# Supplementary material for: NIR-II emissive anionic copper nanoclusters with intrinsic photoredox activity in single-electron transfer
Source: Nat Commun. 2024 Jun 1;15:4688. doi: 10.1038/s41467-024-49081-8 (PMC11144245; doi:10.1038/s41467-024-49081-8)

Supplementary Information

NIR-II emissive anionic copper nanoclusters with intrinsic photoredox activity in single-electron transfer

Li-Juan Liu[1,2†], Mao-Mao Zhang[1†], Ziqi Deng[1], Liang-Liang Yan[1], Yang Lin[1], David Lee Phillips[1], Vivian Wing-Wah Yam[1,3,4] & Jian He[1,3,5]*

[1]Department of Chemistry, The University of Hong Kong, Hong Kong, China.
[2]Chemistry of Chemical Engineering of Guangdong Laboratory, Shantou, China.
[3]State Key Laboratory of Synthetic Chemistry, The University of Hong Kong, Hong Kong, China.
[4]Institute of Molecular Functional Materials, The University of Hong Kong, Hong Kong, China.
[5]HKU Material Innovation Institute for Life Sciences and Energy and The University of Hong Kong Shenzhen Institute of Research and Innovation, Shenzhen, China.
[†]These authors contributed equally: Li-Juan Liu and Mao-Mao Zhang

*email: jianhe@hku.hk

Table of Contents

1. General information..S–2
2. Preparation of Cu_{40} nanoclusters...S–5
3. Supplementary figures and tables...S–6
4. Photoinduced cyanofluoroalkylation of alkenes S–29
5. Supplementary references..S–52
6. NMR spectra S–53

1. General information

Materials and reagents. Unless otherwise noted, materials were purchased from commercial suppliers and used as received.

Instrumentation. X-ray diffraction data of the crystal was collected using synchrotron radiation (λ = 0.67043 Å) on beamline 17B1 at the National Facility for Protein Science Shanghai (NFPS) in the Shanghai Synchrotron Radiation Facility, Shanghai, People's Republic of China. The diffraction data reduction and integration were performed by the HKL3000 software. Powder X-ray diffraction (PXRD) patterns were recorded on a Rigaku Ultima IV X-ray diffractometer (CuKα, λ = 1.5418 Å), operating at 40 kV and 30 mA. The measurement parameters included a scan speed of 10° min^{-1}, a step size of 0.05°, and a scan range of 2θ from 3° to 50°.

Scanning electron microscope (SEM) images and energy dispersive spectroscopy (EDS) mapping were collected on an EM-30 AX PLUS microscope (South Korea, COXEM company).

Electrospray ionization time-of-flight (ESI-TOF) mass spectrometry data were recorded on a Waters Q-TOF mass spectrometer using a Z-spray source. The ESI-TOF mass sample was prepared by dissolving the nanoclusters in CH_2Cl_2 (~0.5 mg mL^{-1}). For the positive-ion or negative-ion mode detection, the samples were directly infused into the chamber at 5 mL min^{-1}, respectively. The source temperature was maintained at 70 °C, the spray voltage was 2.20 kV, and the cone voltage was adjusted to 60 V. High-resolution EI mass spectra were recorded on a Thermo Scientific DFS Magnetic Sector GCHRMS system. High-resolution ESI mass measurements were performed on a Bruker impact II high-resolution LC-QTOF mass spectrometer. Accurate masses from high-resolution mass spectra were reported for the molecular ion $[M+Na]^+$.

All UV−Vis absorption spectra were acquired in the 200–800 nm range using a Cary 3500 spectrophotometer (Agilent). Steady-state emission spectra were obtained on an Edinburgh FLS1000 spectrophotometer.

^{1}H NMR spectra were recorded on a Bruker 500 (500 MHz) or Bruker 400 (400 MHz)

spectrometer in $CDCl_3$. Chemical shifts were quoted in parts per million (ppm) referenced to 0.0 ppm of tetramethyl silane (TMS). ^{13}C NMR spectra were recorded on a Bruker 500 (500 MHz) or Bruker 400 (400 MHz) spectrometer in $CDCl_3$ with complete proton decoupling. Chemical shifts are reported in ppm with the solvent resonance as the internal standard ($^{13}CDCl_3$: 77.00 ppm). ^{19}F and ^{31}P NMR spectra were recorded on a Bruker 500 (500 MHz) or Bruker 400 (400 MHz) spectrometer. The following abbreviations (or combinations thereof) were used to explain multiplicities: s = singlet, d = doublet, t = triplet, q = quartet, m = multiplet. Coupling constants, *J*, were reported in Hertz unit (Hz).

Cyclic voltammograms (CVs) were performed on a CHI760E electrochemistry workstation. Regular 3-electrode systems were used. Measurements were recorded in an acetonitrile solution of Bu_4NClO_4 (0.1 M) at a scan rate of 100 mV s^{-1} under the protection of N_2 using a glassy carbon disk (d = 0.3 cm) as a working electrode and a platinum plate (1 cm × 1 cm) as a counter electrode. An Ag/AgCl (3 M KCl) electrode was used as a reference electrode in all the experiments, and its potential (0.46 V vs. Fc^+/Fc) was calibrated with the ferrocenium/ferrocene (Fc^+/Fc) redox couple.

Stern−Volmer experiments. An Edinburgh FLS-5 spectrophotometer was used for luminescence quenching experiments. Linear regression of I_0/I against concentration was performed in Origin. All samples for the luminescence test were prepared in the glovebox, and the measurements were performed at room temperature. Nanocluster solution (0.025 mM) in acetonitrile was excited at 456 nm and the emission was collected at 1077 nm. For each quenching experiment, a certain volume of the stock solution was added into a 4-mL solution of $[Cu_{40}H_{17}(2,4\text{-DMBT})_{24}](PPh_4)$ (0.025 mM) in a 10-mm quartz cuvette with a screw cap.

Density functional theory calculations. For the experimental complex, $[Cu_{40}H_{17}(SR)_{24}]^-$ (R = 2,4-$Me_2C_6H_3$), the aryl groups were replaced by the –CH_3 in theoretical computations to reduce the computational cost without affecting the interfacial bond strength.

Calculations of UV–Vis absorption spectra and Kohn–Sham (K–S) molecular orbitals: Gaussian 16 package[1] was used to obtain the optimized geometry by Perdew-Burke-Ernzerhof

hybrid functional (PBE0)[2] method with Grimme's BJ-damped variant of DFT-D3 empirical dispersion[3,4]. The pseudopotential basis set LANL2DZ and all-electron def2-SVP were used for Cu atoms and other atoms (H, C, and S), respectively. The time dependent density functional theory method (TDDFT) implemented in Gaussian 16 was used to compute the simulated spectra using the same functional, empirical dispersion and basis sets as above. K–S orbital analysis was performed for identifying the atomic orbital contribution to each molecular orbital using Multiwfn 3.8 program[5,6].

Based on the hydrides assigned by X-ray diffraction data, all possible positions of the hydrides in $[Cu_{40}H_{17}(SR)_{24}]^-$ (R = CH_3) were predicted. The energetically favored structure was then fully optimized without any constraints, and the resulting structure is the predicted final model structure shown in Fig. 3c.

2. Preparation of Cu_{40} nanoclusters

Synthesis of $[Cu_{40}H_{17}(2,4\text{-DMBT})_{24}](PPh_4)$ nanocluster (denoted as Cu_{40}-H). $[Cu(CH_3CN)_4]PF_6$ (50 mg, 0.13 mmol) and PPh_4Br (20 mg, 0.048 mmol) were dissolved in acetonitrile (5 mL). Then, 2,4-dimethylbenzenethiol (2,4-DMBTH) (10 μL, 0.074 mmol) was introduced to the reaction. After stirring for 10 minutes, freshly prepared $NaBH_4$ (50 mg, 1.3 mmol) in an ice-cold methanol solution (5 mL) was added instantaneously. The solvent was evaporated after the reduction for 5 hours, the remaining solid was dissolved in dichloromethane and filtered. Red block-like crystals of **Cu_{40}-H** suitable for single-crystal X-ray analysis were obtained by slow vapor diffusion of hexanes into 5-mL dichloromethane-toluene (1:1, v/v) of the nanoclusters at –4 °C for one week.

Gram-scale synthesis. $[Cu(CH_3CN)_4]PF_6$ (4.65 mg, 12.5 mmol) and PPh_4Br (1.89 g, 4.51 mmol) were dissolved in acetonitrile (450 mL). Then, 2,4-dimethylbenzenethiol (2,4-DMBTH) (0.930 mL, 6.90 mmol) was introduced to the reaction. After stirring for 10 minutes, freshly prepared $NaBH_4$ (4.70 g, 124 mmol) in an ice-cold methanol solution (450 mL) was added instantaneously. The solvent was evaporated after the reduction for 5 hours, the remaining solid was dissolved in dichloromethane and filtered. Red block-like crystals of **Cu_{40}-H** suitable for single-crystal X-ray analysis were obtained by slow vapor diffusion of hexanes into 400-mL dichloromethane-toluene (1:1, v/v) of the nanoclusters at –4 °C for one week.

Synthesis of $[Cu_{40}D_{17}(2,4\text{-DMBT})_{24}](PPh_4)$ nanocluster (denoted as Cu_{40}-D). $[Cu(CH_3CN)_4]PF_6$ (50 mg, 0.13 mmol) and PPh_4Br (20 mg, 0.048 mmol) were dissolved in acetonitrile (5 mL). Then, 2,4-DMBTH (10 μL, 0.074 mmol) was introduced to the reaction. After stirring for 10 minutes, freshly prepared $NaBD_4$ (55 mg, 1.3 mmol) in an ice-cold methanol solution (5 mL) was added instantaneously. The solvent was evaporated after the reduction for 5 hours, the remaining solid was dissolved in dichloromethane and filtered. Red block-like crystals of **Cu_{40}-D** were obtained by slow vapor diffusion of hexanes into 5-mL dichloromethane-toluene (1:1, v/v) of the nanoclusters at –4 °C for one week.

3. Supplementary figures and tables

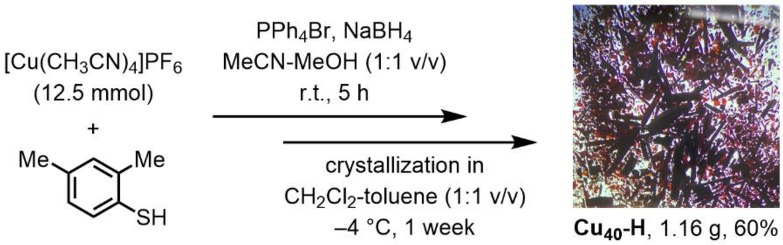

Supplementary Figure 1. One-pot synthetic procedure for **Cu_{40}-H**.

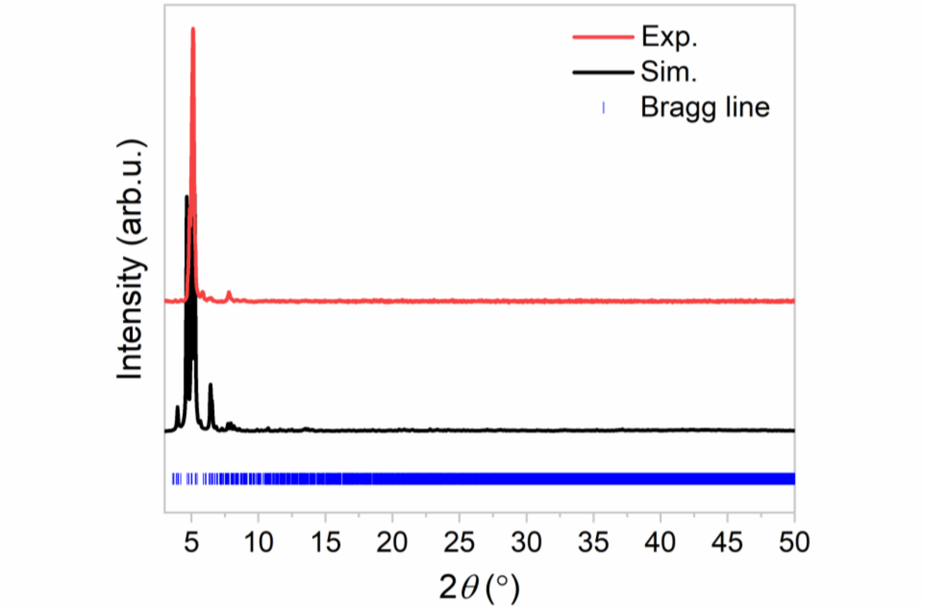

Supplementary Figure 2. PXRD patterns of **Cu_{40}-H**. Source data are provided as a Source Data file.

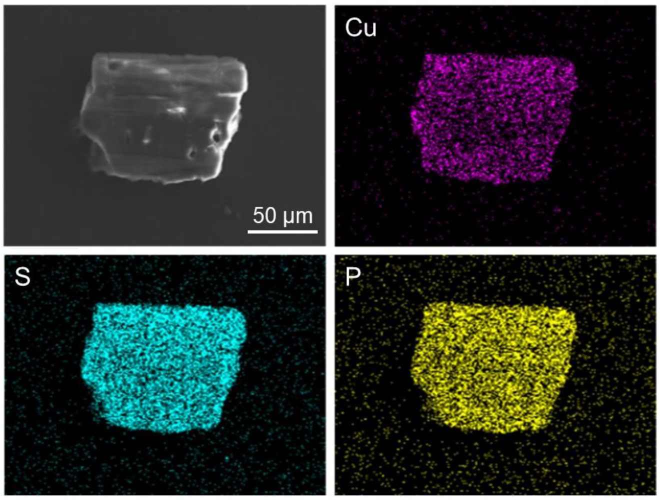

Supplementary Figure 3. SEM image and elemental mapping of **Cu_{40}-H**. EDS mapping images of Cu, S, and P are represented in magenta, cyan, and yellow, respectively. Scale bar, 50 μm.

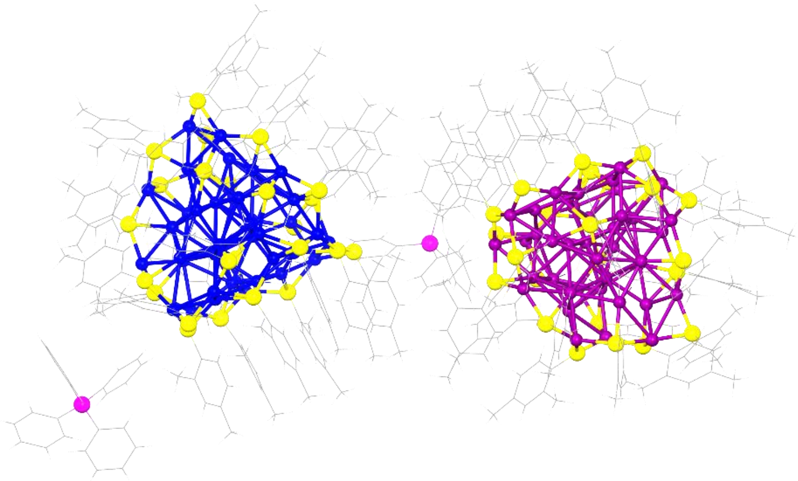

Supplementary Figure 4. One unit cell containing a pair of **Cu_{40}-H** enantiomers. Color labels: Cu, purple and blue; P, magenta; S, yellow; C, gray.

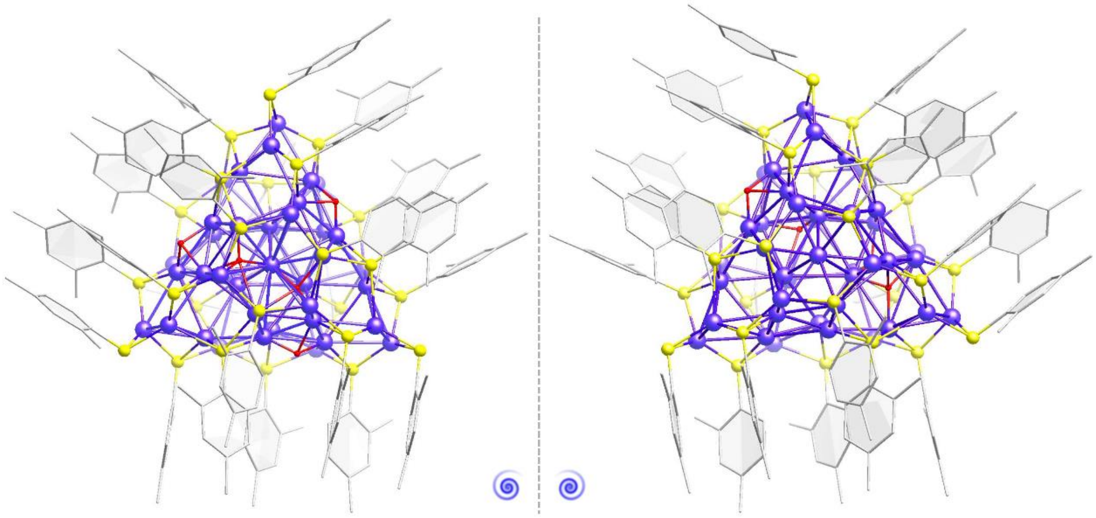

Supplementary Figure 5. Total structure of racemic **Cu_{40}-H** characterized by X-ray crystallography. Color labels: Cu, purple; S, yellow; C, gray; hydrides, red.

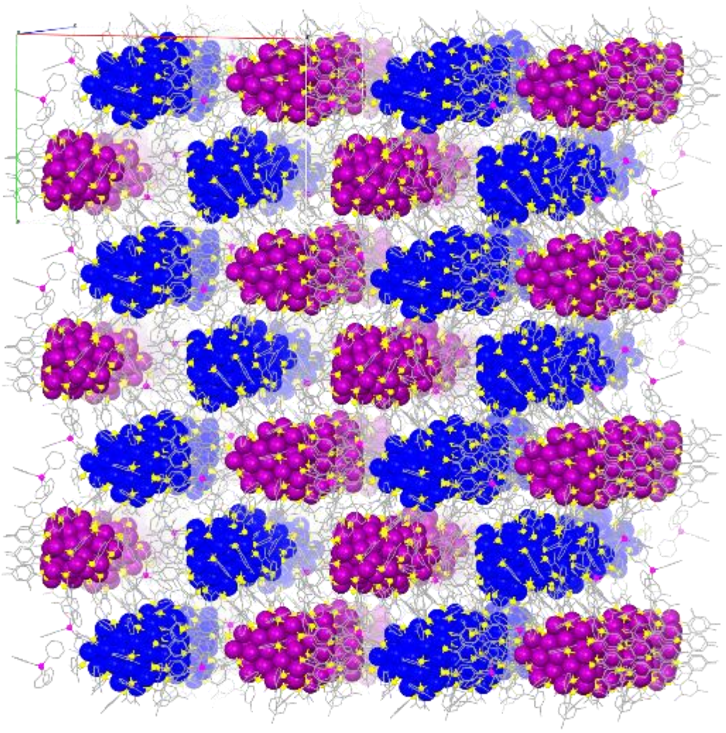

Supplementary Figure 6. Packing mode of **Cu_{40}-H** enantiomers in the $P2_1$/c space group. Color labels: Cu, purple and blue; P, magenta; S, yellow; C, gray.

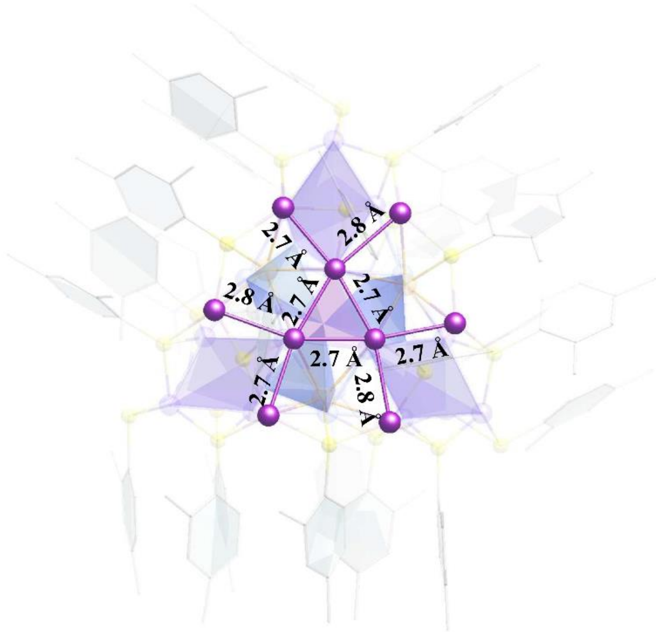

Supplementary Figure 7. Each vertex of the equilateral triangle in the center of the Cu_9 unit is capped by two more copper atoms. Color labels: Cu, purple; S, yellow; C, gray.

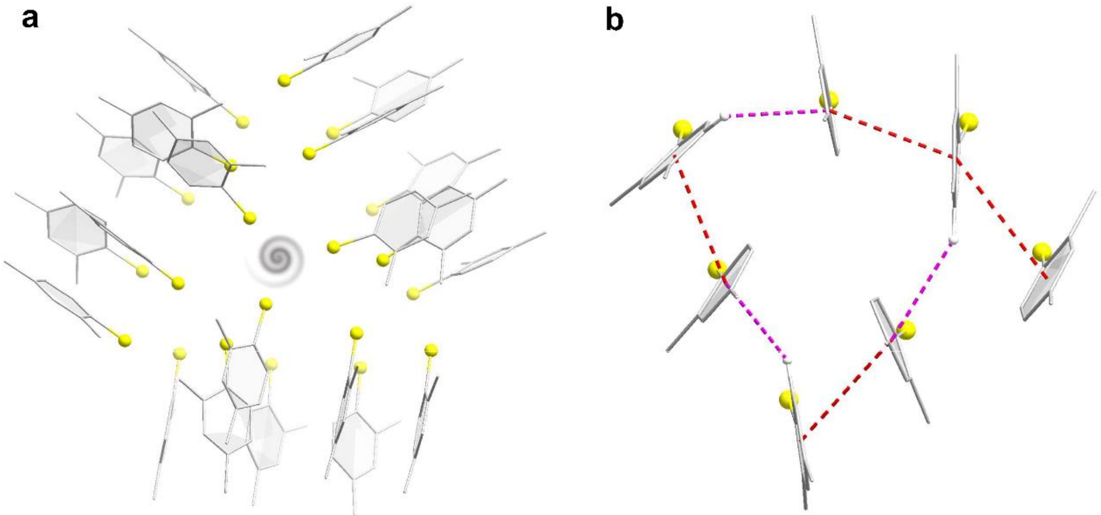

Supplementary Figure 8. Distributions of thiolate groups viewed from the front (**a**) and representation of weak interactions in the ligand assembly (**b**). Color labels: S, yellow; C, gray; H, white. The average distances of π−π stacking and C−H···π interactions are 4.00 and 3.18 Å, respectively.

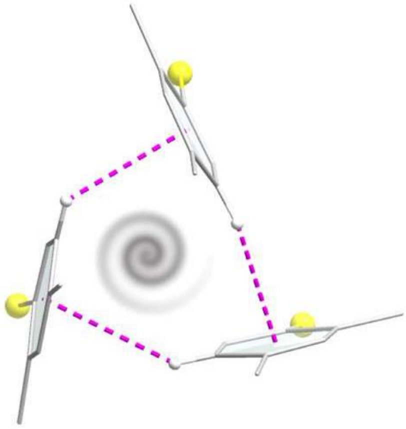

Supplementary Figure 9. Distributions of thiolate groups on the surface of the Cu_9 hexagonal pedestal. Color labels: S, yellow; C, gray; H, white. The average distance of C−H···π interactions is 3.05 Å.

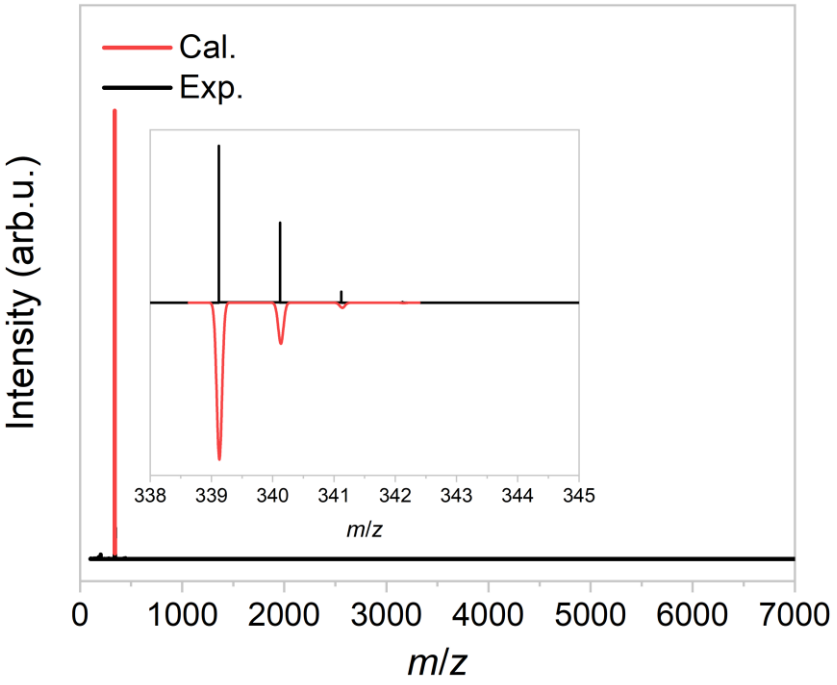

Supplementary Figure 10. ESI-TOF mass spectra of **Cu_{40}-H** in a positive mode. Source data are provided as a Source Data file.

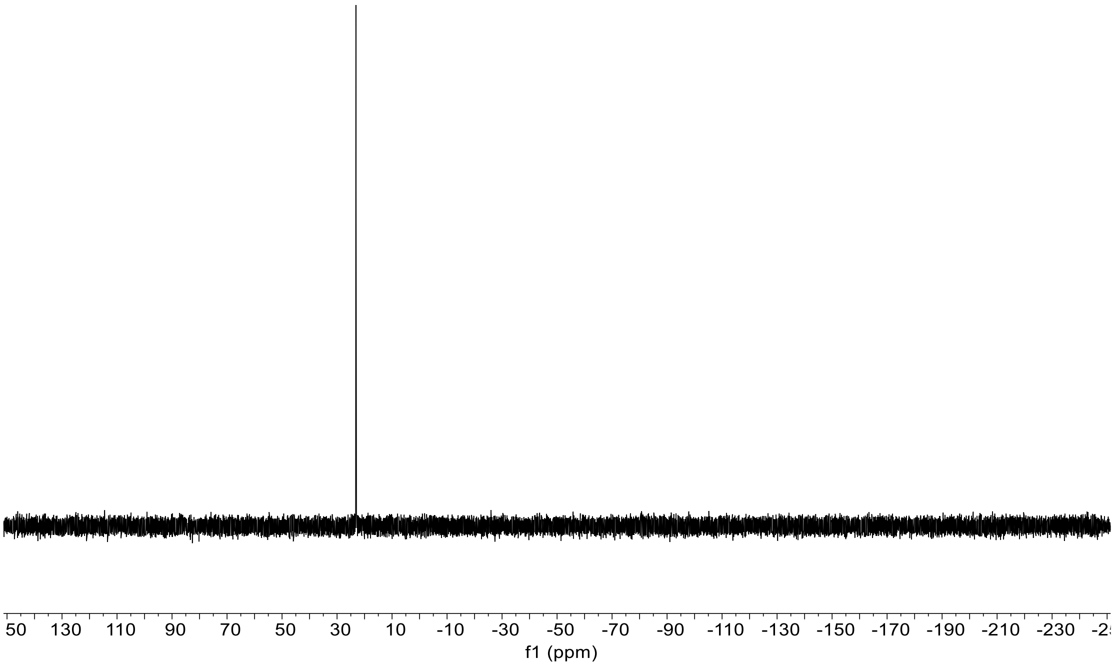

Supplementary Figure 11. ^{31}P NMR spectrum of **Cu_{40}-H** in $CDCl_3$.

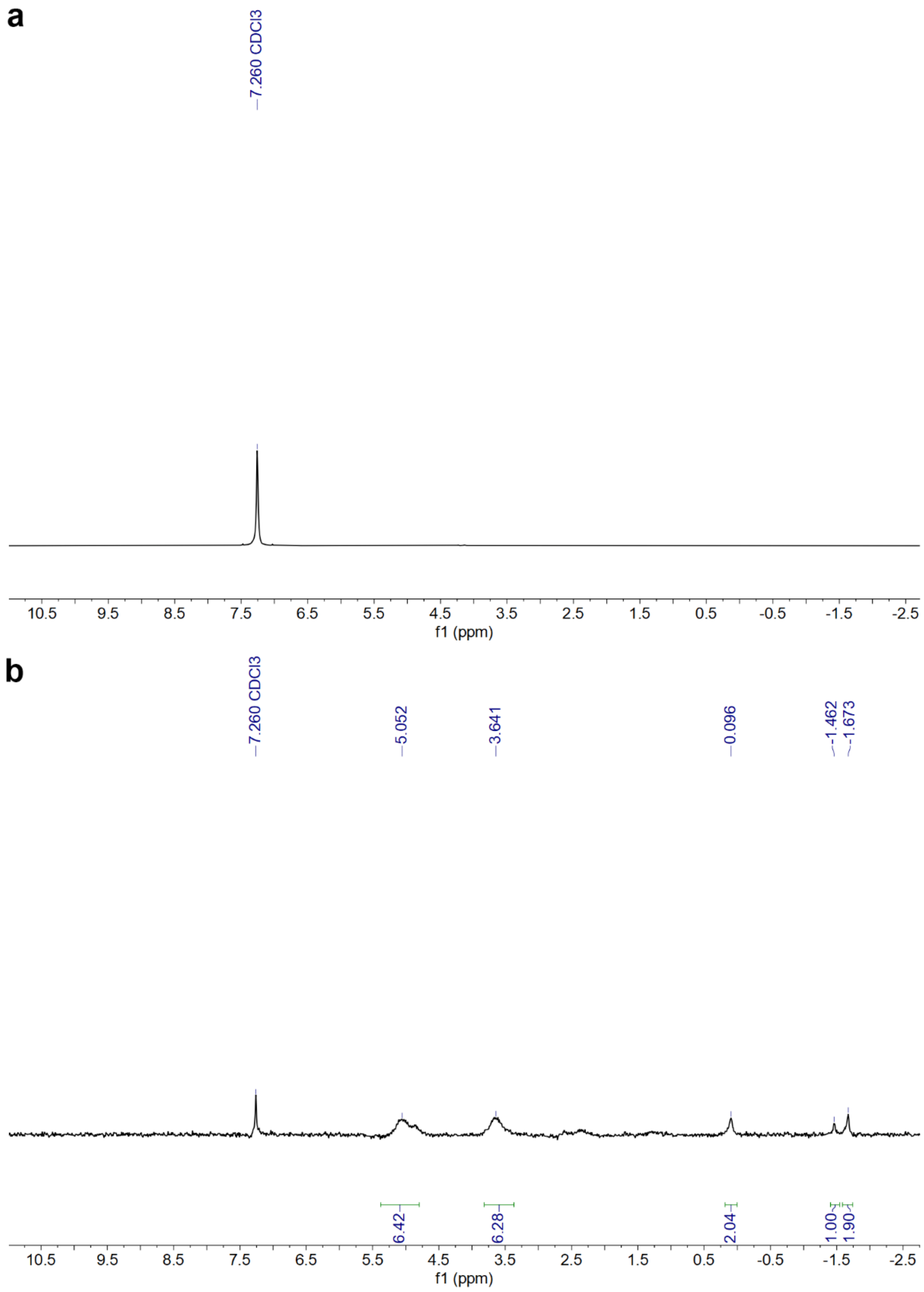

Supplementary Figure 12. ^{2}H NMR spectra of **Cu$_{40}$-H** (**a**) and **Cu$_{40}$-D** (**b**) in $CHCl_3$.

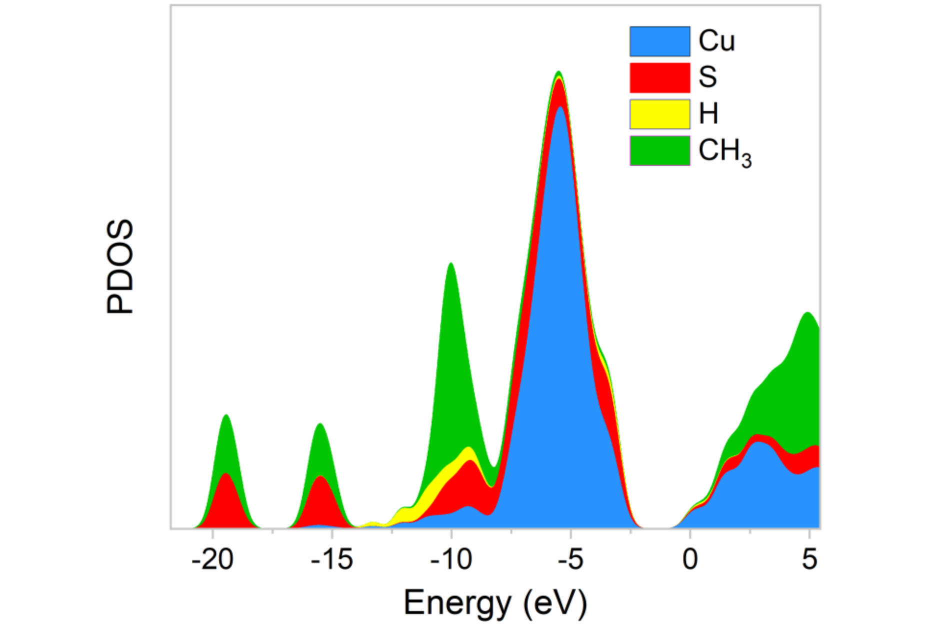

Supplementary Figure 13. PDOS curves for **Cu_{40}-H**.

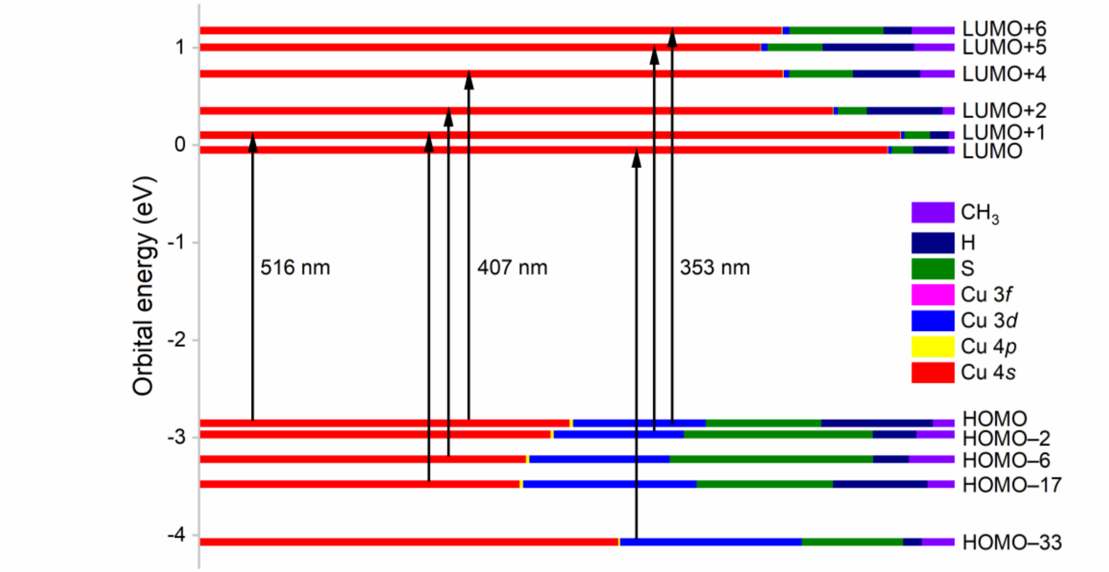

Supplementary Figure 14. Kohn–Sham molecular orbital energy level diagram. Source data are provided as a Source Data file.

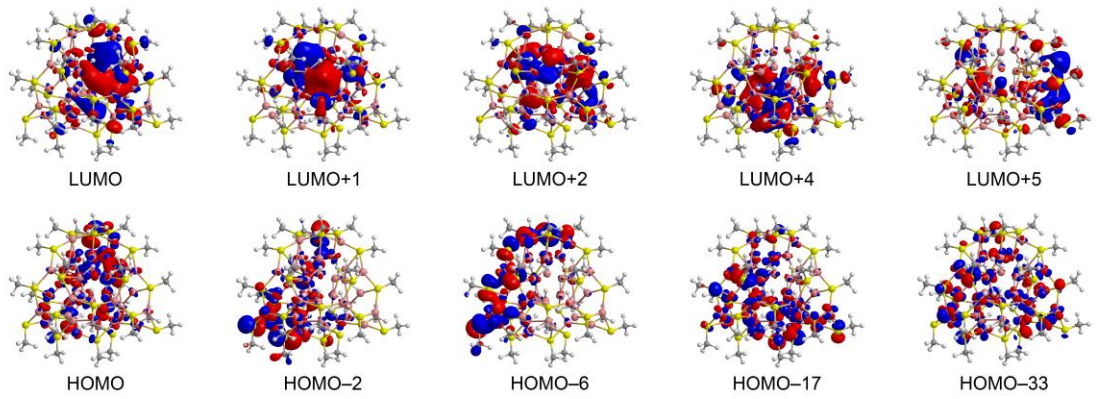

Supplementary Figure 15. Selected frontier molecular orbital of **Cu$_{40}$-H**.

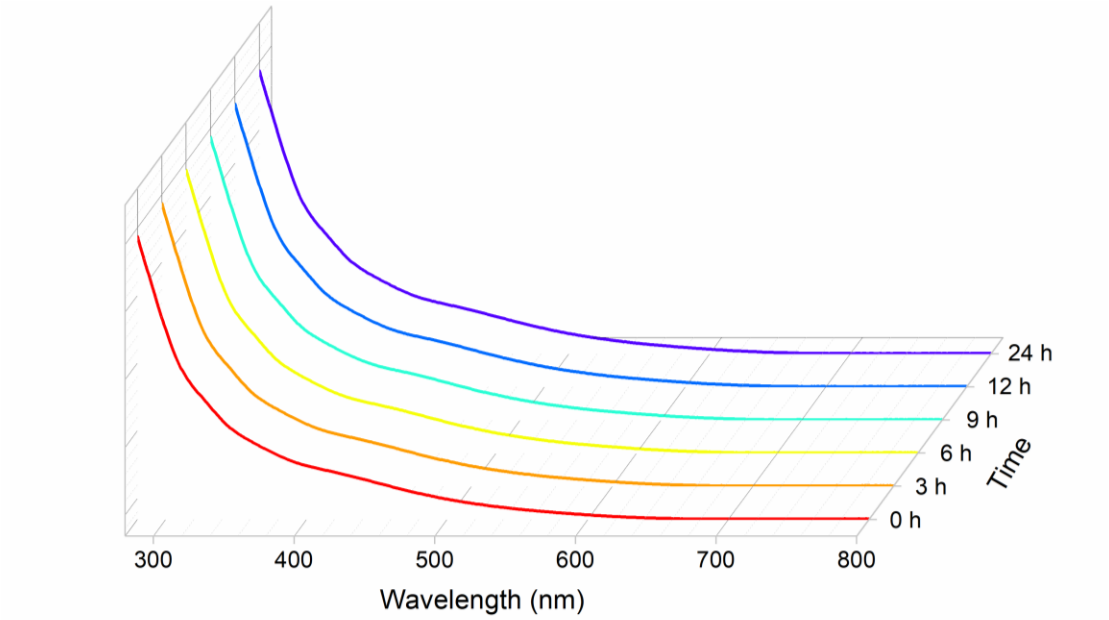

Supplementary Figure 16. Time-dependent UV−Vis absorption spectra of **Cu$_{40}$-H** in dichloromethane. Source data are provided as a Source Data file.

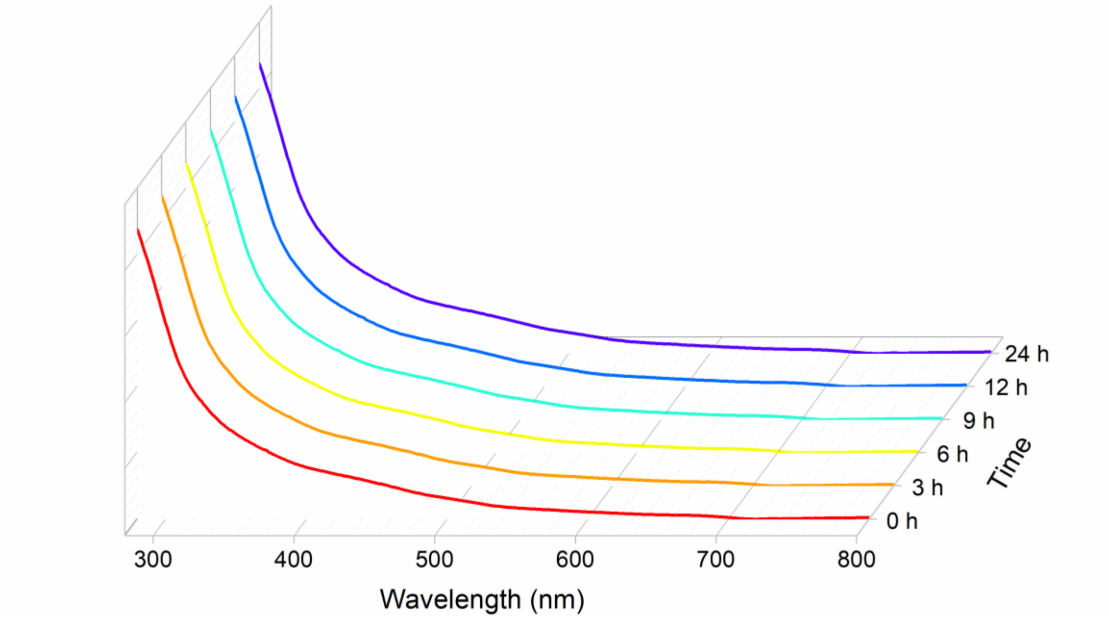

Supplementary Figure 17. Time-dependent UV−Vis absorption spectra of **Cu_{40}-H** in acetonitrile. Source data are provided as a Source Data file.

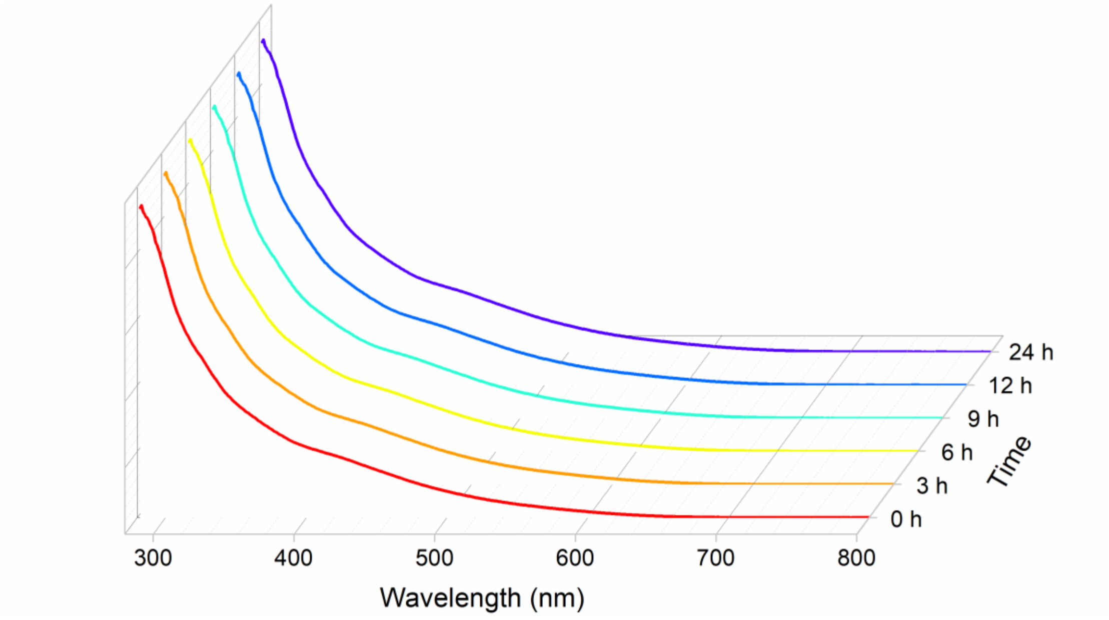

Supplementary Figure 18. Time-dependent UV−Vis absorption spectra of **Cu_{40}-H** in *N*,*N*-dimethylacetamide. Source data are provided as a Source Data file.

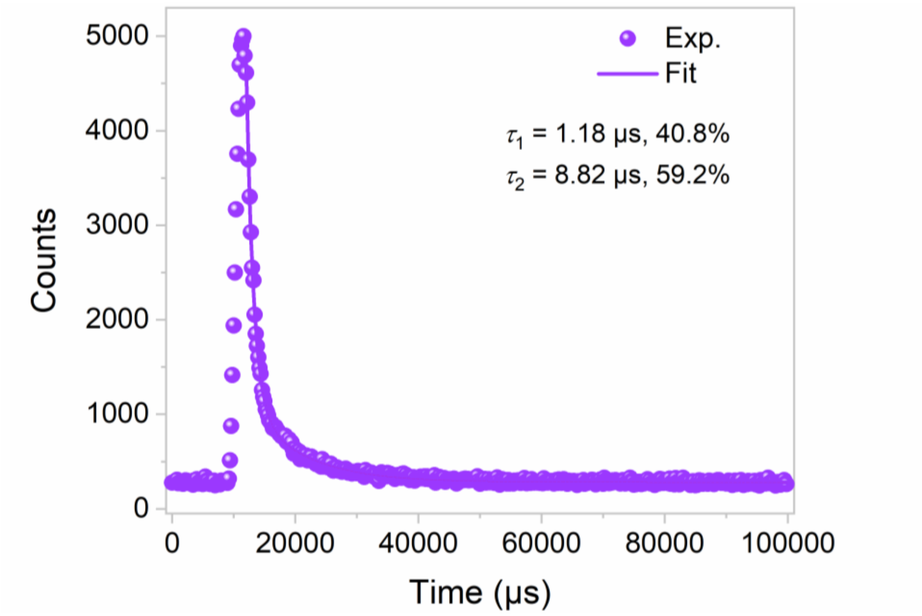

Supplementary Figure 19. Emission decay (λ_{max} = 1174 nm) of photoexcited **Cu_{40}-H** in dichloromethane at room temperature. Source data are provided as a Source Data file.

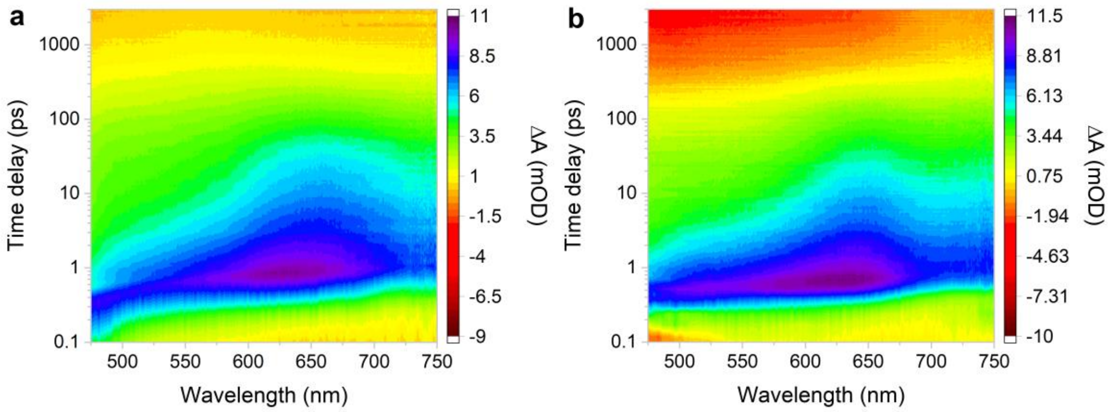

Supplementary Figure 20. TA data maps pumped at 400 nm in toluene (**a**) and *N*,*N*-dimethylacetamide (**b**). Source data are provided as a Source Data file.

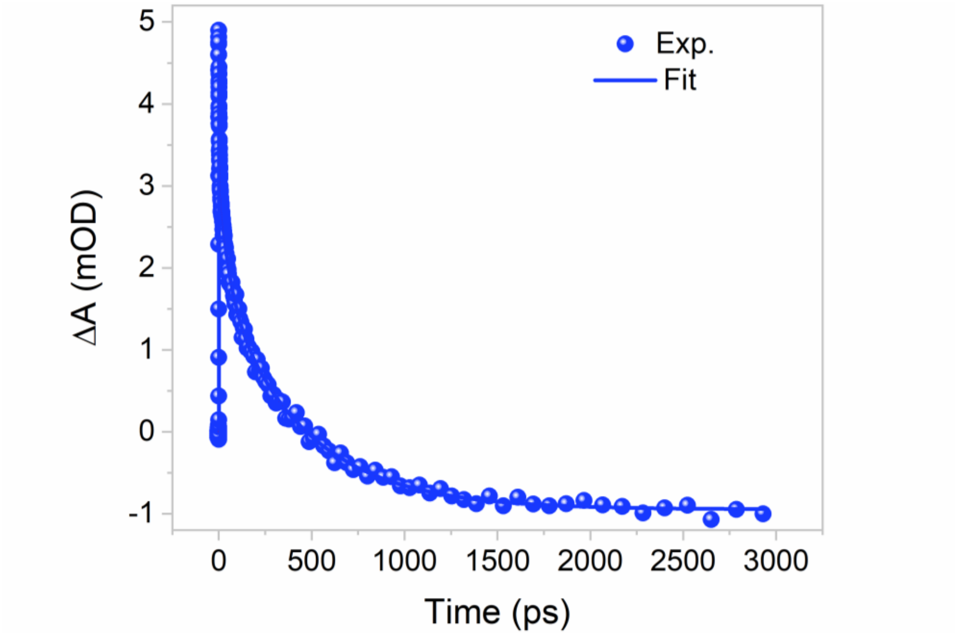

Supplementary Figure 21. TA kinetic traces of **Cu_{40}-H** selected at 650 nm in dichloromethane. Source data are provided as a Source Data file.

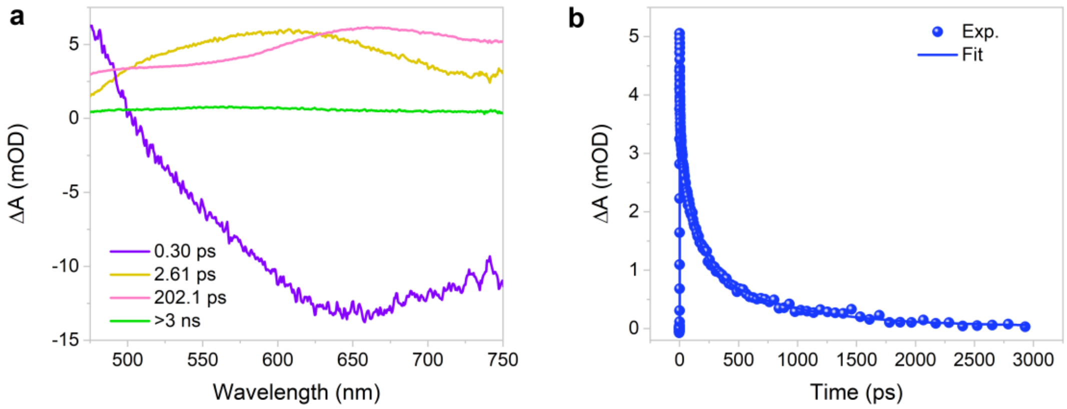

Supplementary Figure 22. a, Species-associated spectra (from global fit analysis). **b**, TA kinetic traces of **Cu_{40}-H** selected at 650 nm in toluene. Source data are provided as a Source Data file.

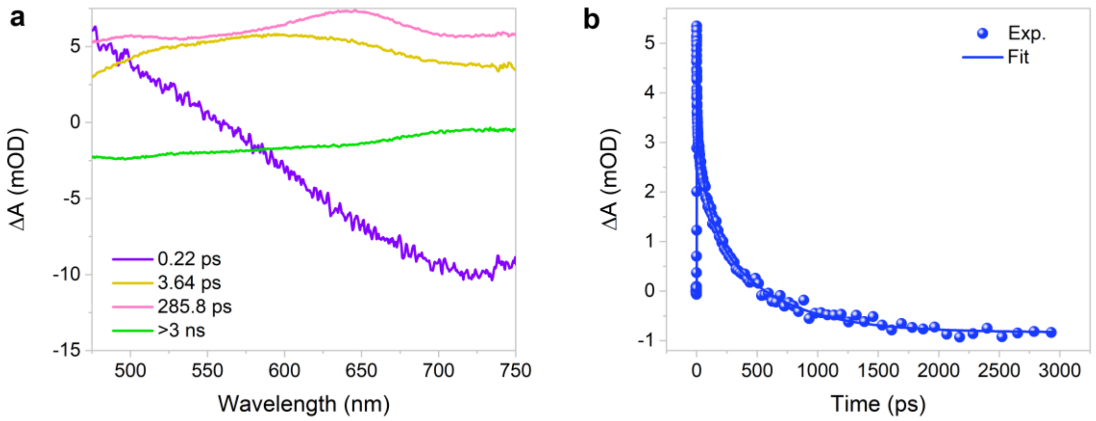

Supplementary Figure 23. a, Species-associated spectra (from global fit analysis). **b**, TA kinetic traces of **Cu_{40}-H** selected at 650 nm in *N*,*N*-dimethylacetamide. Source data are provided as a Source Data file.

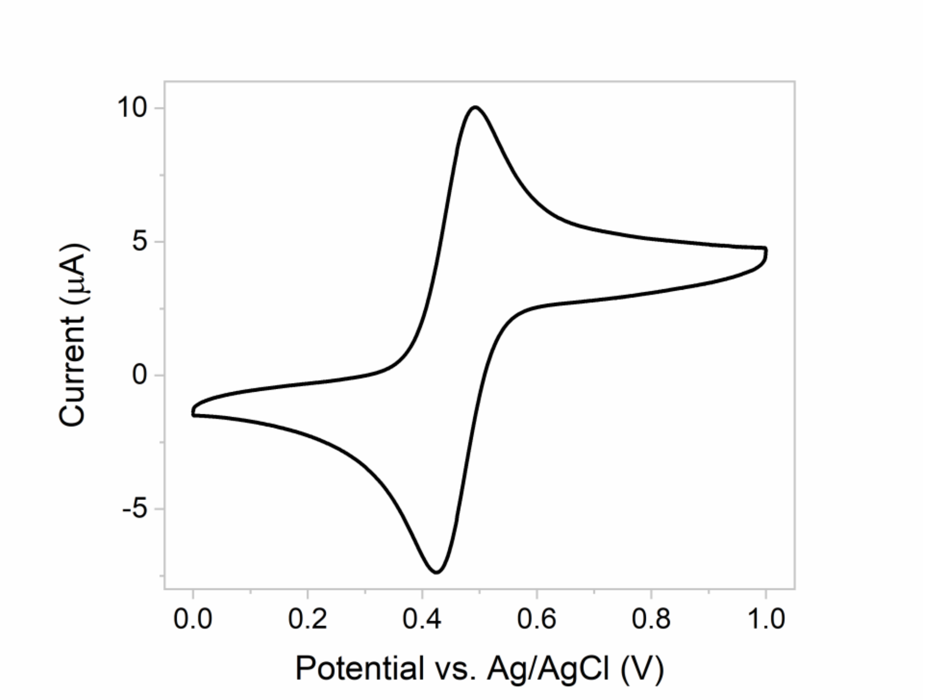

Supplementary Figure 24. CV of ferrocenium [half-wave redox potential $E^0_{1/2}$ (Fc^+/Fc) = 0.46 V]. Source data are provided as a Source Data file.

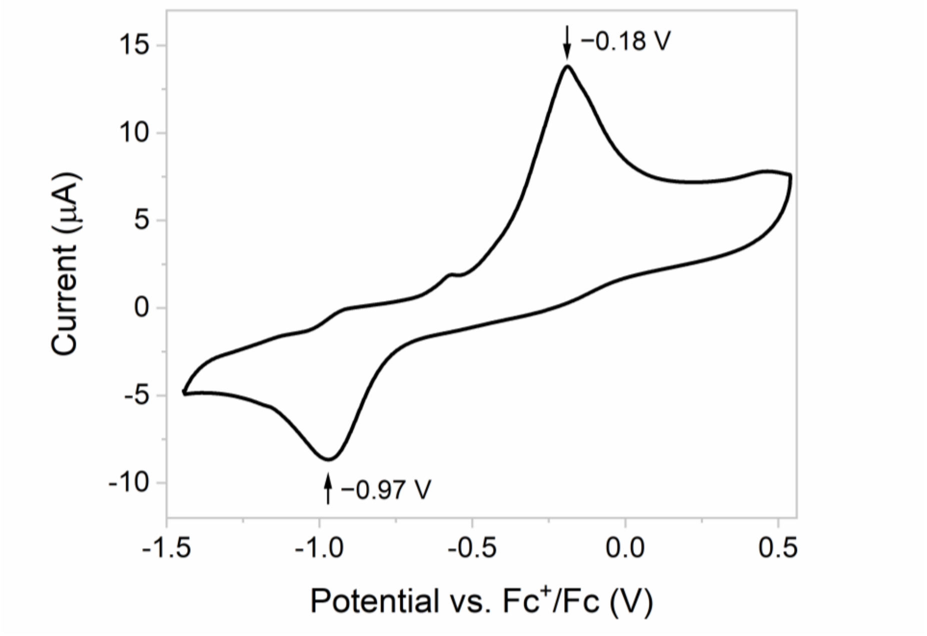

Supplementary Figure 25. CV of **Cu_{40}-H** [$E^0_{1/2}$ (Cu^{II}/Cu^{I}) = –0.58 V vs. Fc^+/Fc]. Source data are provided as a Source Data file.

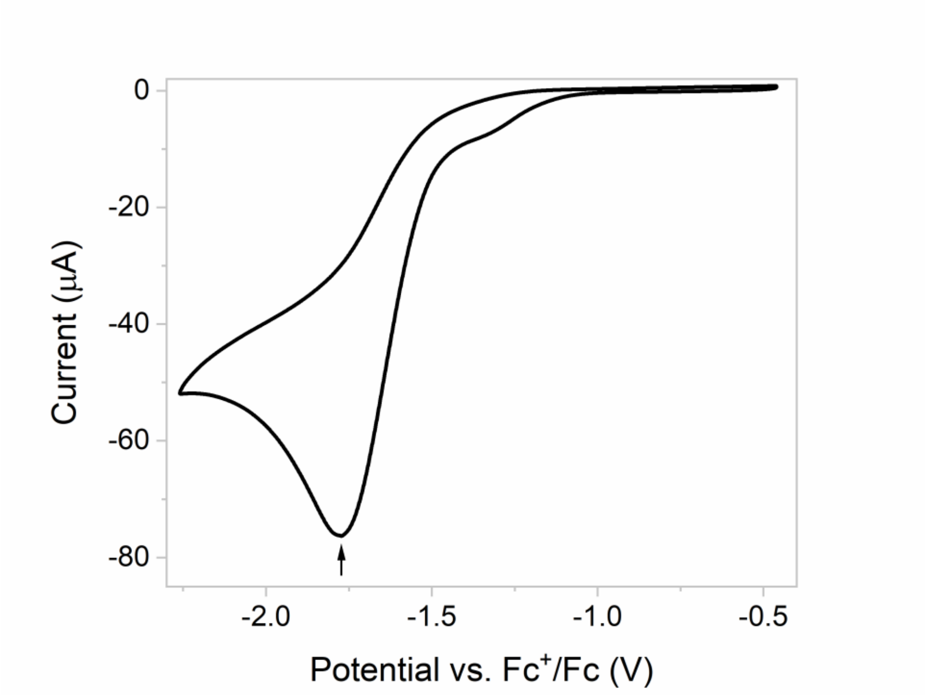

Supplementary Figure 26. CV of C_4F_9I ($E_{p/2}$ = –1.65 V vs. Fc^+/Fc). Source data are provided as a Source Data file.

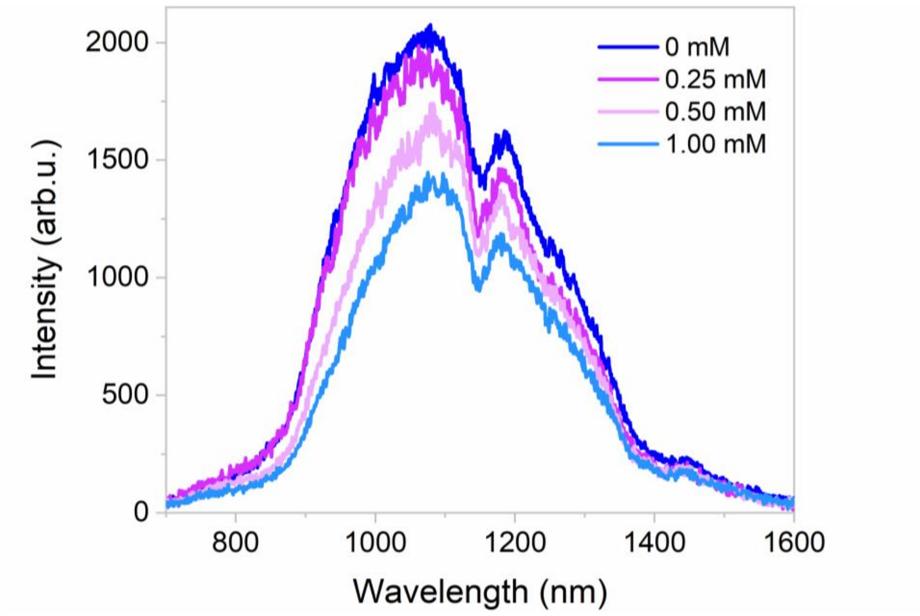

Supplementary Figure 27. Emission spectra of **Cu_{40}-H** (0.025 mM in acetonitrile) with addition of C_4F_9I under nitrogen atmosphere at room temperature (Ex: 456 nm). Source data are provided as a Source Data file.

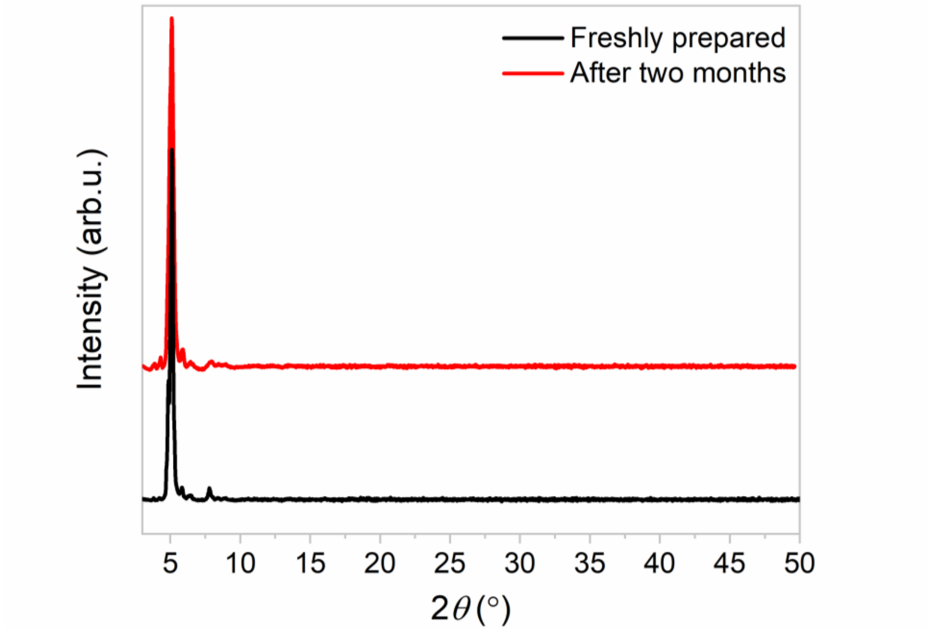

Supplementary Figure 28. Stability test based on the PXRD patterns of **Cu_{40}-H**. Source data are provided as a Source Data file.

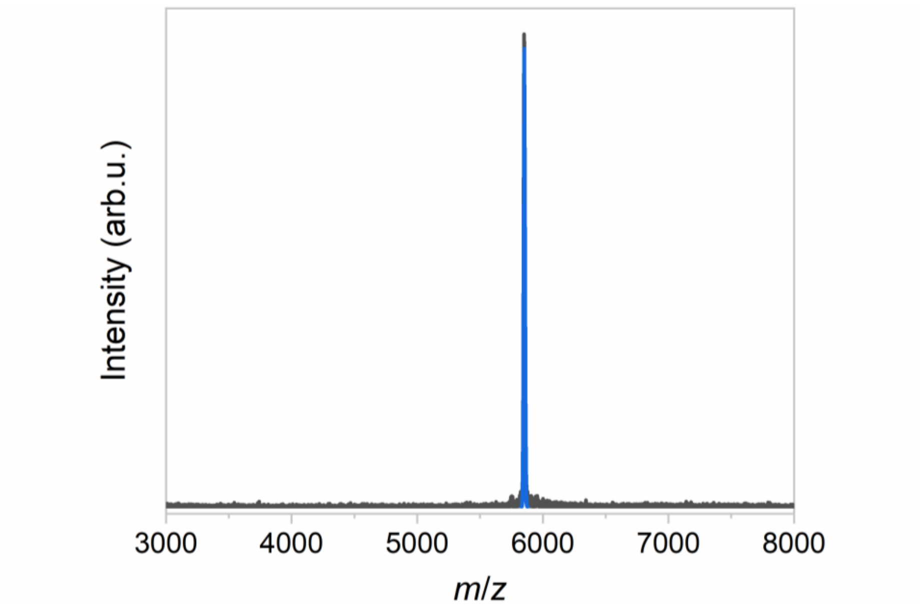

Supplementary Figure 29. ESI-TOF mass spectrum (negative mode) of the reaction mixture upon irradiation for 2 hours. Source data are provided as a Source Data file.

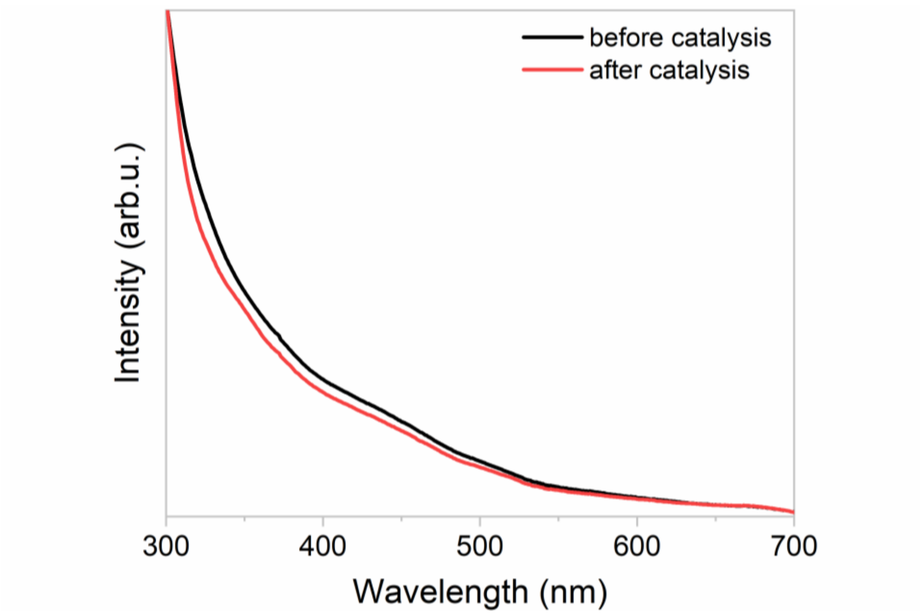

Supplementary Figure 30. UV−Vis absorption spectra of **Cu_{40}-H** before and after photocatalysis. Source data are provided as a Source Data file.

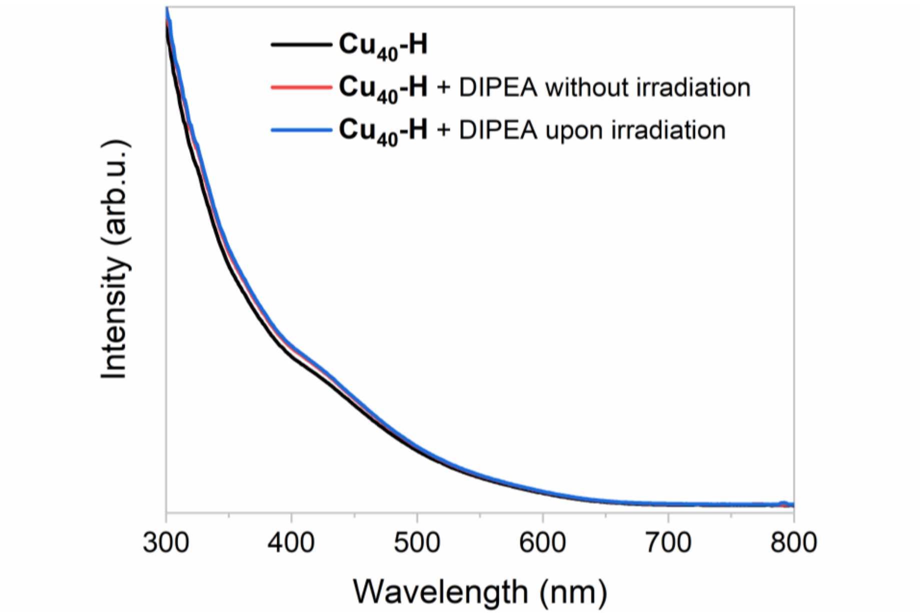

Supplementary Figure 31. Photostability of **Cu_{40}-H** in the presence of *N*,*N*-diisopropylethylamine (DIPEA). Source data are provided as a Source Data file.

Table S1. Crystal data and structure refinement for Cu_{40}-H

Identification code	**Cu_{40}-H**
CCDC number	2263793
Empirical formula	$C_{454}H_{521}Cl_{10}Cu_{80}P_2S_{48}$
Formula weight	6814.59
Temperature/K	293
Crystal system	monoclinic
Space group	$P2_1/c$
a/Å	48.300(10)
b/Å	27.455(5)
c/Å	44.930(9)
α/°	90
β/°	109.02
γ/°	90
Volume/Å^3	56326(22)
Z	4
ρ_{calc} g/cm^3	1.607
μ/mm^{-1}	2.784
F(000)	26124.0
Crystal size/mm^3	0.05 × 0.05 × 0.05
Radiation	synchrotron (λ = 0.67043 Å)
2θ range for data collection/°	1.728 to 52.304
Index ranges	$-63 \le h \le 62$, $0 \le k \le 36$, $-59 \le l \le 58$
Reflections collected	230661
Independent reflections	131836 [R_{int} = 0.0516, R_{sigma} = 0.0552]
Data/restraints/parameters	131836/254/5664
Goodness-of-fit on F^2	1.025
Final R indexes [$I \ge 2\sigma(I)$]	R_1 = 0.0761, wR_2 = 0.1955
Final R indexes [all data]	R_1 = 0.0861, wR_2 = 0.2014
Largest diff. peak/hole/eÅ^{-3}	3.16/−2.80

$R_1 = \sum||F_o|-|F_c||/\sum|F_o|$. $wR_2 = [\sum w(F_o^2-F_c^2)^2/\sum w(F_o^2)^2]^{1/2}$

Table S2. Coordinates of the optimized geometries

Atom	x	y	z
Cu	0.01118	−0.00254	1.62268
Cu	1.35475	−2.52601	1.9223
Cu	0.20195	1.61978	−0.51161
Cu	2.18517	0.06253	3.1069
Cu	−1.48746	−0.62894	−0.52323
Cu	1.50227	2.40924	1.94357
Cu	−1.03679	−1.90715	3.05838
Cu	1.32549	−0.9811	−0.5177
Cu	−1.17774	1.80725	3.09763
Cu	1.18259	−1.93511	4.5656
Cu	−0.76684	−2.9151	0.64107
Cu	3.91891	−3.13403	1.82098
Cu	1.6987	−3.55005	−0.30535
S	2.6557	−3.44231	3.68311
Cu	−2.16919	2.11997	0.6556
Cu	2.23408	3.24398	−0.28032
Cu	−0.66238	4.06717	−0.16916
Cu	2.50632	1.05545	−1.8576
Cu	−2.17112	1.64979	−1.85515
Cu	0.33851	1.53029	−3.22436
Cu	2.926	0.79242	0.66923
Cu	1.03599	1.97478	4.59629
S	4.1597	−0.62921	2.05822
S	2.02168	0.08419	5.41982
Cu	−0.32261	−2.66628	−1.8748
Cu	−3.93346	0.25725	−0.36531
Cu	−1.50938	−0.43613	−3.22662
Cu	−3.20982	−2.59502	−0.23667
Cu	0.74588	4.93761	1.86911
S	1.63397	3.97736	3.72726
Cu	−2.30905	−0.15235	4.57655
S	−2.6158	−3.26287	2.01229
S	−0.96455	−1.92429	5.38554

Cu	3.89332	−1.45048	−0.18649
Cu	1.13237	−1.02223	−3.25374
Cu	−2.88925	0.07315	1.90161
S	−1.1635	1.65041	5.40743
S	−1.52782	3.86627	2.05528
Cu	4.47168	−3.94989	−0.73963
S	5.60849	−4.44398	1.09725
S	2.70098	−4.77594	−1.87733
Cu	1.22386	5.84837	−0.6674
S	2.85036	4.76915	−1.80651
S	−0.73053	5.56908	−1.86533
Cu	2.54074	3.11914	−3.35479
Cu	3.98887	−0.71181	−3.21972
S	4.07906	1.49252	−3.60018
Cu	−1.3428	3.78959	−3.20176
Cu	−3.96852	0.6827	−3.37733
S	−3.3194	2.82667	−3.59682
S	0.55863	3.48085	−4.41517
Cu	−2.61937	−3.01887	−3.20396
Cu	1.42371	−3.68406	−3.43319
S	−0.75799	−4.21652	−3.62683
Cu	−5.71339	−1.91191	−0.72799
S	−5.58107	0.04196	−1.89472
S	−3.28198	−1.22675	−4.4476
Cu	−4.72654	−1.80228	1.7702
S	−4.475	−3.46463	−1.92416
S	1.05664	7.065	1.18287
S	−4.33369	−0.59757	3.66783
S	5.24571	−2.11119	−1.89255
S	2.72486	−2.12856	−4.45226
S	−6.72515	−2.63532	1.11479
H	0.68084	1.17153	2.69457
H	−1.38699	−0.01197	2.6212
H	0.55501	−3.83283	0.97986
H	−1.30188	2.51533	−0.70052
H	3.04311	2.38775	1.02191

H	−3.62182	1.42213	0.9458
H	0.03933	0.02306	−3.92759
H	0.67474	−1.20858	2.68508
H	2.83927	−0.14395	−0.76036
H	−1.53736	−2.39655	−0.78285
Pb	2.38911	−2.13968	0.47086
H	−2.99863	−0.9649	0.38388
H	0.67827	3.15825	0.52126
H	1.14005	2.06408	−1.83922
H	−1.241	0.31394	−1.8371
H	1.21799	−1.97891	−1.8588
H	0.02432	−0.01834	−0.04058
C	−1.95747	6.79243	−1.45772
H	−1.57597	7.43305	−0.66156
H	−2.15423	7.40173	−2.33951
H	−2.88675	6.32826	−1.1242
C	2.66961	7.58087	1.73086
H	2.99165	8.45345	1.15991
H	2.62941	7.8544	2.78718
H	3.41526	6.79302	1.6096
C	−4.55736	3.88237	−2.87598
H	−4.37245	4.91206	−3.17896
H	−5.54764	3.58066	−3.21361
H	−4.5159	3.82117	−1.78643
C	−6.88141	1.22093	−1.59702
H	−7.23666	1.61388	−2.54904
H	−7.70842	0.71564	−1.09968
H	−6.53695	2.04028	−0.96618
C	−7.93286	−1.43762	1.63948
H	−8.86154	−1.57693	1.08335
H	−8.14362	−1.56982	2.70262
H	−7.58687	−0.414	1.48561
C	−4.79474	−5.16216	−1.49466
H	−5.53588	−5.20016	−0.69552
H	−5.18745	−5.6787	−2.3697
H	−3.88426	−5.65831	−1.15715

C	−2.68041	−4.98297	2.4655
H	−3.49263	−5.45813	1.91794
H	−1.73771	−5.46512	2.20767
H	−2.86694	−5.07409	3.53294
C	2.42936	−6.5294	−1.73682
H	2.56511	−6.9856	−2.71728
H	3.15705	−6.95886	−1.04929
H	1.42742	−6.7486	−1.36908
C	−0.98536	−5.83436	−2.9198
H	−1.96374	−6.2212	−3.20268
H	−0.21297	−6.50629	−3.28913
H	−0.92682	−5.78669	−1.8297
C	−2.78414	−1.20836	−6.15633
H	−2.04711	−1.99329	−6.32749
H	−3.66205	−1.38855	−6.77436
H	−2.34014	−0.24807	−6.41698
C	2.46424	−1.7679	−6.17581
H	3.01377	−2.49191	−6.7757
H	1.40335	−1.81668	−6.42048
H	2.82747	−0.76295	−6.39437
C	0.4052	3.12063	−6.15164
H	−0.648	3.00008	−6.40683
H	0.82219	3.95139	−6.71917
H	0.93412	2.20078	−6.40212
C	5.61141	2.03927	−2.87875
H	6.41993	1.39073	−3.2139
H	5.82311	3.06308	−3.1828
H	5.55533	1.9943	−1.78852
C	4.51748	5.34461	−1.56667
H	4.92791	5.65042	−2.52885
H	4.51099	6.20713	−0.90118
H	5.14727	4.5694	−1.13084
C	−2.93456	4.87539	2.47222
H	−3.84497	4.42143	2.08032
H	−3.00934	4.98215	3.55298
H	−2.79442	5.85741	2.0236

C	−2.17796	3.02125	5.91722
H	−2.37688	2.93167	6.98472
H	−1.6501	3.95776	5.72762
H	−3.12359	3.03764	5.37457
C	3.33602	4.44661	3.95596
H	3.42338	5.52782	3.85615
H	3.66287	4.15728	4.95451
H	3.97666	3.96431	3.21642
C	3.71731	0.21643	5.94511
H	3.73988	0.32898	7.02858
H	4.2614	−0.68785	5.66554
H	4.20599	1.07496	5.48354
C	5.70518	0.14975	2.47393
H	5.84785	0.1343	3.55302
H	6.51091	−0.4054	1.99656
H	5.70747	1.17859	2.11416
C	5.3319	−6.13991	1.56167
H	5.92485	−6.79482	0.92124
H	5.64338	−6.29443	2.59665
H	4.28256	−6.42685	1.47224
C	6.9251	−1.69693	−1.47279
H	7.26761	−2.36045	−0.67752
H	7.55248	−1.84062	−2.3519
H	7.00882	−0.66342	−1.13392
C	2.3303	−5.18315	3.86177
H	3.27941	−5.71756	3.84811
H	1.8311	−5.37213	4.81195
H	1.70213	−5.54799	3.04777
C	−1.52924	−3.58225	5.70258
H	−1.33726	−3.8211	6.74841
H	−2.60234	−3.65297	5.51408
H	−1.01621	−4.30577	5.06894
C	−5.54345	0.67638	3.95369
H	−6.53992	0.27867	3.76442
H	−5.48276	0.99885	4.99253
H	−5.36986	1.53294	3.30065

Table S3. Analysis of selected excited states with largest oscillator strengths that contributes to the peaks in the theoretical UV−Vis spectrum of Cu_{40}-H

Orbital energy (eV)	Cu(s)	Cu(p)	Cu(d)	Cu(f)	S	H	R(CH_3)
−4.0719	0.5547	0.0025	0.2404	2.9659E−6	0.1339	0.0250	0.0434
−3.4773	0.4241	0.2297	0.2297	4.2604E−6	0.1803	0.1253	0.0359
−3.2226	0.4327	0.1856	0.1856	5.2506E−6	0.2692	0.0474	0.0606
−2.9677	0.4650	0.1728	0.1728	4.2883E−6	0.250	0.0578	0.0506
−2.8526	0.4902	0.1756	0.1756	4.5082E−6	0.1532	0.1474	0.0289
−0.0501	0.9109	0.0051	0.0051	5.1587E−7	0.0283	0.0464	0.0084
0.1023	0.9279	0.0049	0.0049	5.2111E−7	0.0335	0.0252	0.0077
0.3518	0.8384	0.0056	0.0056	7.1439E−7	0.0384	0.0100	0.0165
0.7290	0.7727	0.0070	0.0070	9.0547E−7	0.0844	0.089	0.0457
1.0027	0.7428	0.0088	0.0088	1.1017E−6	0.0727	0.1209	0.0537
1.1747	0.7710	0.0091	0.0091	1.7305E−6	0.1246	0.0374	0.0568

Table S4. Investigation of visible-light-induced two-component coupling via the formation of an electron donor−acceptor complex

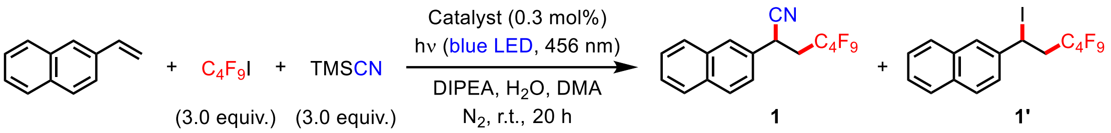

Entry	Catalyst	Yield of **1** (%)	Yield of **1’** (%)
1	**Cu_{40}-H**	78	<5
2	no catalyst	<5	9

Standard conditions: 2-vinylnaphthalene (0.10 mmol, 1.0 equiv.), C_4F_9I (3.0 equiv.), TMSCN (3.0 equiv.), **Cu_{40}-H** (0.3 mol%) or without catalyst, DIPEA (4.0 equiv.), and H_2O (2.0 equiv.) in anhydrous DMA (1.0 mL) under nitrogen atmosphere at room temperature with blue-LED light irradiation (456 nm) for 20 h. Yield was determined by ^{1}H NMR of the crude product using 1,2-dibromoethane as an internal standard.

4. Photoinduced cyanofluoroalkylation of alkenes

General procedure. To a 10-mL flame-dried Schlenk tube under N_2 atmosphere were added **Cu_{40}-H** (2.0 mg, 0.3 mol%), alkene (0.10 mmol, 1.0 equiv.), fluoroalkyl iodide (0.30 mmol, 3.0 equiv.), TMSCN (29.8 mg, 0.30 mmol, 3.0 equiv.), DIPEA (69.7 μL, 0.40 mmol, 4.0 equiv.), H_2O (3.6 μL, 0.20 mmol, 2.0 equiv.), and anhydrous DMA (1.0 mL) sequentially. The reaction mixture was irradiated by 40-watt Kessil PR160L-456 blue-LED lamps at room temperature for 20 hours. After irradiation, the reaction mixture was diluted with saturated brine (10 mL) and extracted four times with ethyl acetate (4 × 5 mL). The combined organic layers were dried over anhydrous Na_2SO_4 and concentrated under vacuum. The residue was purified by flash column chromatography on silica gel to afford the corresponding product.

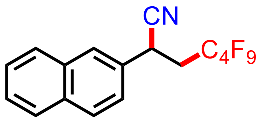

4,4,5,5,6,6,7,7,7-Nonafluoro-2-(naphthalen-2-yl)heptanenitrile (1)[7]**.** The title compound was synthesized according to the General Procedure using 2-vinylnaphthalene (15.4 mg, 0.10 mmol) and C_4F_9I (103.8 mg, 0.30 mmol). The crude mixture was purified by flash column chromatography using hexanes/ethyl acetate (30:1 v/v) as the eluent to give 30.3 mg (0.076 mmol, 76% yield) of the title compound as a colorless oil.

^{1}H NMR (400 MHz, $CDCl_3$) δ 7.96 – 7.75 (m, 4H), 7.62 – 7.51 (m, 2H), 7.43 (dd, J = 8.5, 2.0 Hz, 1H), 4.37 (dd, J = 9.8, 4.4 Hz, 1H), 3.02 – 2.83 (m, 1H), 2.73 – 2.55 (m, 1H).

^{19}F NMR (376 MHz, $CDCl_3$) δ −81.0 – −80. 8 (m, 3F), −112.3 – −114.8 (m, 2F), −124.1 – −124.4 (m, 2F), −125.7 – −125.9 (m, 2F).

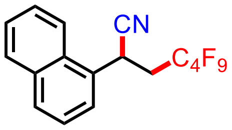

4,4,5,5,6,6,7,7,7-Nonafluoro-2-(naphthalen-1-yl)heptanenitrile (2)[8]. The title compound was synthesized according to the General Procedure using 1-vinylnaphthalene (15.4 mg, 0.10 mmol) and C_4F_9I (103.8 mg, 0.30 mmol). The crude mixture was purified by flash column chromatography using hexanes/ethyl acetate (30:1 v/v) as the eluent to give 28.7 mg (0.072 mmol, 72% yield) of the title compound as a colorless oil.

^{1}H NMR (400 MHz, $CDCl_3$) δ 8.00 – 7.90 (m, 2H), 7.86 (d, *J* = 8.5 Hz, 1H), 7.81 – 7.77 (m, 1H), 7.69 – 7.64 (m, 1H), 7.62 – 7.46 (m, 2H), 4.96 (dd, *J* = 10.6, 3.2 Hz, 1H), 3.02 – 2.83 (m, 1H), 2.79 – 2.57 (m, 1H).

^{19}F NMR (376 MHz, $CDCl_3$) δ −80.8 – −80.0 (m, 3F), −112.4 – −116.3 (m, 2F), −124.1 – −124.4 (m, 2F), −125.7 – −125.9 (m, 2F).

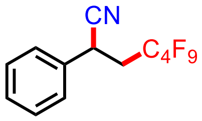

4,4,5,5,6,6,7,7,7-Nonafluoro-2-phenylheptanenitrile (3)[7]. The title compound was synthesized according to the General Procedure using styrene (10.4 mg, 0.10 mmol) and C_4F_9I (103.8 mg, 0.30 mmol). The crude mixture was purified by flash column chromatography using hexanes/ethyl acetate (30:1 v/v) as the eluent to give 32.8 mg (0.094 mmol, 94% yield) of the title compound as a colorless oil.

^{1}H NMR (400 MHz, $CDCl_3$) δ 7.50 – 7.36 (m, 5H), 4.19 (dd, *J* = 9.9, 4.5 Hz, 1H), 2.93 – 2.78 (m, 1H), 2.67 – 2.46 (m, 1H).

^{19}F NMR (376 MHz, $CDCl_3$) δ −80.8 – −81.0 (m, 3F), −111.3 – −115.9 (m, 2F), −124.2 – −124.4 (m, 2F), −125.7 – −126.0 (m, 2F).

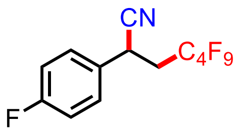

4,4,5,5,6,6,7,7,7-Nonafluoro-2-(4-fluorophenyl)heptanenitrile (4)[7]**.** The title compound was synthesized according to the General Procedure using 1-fluoro-4-vinylbenzene (12.2 mg, 0.10 mmol) and C_4F_9I (103.8 mg, 0.30 mmol). The crude mixture was purified by flash column chromatography using hexanes/ethyl acetate (30:1 v/v) as the eluent to give 32.7 mg (0.089 mmol, 89% yield) of the title compound as a colorless oil.

^{1}H NMR (400 MHz, $CDCl_3$) δ 7.38 (dd, J = 8.4, 5.0 Hz, 2H), 7.14 (t, J = 8.3 Hz, 2H), 4.19 (dd, J = 9.5, 4.8 Hz, 1H), 2.92 – 2.75 (m, 1H), 2.62 – 2.44 (m, 1H).

^{19}F NMR (376 MHz, $CDCl_3$) δ −80.9 – −81.0 (m, 3F), −111.5 – −112.5 (m, 1F), −112.7 – −114.7 (m, 2F), −124.2 – −124.3 (m, 2F), −125.8 – −125.9 (m, 2F).

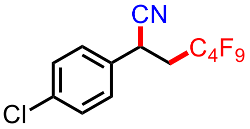

2-(4-Chlorophenyl)-4,4,5,5,6,6,7,7,7-nonafluoroheptanenitrile (5)[7]**.** The title compound was synthesized according to the General Procedure using 1-chloro-4-vinylbenzene (13.8 mg, 0.10 mmol) and C_4F_9I (103.8 mg, 0.30 mmol). The crude mixture was purified by flash column chromatography using hexanes/ethyl acetate (30:1 v/v) as the eluent to give 35.6 mg (0.093 mmol, 93% yield) of the title compound as a colorless oil.

^{1}H NMR (400 MHz, $CDCl_3$) δ 7.43 (d, J = 8.2 Hz, 2H), 7.34 (d, J = 8.2 Hz, 2H), 4.18 (dd, J = 9.5, 4.8 Hz, 1H), 2.93 – 2.75 (m, 1H), 2.60 – 2.45 (m, 1H).

^{19}F NMR (376 MHz, $CDCl_3$) δ −80.9 – −80.0 (m, 3F), −112.5 – −114.8 (m, 2F), −124.2 – −124.4 (m, 2F), −125. 8 – −125.9 (m, 2F).

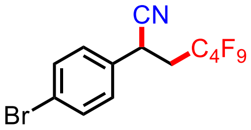

2-(4-Bromophenyl)-4,4,5,5,6,6,7,7,7-nonafluoroheptanenitrile (6)[7]**.** The title compound was synthesized according to the General Procedure using 1-bromo-4-vinylbenzene (18.2 mg, 0.10 mmol) and C_4F_9I (103.8 mg, 0.30 mmol). The crude mixture was purified by flash column chromatography using hexanes/ethyl acetate (30:1 v/v) as the eluent to give 37.1 mg (0.087 mmol, 87% yield) of the title compound as a colorless oil.

^{1}H NMR (400 MHz, $CDCl_3$) δ 7.63 – 7.57 (m, 2H), 7.32 – 7.29 (m, 2H), 4.19 (dd, J = 9.4, 4.8 Hz, 1H), 2.98 – 2.76 (m, 1H), 2.64 – 2.46 (m, 1H).

^{19}F NMR (376 MHz, $CDCl_3$) δ −81.0 – −80. 9 (m, 3F), −112.6 – −114.9 (m, 2F), −124.2 – −124.4 (m, 2F), −125.8 – −125.9 (m, 2F).

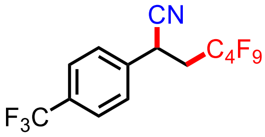

4,4,5,5,6,6,7,7,7-Nonafluoro-2-(4-(trifluoromethyl)phenyl)heptanenitrile (7)[7]**.** The title compound was synthesized according to the General Procedure using 1-(trifluoromethyl)-4-vinylbenzene (17.2 mg, 0.10 mmol) and C_4F_9I (103.8 mg, 0.30 mmol). The crude mixture was purified by flash column chromatography using hexanes/ethyl acetate (30:1 v/v) as the eluent to give 30.4 mg (0.073 mmol, 73% yield) of the title compound as a colorless oil.

^{1}H NMR (400 MHz, $CDCl_3$) δ 7.63 – 7.57 (m, 2H), 7.32 – 7.29 (m, 2H), 4.19 (dd, J = 9.4, 4.8 Hz, 1H), 2.98 – 2.76 (m, 1H), 2.64 – 2.46 (m, 1H).

^{19}F NMR (376 MHz, $CDCl_3$) δ −62.9 (s, 3F), −80.9 – −81.0 (m, 3F), −112.2 – −114.5 (m, 2F), −124.2– −124.3 (m, 2F), −125.8 – −125.9 (m, 2F).

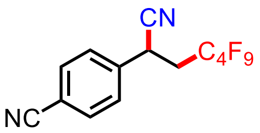

4-(1-Cyano-3,3,4,4,5,5,6,6,6-nonafluorohexyl)benzonitrile (8)[8]**.** The title compound was synthesized according to the General Procedure using 4-vinylbenzonitrile (12.9 mg, 0.10 mmol) and C_4F_9I (103.8 mg, 0.30 mmol). The crude mixture was purified by flash column chromatography using hexanes/ethyl acetate (10:1 v/v) as the eluent to give 25.4 mg (0.068 mmol, 68% yield) of the title compound as a colorless oil.

^{1}H NMR (400 MHz, $CDCl_3$) δ 7.77 (d, J = 8.4 Hz, 2H), 7.56 (d, J = 8.4 Hz, 2H), 4.28 (dd, J = 9.1, 5.1 Hz, 1H), 2.97 – 2.78 (m, 1H), 2.69 – 2.48 (m, 1H).

^{19}F NMR (376 MHz, $CDCl_3$) δ −80.8 – −81.1 (m, 3F), −112.3 – −114.6 (m, 2F), −124.0 – −124.6 (m, 2F), −125.6 – −126.3 (m, 2F).

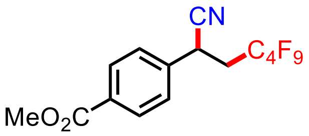

Methyl 4-(1-cyano-3,3,4,4,5,5,6,6,6-nonafluorohexyl)benzoate (9). The title compound was synthesized according to the General Procedure using methyl 4-vinylbenzoate (16.2 mg, 0.10 mmol) and C_4F_9I (103.8 mg, 0.30 mmol). The crude mixture was purified by flash column chromatography using hexanes/ethyl acetate (10:1 v/v) as the eluent to give 34.2 mg (0.084 mmol, 84% yield) of the title compound as a white solid.

^{1}H NMR (400 MHz, $CDCl_3$) δ 8.11 (d, J = 8.1 Hz, 2H), 7.49 (d, J = 8.1 Hz, 2H), 4.26 (dd, J = 9.5, 4.7 Hz, 1H), 3.94 (s, 3H), 2.96 – 2.78 (m, 1H), 2.66 – 2.48 (m, 1H).

^{13}C NMR (100 MHz, $CDCl_3$) δ 166.0, 138.3, 131.1, 130.9, 127.4, 118.1, 52.4, 36.7 (t, J = 21.3 Hz), 29.7.

^{19}F NMR (376 MHz, $CDCl_3$) δ −80.8 – −81.1 (m, 3F), −112.1 – −116.5 (m, 2F), −123.9 – −124.4 (m, 2F), −125.8 – −126.0 (m, 2F).

HRMS *m*/*z* (ESI) calcd. for $C_{15}H_{10}F_9NO_2Na$ $[M+Na]^+$: 430.0460; found: 430.0414.

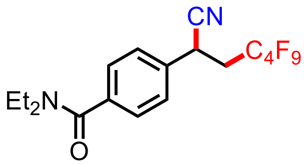

4-(1-Cyano-3,3,4,4,5,5,6,6,6-nonafluorohexyl)-*N*,*N*-diethylbenzamide (10). The title compound was synthesized according to the General Procedure using *N*,*N*-diethyl-4-vinylbenzamide (20.3 mg, 0.10 mmol) and C_4F_9I (103.8 mg, 0.30 mmol). The crude mixture was purified by flash column chromatography using hexanes/ethyl acetate (10:1 v/v) as the eluent to give 38.1 mg (0.085 mmol, 85% yield) of the title compound as a white solid.

^{1}H NMR (400 MHz, $CDCl_3$) δ 7.48 – 7.40 (m, 4H), 4.22 (dd, *J* = 9.7, 4.5 Hz, 1H), 3.56 (d, *J* = 8.6 Hz, 2H), 3.33 – 3.19 (m, 2H), 2.94 – 2.76 (m, 1H), 2.63 – 2.46 (m, 1H), 1.33 – 1.07 (m, 6H).

^{13}C NMR (100 MHz, $CDCl_3$) δ 170.0, 138.3, 134.6, 127.7, 127.4, 118.4, 43.3, 39.4, 36.9 (t, *J* = 21.6 Hz), 29.6, 14.2, 12.9.

^{19}F NMR (376 MHz, $CDCl_3$) δ −79.6 – −82.2 (m, 3F), −111.0 – −116.7 (m, 2F), −123.9 – −125.3 (m, 2F), −125.8 – −125.9 (m, 2F).

HRMS *m*/*z* (ESI) calcd. for $C_{18}H_{17}F_9N_2ONa$ $[M+Na]^+$: 471.1089; found: 471.1084.

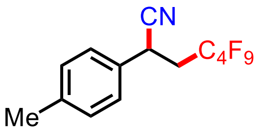

4,4,5,5,6,6,7,7,7-Nonafluoro-2-(*p*-tolyl)heptanenitrile (11)[7]**.** The title compound was synthesized according to the General Procedure using 1-methyl-4-vinylbenzene (11.8 mg, 0.10 mmol) and C_4F_9I (103.8 mg, 0.30 mmol). The crude mixture was purified by flash column chromatography using hexanes/ethyl acetate (30:1 v/v) as the eluent to give 34.9 mg (0.096 mmol, 96% yield) of the title compound as a colorless oil.

^{1}H NMR (400 MHz, $CDCl_3$) δ 7.29 – 7.21 (m, 4H), 4.16 (dd, *J* = 9.8, 4.5 Hz, 1H), 2.93 – 2.71 (m, 1H), 2.61 – 2.43 (m, 1H), 2.37 (s, 3H).

^{19}F NMR (376 MHz, $CDCl_3$) δ −80.7 – −81.2 (m, 3F), −110.9 – −116.0 (m, 2F), −122.8 – −125.5 (m, 2F), −125.5 – −126.0 (m, 2F).

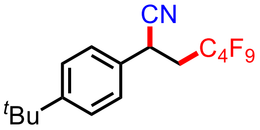

2-(4-(*Tert*-butyl)phenyl)-4,4,5,5,6,6,7,7,7-nonafluoroheptanenitrile (12)[8]. The title compound was synthesized according to the General Procedure using 1-(*tert*-butyl)-4-vinylbenzene (16.0 mg, 0.10 mmol) and C_4F_9I (103.8 mg, 0.30 mmol). The crude mixture was purified by flash column chromatography using hexanes/ethyl acetate (30:1 v/v) as the eluent to give 37.7 mg (0.093 mmol, 93% yield) of the title compound as a colorless oil.

^{1}H NMR (400 MHz, $CDCl_3$) δ 7.44 (d, *J* = 8.3 Hz, 2H), 7.31 (d, *J* = 8.4 Hz, 2H), 4.17 (dd, *J* = 10.1, 4.2 Hz, 1H), 2.93 – 2.70 (m, 1H), 2.65 – 2.44 (m, 1H), 1.35 (s, 9H).

^{19}F NMR (376 MHz, $CDCl_3$) δ −80.8 – −81.0 (m, 3F), −111.4 – −114.9 (m, 2F), −124.1 – −124.4 (m, 2F), −125.2 – −126.5 (m, 2F).

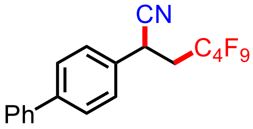

2-([1,1'-Biphenyl]-4-yl)-4,4,5,5,6,6,7,7,7-nonafluoroheptanenitrile (13)[7]. The title compound was synthesized according to the General Procedure using 4-vinyl-1,1'-biphenyl (18.0 mg, 0.10 mmol) and C_4F_9I (103.8 mg, 0.30 mmol). The crude mixture was purified by flash column chromatography using hexanes/ethyl acetate (30:1 v/v) as the eluent to give 37.8 mg (0.089 mmol, 89% yield) of the title compound as a colorless oil.

^{1}H NMR (400 MHz, $CDCl_3$) δ 7.65 (d, *J* = 8.0 Hz, 2H), 7.58 (d, *J* = 7.6 Hz, 2H), 7.49 – 7.41 (m, 4H), 7.39 (t, *J* = 7.4 Hz, 1H), 4.24 (dd, *J* = 9.9, 4.5 Hz, 1H), 2.96 – 2.80 (m, 1H), 2.68 – 2.50 (m, 1H).

^{19}F NMR (376 MHz, $CDCl_3$) δ −80.8 – −81.1 (m, 3F), −112.3 – −116.7 (m, 2F), −124.1 – −124.4 (m, 2F), −125.7 – −125.9 (m, 2F).

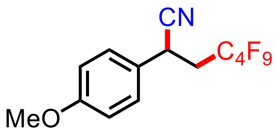

4,4,5,5,6,6,7,7,7-Nonafluoro-2-(4-methoxyphenyl)heptanenitrile (14)[7]**.** The title compound was synthesized according to the General Procedure using 1-methoxy-4-vinylbenzene (13.4 mg, 0.10 mmol) and C_4F_9I (103.8 mg, 0.30 mmol). The crude mixture was purified by flash column chromatography using hexanes/ethyl acetate (30:1 v/v) as the eluent to give 30.7 mg (0.081 mmol, 81% yield) of the title compound as a colorless oil.

^{1}H NMR (400 MHz, $CDCl_3$) δ 7.30 (d, J = 8.6 Hz, 2H), 6.94 (d, J = 8.5 Hz, 2H), 4.15 (dd, J = 9.7, 4.6 Hz, 1H), 3.83 (s, 3H), 2.91 – 2.72 (m, 1H), 2.60 – 2.42 (m, 1H).

^{19}F NMR (376 MHz, $CDCl_3$) δ −80.8 – −81.1 (m, 3F), −112.4 – −115.5 (m, 2F), −124.2 – −124.4 (m, 2F), −125.4 – −126.5 (m, 2F).

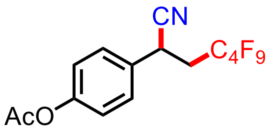

4-(1-Cyano-3,3,4,4,5,5,6,6,6-nonafluorohexyl)phenyl acetate (15)[7]**.** The title compound was synthesized according to the General Procedure using 4-vinylphenyl acetate (16.2 mg, 0.10 mmol) and C_4F_9I (103.8 mg, 0.30 mmol). The crude mixture was purified by flash column chromatography using hexanes/ethyl acetate (10:1 v/v) as the eluent to give 37.9 mg (0.093 mmol, 93% yield) of the title compound as a colorless oil.

^{1}H NMR (400 MHz, $CDCl_3$) δ 7.44 – 7.39 (m, 2H), 7.21 – 7.15 (m, 2H), 4.20 (dd, J = 9.9, 4.4 Hz, 1H), 2.93 – 2.75 (m, 1H), 2.67 – 2.46 (m, 1H), 2.32 (s, 3H).

^{19}F NMR (376 MHz, $CDCl_3$) δ −80.8 – −81.0 (m, 3F), −111.8 – −116.2 (m, 2F), −124.2 – −124.4 (m, 2F), −125.0 – −126.3 (m, 2F).

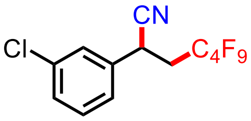

2-(3-Chlorophenyl)-4,4,5,5,6,6,7,7,7-nonafluoroheptanenitrile (16)[7]**.** The title compound was synthesized according to the General Procedure using 1-chloro-3-vinylbenzene (13.8 mg, 0.10 mmol) and C_4F_9I (103.8 mg, 0.30 mmol). The crude mixture was purified by flash column chromatography using hexanes/ethyl acetate (30:1 v/v) as the eluent to give 32.6 mg (0.085 mmol, 85% yield) of the title compound as a colorless oil.

^{1}H NMR (400 MHz, $CDCl_3$) δ 7.42 – 7.35 (m, 3H), 7.33 – 7.27 (m, 1H), 4.18 (dd, J = 9.6, 4.6 Hz, 1H), 2.95 – 2.76 (m, 1H), 2.66 – 2.45 (m, 1H).

^{19}F NMR (376 MHz, $CDCl_3$) δ −80.9 – −81.0 (m, 3F), −112.4 – −115.5 (m, 2F), −123.8 – −124.8 (m, 2F), −125.7 – −126.2 (m, 2F).

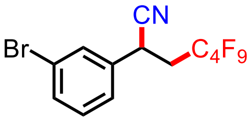

2-(3-Bromophenyl)-4,4,5,5,6,6,7,7,7-nonafluoroheptanenitrile (17)[7]**.** The title compound was synthesized according to the General Procedure using 1-bromo-3-vinylbenzene (18.2 mg, 0.10 mmol) and C_4F_9I (103.8 mg, 0.30 mmol). The crude mixture was purified by flash column chromatography using hexanes/ethyl acetate (30:1 v/v) as the eluent to give 37.1 mg (0.087 mmol, 87% yield) of the title compound as a colorless oil.

^{1}H NMR (400 MHz, $CDCl_3$) δ 7.56 – 7.51 (m, 2H), 7.37 – 7.29 (m, 2H), 4.16 (dd, J = 9.7, 4.6 Hz, 1H), 2.94 – 2.74 (m, 1H), 2.66 – 2.43 (m, 1H).

^{19}F NMR (376 MHz, $CDCl_3$) δ −79.8 – −81.6 (m, 3F), −112.0 – −116.2 (m, 2F), −124.1 – −124.3 (m, 2F), −125.7 – −125.9 (m, 2F).

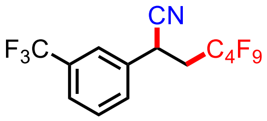

4,4,5,5,6,6,7,7,7-Nonafluoro-2-(3-(trifluoromethyl)phenyl)heptanenitrile (18). The title compound was synthesized according to the General Procedure using 1-(trifluoromethyl)-3-vinylbenzene (17.2 mg, 0.10 mmol) and C_4F_9I (103.8 mg, 0.30 mmol). The crude mixture was purified by flash column chromatography using hexanes/ethyl acetate (30:1 v/v) as the eluent to give 23.8 mg (0.057 mmol, 57% yield) of the title compound as a colorless oil.

^{1}H NMR (400 MHz, $CDCl_3$) δ 7.74 – 7.54 (m, 4H), 4.28 (dd, J = 9.8, 4.8 Hz, 1H), 2.96 – 2.81 (m, 1H), 2.64 – 2.50 (m, 1H).

^{13}C NMR (100 MHz, $CDCl_3$) δ 134.8, 132.3 (q, J = 32.9 Hz), 130.7, 130.4, 126.2 (q, J = 3.7 Hz), 124.2 (q, J = 3.8 Hz), 123.4 (q, J = 271.0 Hz), 118.0, 36.8 (t, J = 21.4 Hz), 29.7 (t, J = 3.9 Hz).

^{19}F NMR (376 MHz, $CDCl_3$) δ −62.8 (s, 3F), −80.8 – −81.2 (m, 3F), −113.2 – −113.9 (m, 2F), −124.21– −124.3 (m, 2F), −125.6 – −126.3 (m, 2F).

HRMS *m/z* (ESI) calcd. for $C_{14}H_7F_{12}NNa$ $[M+Na]^+$: 440.0279; found: 440.0278.

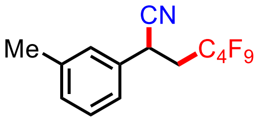

4,4,5,5,6,6,7,7,7-Nonafluoro-2-(*m*-tolyl)heptanenitrile (19)[7]**.** The title compound was synthesized according to the General Procedure using 1-methyl-3-vinylbenzene (11.8 mg, 0.10 mmol) and C_4F_9I (103.8 mg, 0.30 mmol). The crude mixture was purified by flash column chromatography using hexanes/ethyl acetate (30:1 v/v) as the eluent to give 31.9 mg (0.088 mmol, 88% yield) of the title compound as a colorless oil.

^{1}H NMR (400 MHz, $CDCl_3$) δ 7.32 (t, J = 7.8 Hz, 1H), 7.23 – 7.13 (m, 3H), 4.15 (dd, J = 10.0, 4.3 Hz, 1H), 2.93 – 2.75 (m, 1H), 2.66 – 2.44 (m, 1H), 2.39 (s, 3H).

^{19}F NMR (376 MHz, $CDCl_3$) δ −80. 9 – −81.0 (m, 3F), −111.9 – −116.2 (m, 2F), −124.2 – −124.4 (m, 2F), −125.0 – −127.5 (m, 2F).

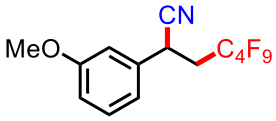

4,4,5,5,6,6,7,7,7-Nonafluoro-2-(3-methoxyphenyl)heptanenitrile (20)[8]. The title compound was synthesized according to the General Procedure using 1-methoxy-3-vinylbenzene (13.4 mg, 0.10 mmol) and C_4F_9I (103.8 mg, 0.30 mmol). The crude mixture was purified by flash column chromatography using hexanes/ethyl acetate (20:1 v/v) as the eluent to give 32.2 mg (0.085 mmol, 85% yield) of the title compound as a colorless oil.

^{1}H NMR (400 MHz, $CDCl_3$) δ 7.39 – 7.31 (m, 1H), 7.04 – 6.89 (m, 3H), 4.15 (dd, J = 10.0, 4.3 Hz, 1H), 3.84 (s, 3H), 2.93 – 2.75 (m, 1H), 2.63 – 2.44 (m, 1H).

^{19}F NMR (376 MHz, $CDCl_3$) δ −80.9 – −80.0 (m, 3F), −112.0 – −115.3 (m, 2F), −124.2 – −124.4 (m, 2F), −125.8 – −125.9 (m, 2F).

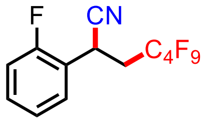

4,4,5,5,6,6,7,7,7-Nonafluoro-2-(2-fluorophenyl)heptanenitrile (21)[8]. The title compound was synthesized according to the General Procedure using 1-fluoro-2-vinylbenzene (12.2 mg, 0.10 mmol) and C_4F_9I (103.8 mg, 0.30 mmol). The crude mixture was purified by flash column chromatography using hexanes/ethyl acetate (30:1 v/v) as the eluent to give 27.2 mg (0.074 mmol, 74% yield) of the title compound as a colorless oil.

^{1}H NMR (400 MHz, $CDCl_3$) δ 7.54 – 7.50 (m, 1H), 7.46 – 7.39 (m, 1H), 7.28 – 7.21 (m, 1H), 7.16 (dd, J = 10.1, 8.5 Hz, 1H), 4.48 (dd, J = 9.4, 4.7 Hz, 1H), 2.94 – 2.75 (m, 1H), 2.72 – 2.50 (m, 1H).

^{19}F NMR (376 MHz, $CDCl_3$) δ −80.9 – −81.0 (m, 3F), −112.1 – −115.9 (m, 2F), −117.5 (s, 1F), −124.2 – −124.4 (m, 2F), −125.0 – −126.4 (m, 2F).

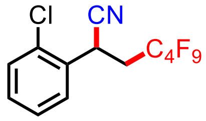

2-(2-Chlorophenyl)-4,4,5,5,6,6,7,7,7-nonafluoroheptanenitrile (22)[7]. The title compound was synthesized according to the General Procedure using 1-chloro-2-vinylbenzene (13.9 mg, 0.10 mmol) and C_4F_9I (103.8 mg, 0.30 mmol). The crude mixture was purified by flash column chromatography using hexanes/ethyl acetate (30:1 v/v) as the eluent to give 28.3 mg (0.074 mmol, 74% yield) of the title compound as a colorless oil.

^{1}H NMR (400 MHz, $CDCl_3$) δ 7.68 – 7.60 (m, 1H), 7.51 – 7.44 (m, 1H), 7.43 – 7.34 (m, 2H), 4.69 (dd, J = 10.2, 3.8 Hz, 1H), 2.85 – 2.51 (m, 2H).

^{19}F NMR (376 MHz, $CDCl_3$) δ −80.9 – −81.1 (m, 3F), −111.9 – −116.3 (m, 2F), −124.2 – −124.4 (m, 2F), −125.7 – −125.9 (m, 2F).

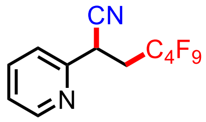

4,4,5,5,6,6,7,7,7-Nonafluoro-2-(pyridin-2-yl)heptanenitrile (23)[8]. The title compound was synthesized according to the General Procedure using 2-vinylpyridine (10.5 mg, 0.10 mmol) and C_4F_9I (103.8 mg, 0.30 mmol). The crude mixture was purified by flash column chromatography using hexanes/ethyl acetate (10:1 v/v) as the eluent to give 28.4 mg (0.081 mmol, 81% yield) of the title compound as a colorless oil.

^{1}H NMR (400 MHz, $CDCl_3$) δ 8.64 (d, J = 4.8 Hz, 1H), 7.86 – 7.75 (m, 1H), 7.54 (d, J = 7.8 Hz, 1H), 7.43 – 7.30 (m, 1H), 4.36 (dd, J = 9.3, 4.7 Hz, 1H), 3.16 – 2.98 (m, 1H), 2.97 – 2.79 (m, 1H).

^{19}F NMR (376 MHz, $CDCl_3$) δ −81.0 (dd, J = 11.5, 7.6 Hz, 3F), −111.1 – −115.8 (m, 2F), −124.3 (ABq, J = 9.3, 8.7 Hz, 2F), −125.9 (t, J = 11.5 Hz, 2F).

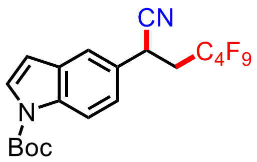

***Tert*-butyl 5-(1-cyano-3,3,4,4,5,5,6,6,6-nonafluorohexyl)-1*H*-indole-1-carboxylate (24).** The title compound was synthesized according to the General Procedure using *tert*-butyl 5-vinyl-1*H*-indole-1-carboxylate (24.3 mg, 0.10 mmol) and C_4F_9I (103.8 mg, 0.30 mmol). The crude mixture was purified by flash column chromatography using hexanes/ethyl acetate (30:1 v/v) as the eluent to give 39.5 mg (0.081 mmol, 81% yield) of the title compound as a white solid.

^{1}H NMR (400 MHz, $CDCl_3$) δ 8.20 (d, *J* = 8.6 Hz, 1H), 7.66 (d, *J* = 3.7 Hz, 1H), 7.61 (s, 1H), 7.36 – 7.25 (m, 1H), 6.59 (d, *J* = 3.7 Hz, 1H), 4.29 (dd, *J* = 10.0, 4.5 Hz, 1H), 2.98 – 2.79 (m, 1H), 2.70 – 2.45 (m, 1H), 1.68 (s, 9H).

^{13}C NMR (100 MHz, $CDCl_3$) δ 149.4, 135.1, 131.3, 128.1, 127.4, 122.9, 119.7, 119.2, 116.3, 106.9, 84.3, 37.4 (t, *J* = 21.2 Hz), 29.7, 28.1.

^{19}F NMR (376 MHz, $CDCl_3$) δ −80.9 – −81.0 (m, 3F), −112.3 – −115.5 (m, 2F), −124.2 – −124.4 (m, 2F), −125.7 – −126.0 (m, 2F).

HRMS *m*/*z* (ESI) calcd. for $C_{20}H_{17}F_9N_2O_2Na$ $[M+Na]^+$: 511.1039; found: 511.1024.

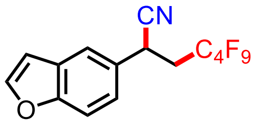

2-(Benzofuran-5-yl)-4,4,5,5,6,6,7,7,7-nonafluoroheptanenitrile (25). The title compound was synthesized according to the General Procedure using 5-vinylbenzofuran (14.4 mg, 0.10 mmol) and C_4F_9I (103.8 mg, 0.30 mmol). The crude mixture was purified by flash column chromatography using hexanes/ethyl acetate (30:1 v/v) as the eluent to give 32.3 mg (0.083 mmol, 83% yield) of the title compound as a colorless oil.

^{1}H NMR (400 MHz, $CDCl_3$) δ 7.70 (d, *J* = 2.1 Hz, 1H), 7.66 (d, *J* = 2.1 Hz, 1H), 7.56 (d, *J* = 8.6 Hz, 1H), 7.28 (dd, *J* = 8.5, 2.1 Hz, 1H), 6.88 – 6.73 (m, 1H), 4.30 (dd, *J* = 9.8, 4.6 Hz, 1H), 3.00 – 2.79 (m, 1H), 2.69 – 2.49 (m, 1H).

^{13}**C NMR** (100 MHz, $CDCl_3$) δ 154.8, 146.6, 128.52, 128.47, 123.2, 120.2, 119.1, 112.6, 106.5, 37.5 (t, J = 21.0 Hz), 29.7.

^{19}**F NMR** (376 MHz, $CDCl_3$) δ −80.8 – −81.0 (m, 3F), −112.7 – −115.8 (m, 2F), −124.2 – −124.4 (m, 2F), −125.6 – −126.2 (m, 2F).

HRMS m/z (ESI) calcd. for $C_{15}H_8F_9NONa$ $[M+Na]^+$: 412.0354; found: 412.0374.

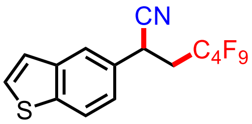

2-(Benzo[*b*]thiophen-5-yl)-4,4,5,5,6,6,7,7,7-nonafluoroheptanenitrile (26). The title compound was synthesized according to the General Procedure using 5-vinylbenzo[*b*]thiophene (16.0 mg, 0.10 mmol) and C_4F_9I (103.8 mg, 0.30 mmol). The crude mixture was purified by flash column chromatography using hexanes/ethyl acetate (30:1 v/v) as the eluent to give 37.3 mg (0.092 mmol, 92% yield) of the title compound as a colorless oil.

^{1}**H NMR** (400 MHz, $CDCl_3$) δ 7.94 (d, J = 8.4 Hz, 1H), 7.88 (d, J = 1.9 Hz, 1H), 7.56 (d, J = 5.5 Hz, 1H), 7.39 – 7.29 (m, 2H), 4.33 (dd, J = 9.8, 4.5 Hz, 1H), 3.02 – 2.81 (m, 1H), 2.70 – 2.50 (m, 1H).

^{13}**C NMR** (100 MHz, $CDCl_3$) δ 140.3, 129.9, 128.5, 123.9, 123.6, 122.8, 122.3, 118.9, 37.4 (t, J = 21.3 Hz), 29.8.

^{19}**F NMR** (376 MHz, $CDCl_3$) δ −80.9 – −81.1 (m, 3F), −112.2 – −115.5 (m, 2F), −124.2 – −124.4 (m, 2F), −125.7 – −126.0 (m, 2F).

HRMS m/z (ESI) calcd. for $C_{15}H_8F_9NSNa$ $[M+Na]^+$: 428.0126; found: 428.0138.

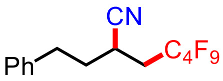

4,4,5,5,6,6,7,7,7-Nonafluoro-2-phenethylheptanenitrile (27)[8]. The title compound was synthesized according to the General Procedure using but-3-en-1-ylbenzene (13.2 mg, 0.10 mmol) and C_4F_9I (103.8 mg, 0.30 mmol). The crude mixture was purified by flash column chromatography using hexanes/ethyl acetate (30:1 v/v) as the eluent to give 26.8 mg (0.071

mmol, 71% yield) of the title compound as a colorless oil.

^{1}H NMR (400 MHz, $CDCl_3$) δ 7.36 – 7.30 (m, 2H), 7.28 – 7.24 (m, 1H), 7.24 – 7.18 (m, 2H), 3.02 – 2.89 (m, 2H), 2.86 – 2.76 (m, 1H), 2.63 – 2.47 (m, 1H), 2.40 – 2.19 (m, 1H), 2.16 – 1.98 (m, 2H).

^{19}F NMR (376 MHz, $CDCl_3$) δ −80.9 – −81.1 (m, 3F), −112.1 – −115.1 (m, 2F), −124.3 – −124.8 (m, 2F), −125.8 – −126.0 (m, 2F).

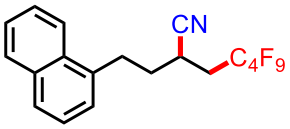

4,4,5,5,6,6,7,7,7-Nonafluoro-2-(2-(naphthalen-1-yl)ethyl)heptanenitrile (28). The title compound was synthesized according to the General Procedure using 1-(but-3-en-1-yl)naphthalene (18.2 mg, 0.10 mmol) and C_4F_9I (103.8 mg, 0.30 mmol). The crude mixture was purified by flash column chromatography using hexanes/ethyl acetate (30:1 v/v) as the eluent to give 31.6 mg (0.074 mmol, 74% yield) of the title compound as a colorless oil.

^{1}H NMR (400 MHz, $CDCl_3$) δ 8.00 (d, J = 8.4 Hz, 1H), 7.89 (d, J = 8.0 Hz, 1H), 7.78 (d, J = 8.1 Hz, 1H), 7.59 – 7.50 (m, 2H), 7.47 – 7.33 (m, 2H), 3.47 (td, J = 9.1, 4.6 Hz, 1H), 3.29 – 3.19 (m, 1H), 3.06 – 2.96 (m, 1H), 2.70 – 2.50 (m, 1H), 2.42 – 2.09 (m, 3H).

^{13}C NMR (100 MHz, $CDCl_3$) δ 135.1, 134.0, 131.4, 129.1, 127.7, 126.5, 126.4, 125.9, 125.6, 123.0, 119.7, 33.6, 33.5 (t, J = 22.4) 30.2, 24.0.

^{19}F NMR (376 MHz, $CDCl_3$) δ −80.9 – −81.0 (m, 3F), −112.6 – −115.0 (m, 2F), −124.3 – −124.5 (m, 2F), −125.8 – −126.2 (m, 2F).

HRMS *m/z* (ESI) calcd. for $C_{19}H_{14}F_9NNa$ $[M+Na]^+$: 450.0875; found: 450.0871.

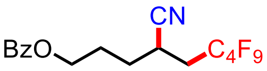

4-Cyano-6,6,7,7,8,8,9,9,9-nonafluorononyl benzoate (29). The title compound was synthesized according to the General Procedure using pent-4-en-1-yl benzoate (23.1 mg, 0.10

mmol) and C_4F_9I (103.8 mg, 0.30 mmol). The crude mixture was purified by flash column chromatography using hexanes/ethyl acetate (30:1 v/v) as the eluent to give 23.1 mg (0.053 mmol, 53% yield) of the title compound as a colorless oil.

^{1}H NMR (400 MHz, $CDCl_3$) δ 8.04 (d, *J* = 7.8 Hz, 2H), 7.59 (t, *J* = 7.4 Hz, 1H), 7.46 (t, *J* = 7.7 Hz, 2H), 4.44 – 4.36 (m, 2H), 3.15 – 3.06 (m, 1H), 2.68 – 2.49 (m, 1H), 2.46 – 2.24 (m, 1H), 2.22 – 2.07 (m, 1H), 2.07 – 1.85 (m, 3H).

^{13}C NMR (100 MHz, $CDCl_3$) δ 166.4, 133.2, 129.8, 129.5, 128.4, 119.4, 63.4, 33.5 (t, *J* = 21.8 Hz), 29.6, 26.2, 24.0.

^{19}F NMR (376 MHz, $CDCl_3$) δ −80.9 – −81.1 (m, 3F), −112.5 – −114.6 (m, 2F), −124.2 – −124.5 (m, 2F), −125.8 – −126.0 (m, 2F).

HRMS *m*/*z* (ESI) calcd. for $C_{17}H_{14}F_9NO_2Na$ $[M+Na]^+$: 458.0773; found: 458.0793.

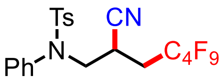

***N*-(2-Cyano-4,4,5,5,6,6,7,7,7-nonafluoroheptyl)-4-methyl-*N*-phenylbenzenesulfonamide (30).** The title compound was synthesized according to the General Procedure using *N*-allyl-4-methyl-*N*-phenylbenzenesulfonamide (28.7 mg, 0.10 mmol) and C_4F_9I (103.8 mg, 0.30 mmol). The crude mixture was purified by flash column chromatography using hexanes/ethyl acetate (5:1 v/v) as the eluent to give 34.6 mg (0.065 mmol, 65% yield) of the title compound as a colorless oil.

^{1}H NMR (400 MHz, $CDCl_3$) δ 7.48 – 7.41 (m, 2H), 7.32 – 7.41 (m, 3H), 7.28 (d, *J* = 8.3 Hz, 2H), 7.06 – 6.98 (m, 2H), 3.94 (dd, *J* = 13.8, 9.1 Hz, 1H), 3.86 – 3.72 (m, 1H), 3.15 – 3.04 (m, 1H), 2.84 – 2.66 (m, 1H), 2.65 – 2.48 (m, 1H), 2.45 (s, 3H).

^{13}C NMR (100 MHz, $CDCl_3$) δ 144.5, 138.1, 134.0, 129.8, 129.7, 129.1, 128.7, 127.8, 117.8, 51.8, 30.8 (t, *J* = 21.9 Hz), 24.1, 21.6.

^{19}F NMR (376 MHz, $CDCl_3$) δ −80.9 (t, *J* = 9.6 Hz, 3F), −111.8 – −114.8 (m, 2F), −124.1 – −124.3 (m, 2F), −125.2 – −126.3 (m, 2F).

HRMS *m*/*z* (ESI) calcd. for $C_{21}H_{17}F_9N_2O_2SNa$ $[M+Na]^+$: 555.0759; found: 555.0747.

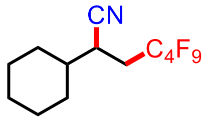

2-Cyclohexyl-4,4,5,5,6,6,7,7,7-nonafluoroheptanenitrile (31)[8]. The title compound was synthesized according to the General Procedure using vinylcyclohexane (11.0 mg, 0.10 mmol) and C_4F_9I (103.8 mg, 0.30 mmol). The crude mixture was purified by flash column chromatography using hexanes/ethyl acetate (30:1 v/v) as the eluent to give 25.2 mg (0.071 mmol, 71% yield) of the title compound as a colorless oil.

^{1}H NMR (400 MHz, $CDCl_3$) δ 2.86 (td, J = 9.1, 4.3 Hz, 1H), 2.60 – 2.43 (m, 1H), 2.38 – 2.18 (m, 1H), 2.02 – 1.66 (m, 5H), 1.67 – 1.60 (m, 1H), 1.38 – 1.13 (m, 5H).

^{19}F NMR (376 MHz, $CDCl_3$) δ −80.9 – −81.1 (m, 3F), −112.5 – −116.0 (m, 2F), −124.2 – −124.4 (m, 2F), −125.87 – −126.0 (m, 2F).

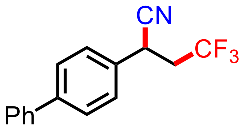

2-([1,1'-Biphenyl]-4-yl)-4,4,4-trifluorobutanenitrile (32)[9]. The title compound was synthesized according to the General Procedure using 4-vinyl-1,1'-biphenyl (18.0 mg, 0.10 mmol) and CF_3I in a DMF solution (1.0 M) (103.8 mg, 0.30 mL, 0.30 mmol). The crude mixture was purified by flash column chromatography using hexanes/ethyl acetate (30:1 v/v) as the eluent to give 21.2 mg (0.077 mmol, 77% yield) of the title compound as a colorless oil.

^{1}H NMR (400 MHz, $CDCl_3$) δ 7.68 – 7.62 (m, 2H), 7.61 – 7.55 (m, 2H), 7.50 – 7.43 (m, 4H), 7.42 – 7.33 (m, 1H), 4.15 (dd, J = 9.6, 5.1 Hz, 1H), 2.96 – 2.79 (m, 1H), 2.72 – 2.56 (m, 1H).

^{19}F NMR (376 MHz, $CDCl_3$) δ −65.0.

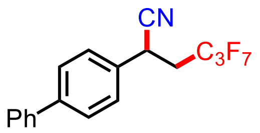

2-([1,1'-Biphenyl]-4-yl)-4,4,5,5,6,6,6-heptafluorohexanenitrile (33). The title compound was synthesized according to the General Procedure using 4-vinyl-1,1'-biphenyl (18.0 mg, 0.10 mmol) and C_3F_7I (88.8 mg, 0.30 mmol). The crude mixture was purified by flash column chromatography using hexanes/ethyl acetate (30:1 v/v) as the eluent to give 34.1 mg (0.091 mmol, 91% yield) of the title compound as a white solid.

^{1}H NMR (400 MHz, $CDCl_3$) δ 7.65 (d, *J* = 7.9 Hz, 2H), 7.58 (d, *J* = 6.9 Hz, 2H), 7.50 – 7.40 (m, 4H), 7.39 (t, *J* = 7.3 Hz, 1H), 4.24 (dd, *J* = 9.8, 4.5 Hz, 1H), 2.97 – 2.77 (m, 1H), 2.67 – 2.48 (m, 1H).

^{13}C NMR (100 MHz, $CDCl_3$) δ 142.2, 139.8, 132.6, 128.9, 128.3, 127.9, 127.6, 127.1, 118.7, 36.8 (t, *J* = 21.3 Hz), 29.4.

^{19}F NMR (376 MHz, $CDCl_3$) δ −80.3 (t, *J* = 9.6 Hz, 3F), −112.6 – −118.6 (m, 2F), −127.6 (s, 2F).

HRMS *m/z* (ESI) calcd. for $C_{18}H_{12}F_7NNa$ $[M+Na]^+$: 398.0750; found: 398.0761.

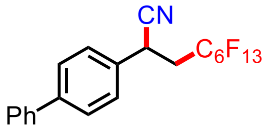

2-([1,1'-Biphenyl]-4-yl)-4,4,5,5,6,6,7,7,8,8,9,9,9-tridecafluorononanenitrile (34). The title compound was synthesized according to the General Procedure using 4-vinyl-1,1'-biphenyl (18.0 mg, 0.10 mmol) and $C_6F_{13}I$ (133.8 mg, 0.30 mmol). The crude mixture was purified by flash column chromatography using hexanes/ethyl acetate (30:1 v/v) as the eluent to give 46.7 mg (0.089 mmol, 89% yield) of the title compound as a white solid.

^{1}H NMR (400 MHz, $CDCl_3$) δ 7.68 – 7.61 (m, 2H), 7.60 – 7.55 (m, 2H), 7.50 – 7.45 (m, 4H), 7.43 – 7.36 (m, 1H), 4.24 (dd, *J* = 9.8, 4.5 Hz, 1H), 3.06 – 2.81 (m, 1H), 2.68 – 2.50 (m, 1H).

^{13}C NMR (100 MHz, $CDCl_3$) δ 142.2, 139.8, 132.6, 128.9, 128.3, 127.9, 127.6, 127.1, 118.7,

37.1 (t, J = 21.8 Hz), 29.5.

^{19}F NMR (376 MHz, $CDCl_3$) δ −80.6 – −80.8 (m, 3F), −112.4 – −114.7 (m, 2F), −121.5 – −121.8 (m, 2F), −122.4 – −122.9 (m, 2F), −123.2 – −123.5 (m, 2F), −125.4 – −126.6 (m, 2F).

HRMS *m*/*z* (ESI) calcd. for $C_{21}H_{12}F_{13}NNa$ $[M+Na]^+$: 548.0654; found: 548.0647.

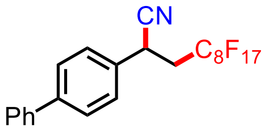

2-([1,1'-Biphenyl]-4-yl)-4,4,5,5,6,6,7,7,8,8,9,9,10,10,11,11,11-heptadecafluoroundecane-nitrile (35). The title compound was synthesized according to the General Procedure using 4-vinyl-1,1'-biphenyl (18.0 mg, 0.10 mmol) and $C_8F_{17}I$ (163.8 mg, 0.30 mmol). The crude mixture was purified by flash column chromatography using hexanes/ethyl acetate (30:1 v/v) as the eluent to give 53.8 mg (0.086 mmol, 86% yield) of the title compound as a white solid.

^{1}H NMR (400 MHz, $CDCl_3$) δ 7.71 – 7.63 (m, 2H), 7.61 – 7.56 (m, 2H), 7.51 – 7.45 (m, 4H), 7.42 – 7.34 (m, 1H), 4.24 (dd, J = 9.8, 4.5 Hz, 1H), 3.00 – 2.78 (m, 1H), 2.69 – 2.49 (m, 1H).

^{13}C NMR (100 MHz, $CDCl_3$) δ 142.2, 139.8, 132.6, 128.9, 128.3, 127.9, 127.6, 127.1, 118.7, 37.1 (t, J = 21.4 Hz), 29.5.

^{19}F NMR (376 MHz, $CDCl_3$) δ −80.7 (t, J = 9.9 Hz, 3F), −112.3 – −114.5 (m, 2F), −121.3 – −121.6 (m, 2F), −121.7 – −121.9 (m, 4F), −122.4 – −122.9 (m, 2F), −123.1 – −123.4 (m, 2F), −126.0 – −125.2 (m, 2F).

HRMS *m*/*z* (ESI) calcd. for $C_{23}H_{12}F_{17}NNa$ $[M+Na]^+$: 648.0590; found: 648.0607.

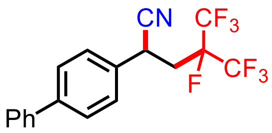

2-([1,1'-Biphenyl]-4-yl)-4,5,5,5-tetrafluoro-4-(trifluoromethyl)pentanenitrile (36). The title compound was synthesized according to the General Procedure using 4-vinyl-1,1'-biphenyl (18.0 mg, 0.10 mmol) and $(CF_3)_2CFI$ (88.8 mg, 0.30 mmol). The crude mixture was purified by flash column chromatography using hexanes/ethyl acetate (30:1 v/v) as the eluent to give 30.4 mg (0.081 mmol, 81% yield) of the title compound as a colorless oil.

^{1}H NMR (400 MHz, $CDCl_3$) δ 7.68 – 7.62 (m, 2H), 7.61 – 7.55 (m, 2H), 7.49 – 7.43 (m, 4H), 7.42 – 7.36 (m, 1H), 4.19 (dd, J = 10.6, 3.6 Hz, 1H), 2.98 – 2.82 (m, 1H), 2.61 – 2.44 (m, 1H).

^{13}C NMR (100 MHz, $CDCl_3$) δ 142.2, 139.8, 133.2, 128.9, 128.4, 127.9, 127.5, 127.1, 118.7, 35.0 (d, J = 19.5 Hz), 30.8.

^{19}F NMR (376 MHz, $CDCl_3$) δ −75.9 – −76.1 (m, 3F), −76.9 – −77.1 (m, 3F), −185.2 – −185.4 (m, 1F).

HRMS m/z (ESI) calcd. for $C_{18}H_{12}F_7NNa$ $[M+Na]^+$: 398.0750; found: 398.0741.

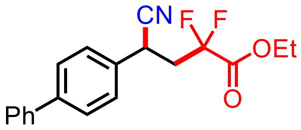

Ethyl 4-([1,1'-biphenyl]-4-yl)-4-cyano-2,2-difluorobutanoate (37)[10]**.** The title compound was synthesized according to the General Procedure using 4-vinyl-1,1'-biphenyl (18.0 mg, 0.10 mmol) and ethyl iododifluoroacetate (75.0 mg, 0.30 mmol). The crude mixture was purified by flash column chromatography using hexanes/ethyl acetate (10:1 v/v) as the eluent to give 30.3 mg (0.092 mmol, 92% yield) of the title compound as a colorless oil.

^{1}H NMR (400 MHz, $CDCl_3$) δ 7.66 – 7.60 (m, 2H), 7.60 – 7.55 (m, 2H), 7.49 – 7.41 (m, 4H), 7.41 – 7.34 (m, 1H), 4.35 – 4.22 (m, 2H), 4.17 (dd, J = 9.3, 5.1 Hz, 1H), 2.96 – 2.79 (m, 1H), 2.73 – 2.49 (m, 1H), 1.34 (t, J = 7.1 Hz, 3H).

^{19}F NMR (376 MHz, $CDCl_3$) δ −102.5 – −109.6 (m).

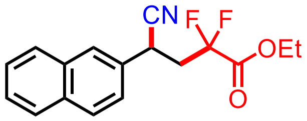

Ethyl 4-cyano-2,2-difluoro-4-(naphthalen-2-yl)butanoate (38). The title compound was synthesized according to the General Procedure using 4-vinyl-1,1'-biphenyl (15.4 mg, 0.10 mmol) and ethyl iododifluoroacetate (75.0 mg, 0.30 mmol). The crude mixture was purified by flash column chromatography using hexanes/ethyl acetate (10:1 v/v) as the eluent to give 26.1 mg (0.086 mmol, 86% yield) of the title compound as a white solid.

^{1}H NMR (400 MHz, $CDCl_3$) δ 7.94 – 7.80 (m, 4H), 7.61 – 7.50 (m, 2H), 7.43 (dd, J = 8.6, 1.9 Hz, 1H), 4.29 (dd, J = 9.3, 5.1 Hz, 1H), 4.26 – 4.17 (m, 2H), 2.98 – 2.82 (m, 1H), 2.78 – 2.61 (m, 1H), 1.30 (t, J = 7.2 Hz, 3H).

^{13}C NMR (100 MHz, $CDCl_3$) δ 162.9, 133.2, 133.0, 131.2, 129.6, 127.9, 127.8, 127.04, 126.98, 126.7, 124.4, 119.2, 113.9 (t, J = 252.0 Hz), 63.5, 40.1 (t, J = 23.9 Hz), 30.9 (t, J = 4.7 Hz), 13.8.

^{19}F NMR (376 MHz, $CDCl_3$) δ −104.8 – −105.3 (m).

HRMS m/z (ESI) calcd. for $C_{17}H_{15}F_2NO_2Na$ $[M+Na]^+$: 326.0963; found: 326.0982.

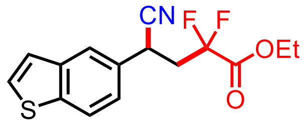

Ethyl 4-(benzo[*b*]thiophen-5-yl)-4-cyano-2,2-difluorobutanoate (39). The title compound was synthesized according to the General Procedure using 5-vinylbenzo[*b*]thiophene (16.0 mg, 0.10 mmol) and ethyl iododifluoroacetate (75.0 mg, 0.30 mmol). The crude mixture was purified by flash column chromatography using hexanes/ethyl acetate (10:1 v/v) as the eluent to give 27.5 mg (0.089 mmol, 89% yield) of the title compound as a white solid.

^{1}H NMR (400 MHz, $CDCl_3$) δ 7.92 (d, J = 8.4 Hz, 1H), 7.85 (s, 1H), 7.54 (d, J = 5.5 Hz, 1H), 7.37 – 7.29 (m, 2H), 4.32 – 4.12 (m, 3H), 3.00 – 2.80 (m, 1H), 2.76 – 2.58 (m, 1H), 1.31 (t, J = 7.2 Hz, 3H).

^{13}C NMR (100 MHz, $CDCl_3$) δ 162.9, 140.1, 140.0, 130.2, 128.3, 123.62, 123.60, 123.1, 122.5, 119.3, 113.8 (t, J = 251.1 Hz), 63.5, 40.5 (t, J = 23.7 Hz), 30.7 (t, J = 4.8 Hz), 13.8.

^{19}**F NMR** (376 MHz, $CDCl_3$) δ −104.9 – −105.3 (m).

HRMS *m*/*z* (ESI) calcd. for $C_{15}H_{13}F_2NO_2SNa$ $[M+Na]^+$: 332.0527; found: 332.0543.

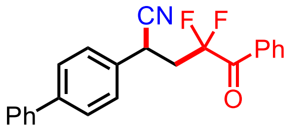

2-([1,1'-Biphenyl]-4-yl)-4,4-difluoro-5-oxo-5-phenylpentanenitrile (40)[11]. The title compound was synthesized according to the General Procedure using 4-vinyl-1,1'-biphenyl (18.0 mg, 0.10 mmol) and 2,2-difluoro-2-iodo-1-phenylethan-1-one (84.6 mg, 0.30 mmol). The crude mixture was purified by flash column chromatography using hexanes/ethyl acetate (10:1 v/v) as the eluent to give 23.1 mg (0.064 mmol, 64% yield) of the title compound as a white solid.

^{1}**H NMR** (400 MHz, $CDCl_3$) δ 8.17 – 8.09 (m, 2H), 7.71 – 7.55 (m, 5H), 7.55 – 7.43 (m, 6H), 7.42 – 7.34 (m, 1H), 4.28 (dd, *J* = 9.8, 4.5 Hz, 1H), 3.07 – 2.89 (m, 1H), 2.88 – 2.67 (m, 1H).

^{19}**F NMR** (376 MHz, $CDCl_3$) δ −96.7 – −102.1 (m).

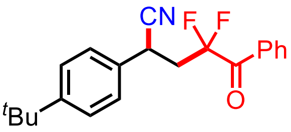

2-(4-(*tert*-Butyl)phenyl)-4,4-difluoro-5-oxo-5-phenylpentanenitrile (41)[11]. The title compound was synthesized according to the General Procedure using 1-(*tert*-butyl)-4-vinylbenzene (16.0 mg, 0.10 mmol) and 2,2-difluoro-2-iodo-1-phenylethan-1-one (84.6 mg, 0.30 mmol). The crude mixture was purified by flash column chromatography using hexanes/ethyl acetate (10:1 v/v) as the eluent to give 24.2 mg (0.071 mmol, 71% yield) of the title compound as a white solid.

^{1}**H NMR** (400 MHz, $CDCl_3$) δ 8.15 – 8.07 (m, 2H), 7.73 – 7.63 (m, 1H), 7.51 (t, *J* = 7.8 Hz, 2H), 7.42 (d, *J* = 8.4 Hz, 2H), 7.34 (d, *J* = 8.4 Hz, 2H), 4.20 (dd, *J* = 10.0, 4.4 Hz, 1H), 3.01 – 2.83 (m, 1H), 2.83 – 2.63 (m, 1H).

^{19}**F NMR** (376 MHz, $CDCl_3$) δ −94.7 – −103.6 (m).

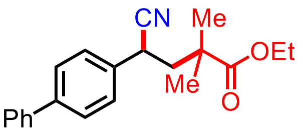

Ethyl-4-([1,1'-biphenyl]-4-yl)-4-cyano-2,2-dimethylbutanoate (42). The title compound was synthesized according to the General Procedure using 4-vinyl-1,1'-biphenyl (18.0 mg, 0.10 mmol) and ethyl-2-bromo-2-methylpropanoate (58.5 mg, 0.30 mmol). After irradiation for 48 hours, the crude mixture was purified by flash column chromatography using hexanes/ethyl acetate (30:1 v/v) as the eluent to give 15.4 mg (0.048 mmol, 48% yield) of the title compound as a colorless oil.

^{1}H NMR (500 MHz, $CDCl_3$) δ 7.60 – 7.56 (m, 4H), 7.46 – 7.41 (m, 4H), 7.38 – 7.34 (m, 1H), 4.17 – 4.06 (m, 2H), 3.89 (dd, J = 9.7, 4.3 Hz, 1H), 2.32 – 2.28 (m, 1H), 2.14 – 2.10 (m, 1H), 1.39 (s, 3H), 1.29 – 1.26 (m, 6H).

^{13}C NMR (125 MHz, $CDCl_3$) δ 176.4, 141.2, 140.3, 135.7, 128.9, 127.8, 127.8, 127.6, 127.1, 121.1, 61.0, 46.2, 42.0, 33.4, 26.0, 25.1, 14.1.

HRMS m/z (ESI) calcd. for $C_{21}H_{23}NO_2Na$ $[M+Na]^+$: 344.1621; found: 344.1623.

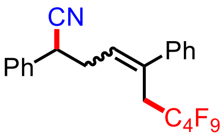

7,7,8,8,9,9,10,10,10-Nonafluoro-2,5-diphenyldec-4-enenitrile (43). The title compound was synthesized according to the General Procedure using (1-(2-phenylcyclopropyl)vinyl)benzene (22.1 mg, 0.10 mmol) and C_4F_9I (103.8 mg, 0.30 mmol). The crude mixture was purified by flash column chromatography using hexanes/ethyl acetate (30:1 v/v) as the eluent to give 33.9 mg (0.073 mmol, 73% yield, E/Z = 3.5:1) of the title compound as a white solid.

^{1}H NMR (500 MHz, $CDCl_3$, major E-isomer) δ 7.46 – 7.26 (m, 10H), 6.05 (t, J = 7.6 Hz, 1H), 3.94 (t, J = 7.0 Hz, 1H), 3.19 – 3.07 (m, 2H), 2.88 – 2.75 (m, 2H).

^{13}C NMR (125 MHz, $CDCl_3$, major E-isomer) δ 141.6, 134.8, 132.9, 129.9, 129.2, 129.0, 128.5, 127.9, 127.3, 126.4, 120.0, 37.2, 35.4, 31.20 (t, J = 21.7 Hz).

^{19}F NMR (471 MHz, $CDCl_3$) δ −80.6 – −81.4 (m, 3F), −111.4 – −111.5 (m, 2F), −124.1 – −124.2 (m, 2F), −125.8 – −125.9 (m, 2F).

HRMS *m/z* (ESI) calcd. for $C_{22}H_{16}F_9NNa$ $[M+Na]^+$: 488.1031; found: 488.1030.

5. Supplementary references

1. Gaussian 16 Rev. A.03 (Wallingford, CT, 2016).
2. Adamo, C. & Barone, V. Toward reliable density functional methods without adjustable parameters: the PBE0 model. *J. Chem. Phys.* **110**, 6158–6170 (1999).
3. Grimme, S., Antony, J., Ehrlich, S. & Krieg, H. A consistent and accurate *ab* initio parametrization of density functional dispersion correction (DFT-D) for the 94 elements H-Pu. *J. Chem. Phys.* **132**, 154104 (2010).
4. Grimme, S., Ehrlich, S. & Goerigk, L. Effect of the damping function in dispersion corrected density functional theory. *J. Comput. Chem.* **32**, 1456−1465 (2011).
5. Lu, T. & Chen, F. Multiwfn: a multifunctional wavefunction analyzer. *J. Comput. Chem.* **33**, 580–592 (2012).
6. Lu, T. & Chen, F. Calculation of molecular orbital composition. *Acta Chim. Sinica* **69**, 2393−2406 (2011).
7. Israr, M., Xiong, H., Li, Y. & Bao, H. Copper-catalyzed enantioselective cyano(fluoro)alkylation of alkenes. *Adv. Synth. Catal.* **362**, 2211–2215 (2020).
8. Guo, Q., Wang, M., Wang, Y., Xu, Z. & Wang, R. Photoinduced, copper–catalyzed three components cyanofluoroalkylation of alkenes with fluoroalkyl iodides as fluoroalkylation reagents. *Chem. Commun.* **53**, 12317-12320 (2017).
9. Wang, F. *et al.* Enantioselective copper-catalyzed intermolecular cyanotrifluoromethylation of alkenes via radical process. *J. Am. Chem. Soc.* **138**, 15547−15550 (2016).
10. Rao, N., Li, Y.-Z., Luo, Y.-C., Zhang, Y. & Zhang, X. Nickel-catalyzed multicomponent carbodifluoroalkylation of electron-deficient alkenes. *ACS Catal.* **13**, 4111−4119 (2023).
11. Fang, M. *et al.* Enantioselective copper-catalyzed intermolecular cyanobenzoyldifluoromethylation of alkenes: access to chiral β-difluoroacyl nitriles. *J. Org. Chem.* **87**, 4107−4111 (2022)

6. NMR spectra

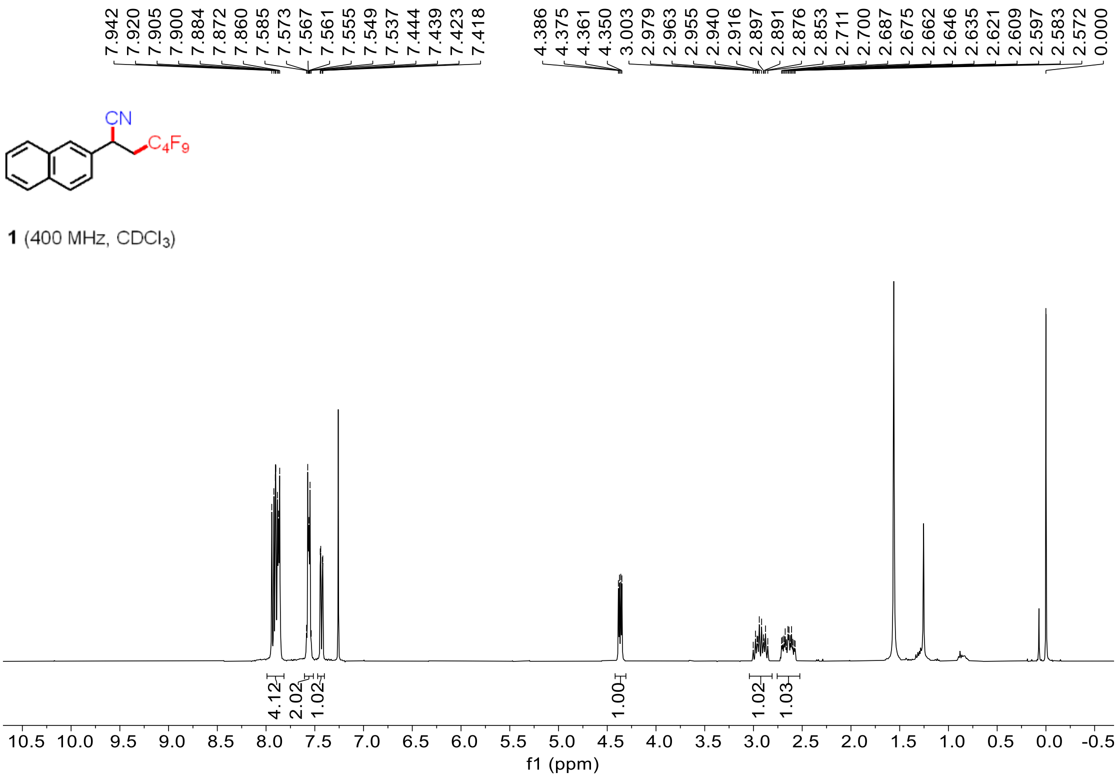

1 (400 MHz, $CDCl_3$)

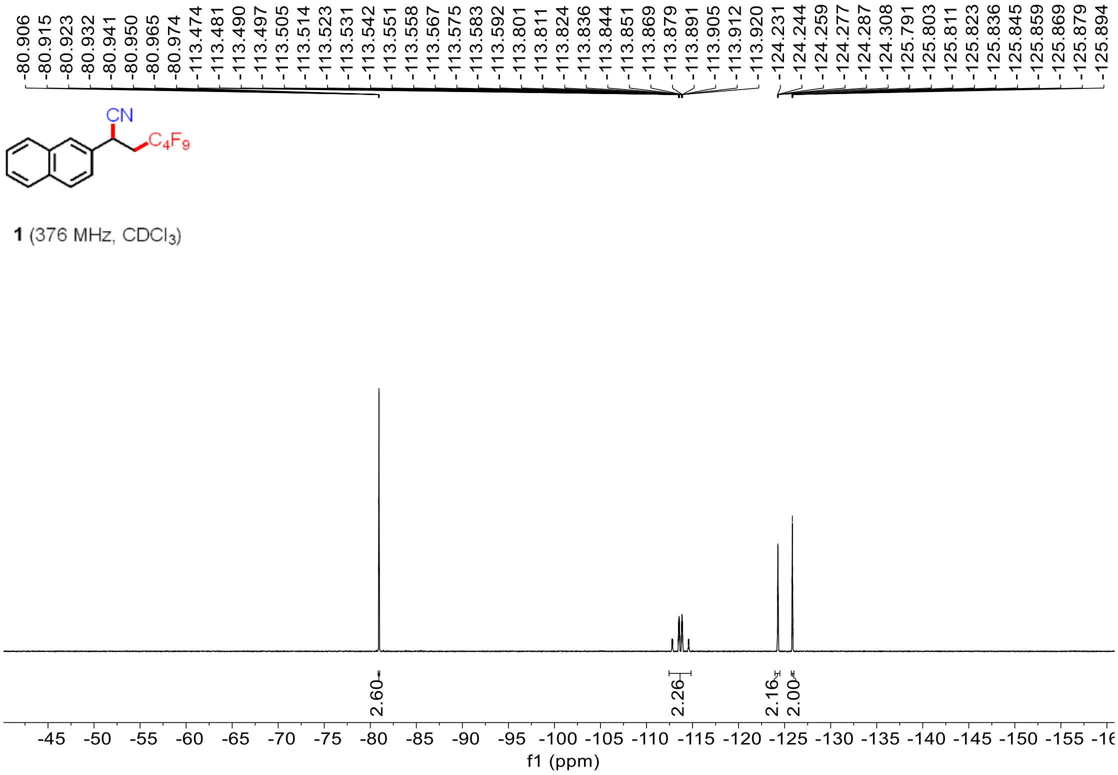

1 (376 MHz, $CDCl_3$)

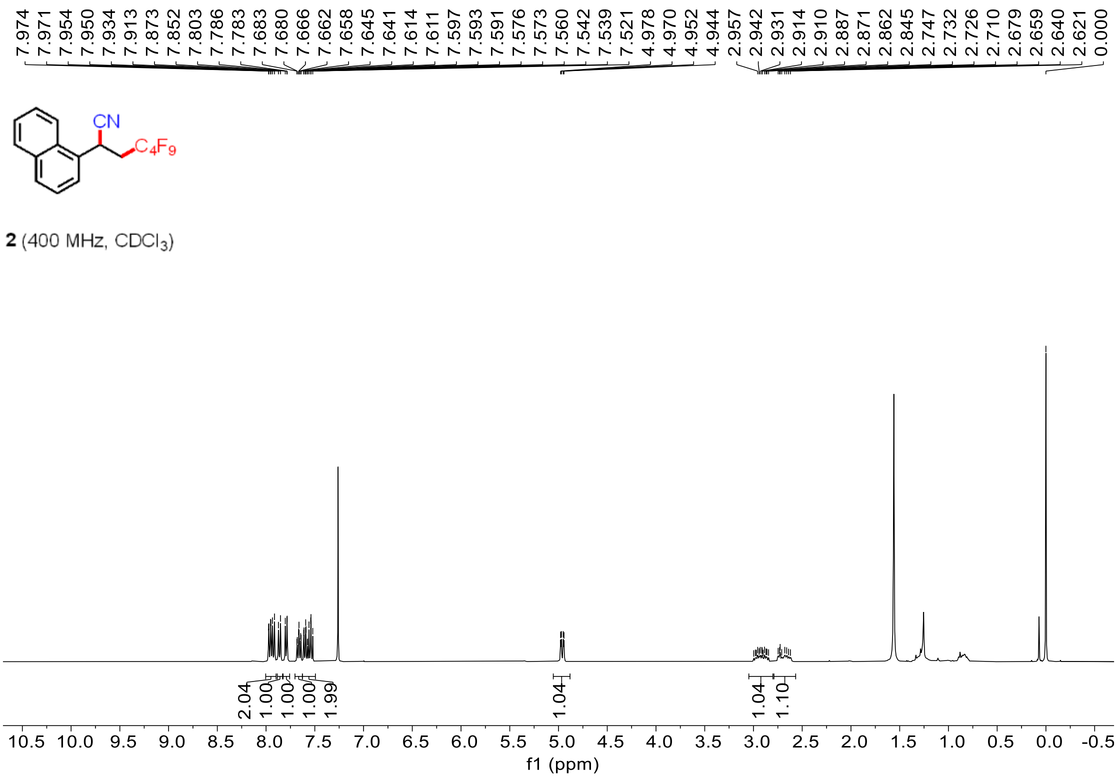

2 (400 MHz, $CDCl_3$)

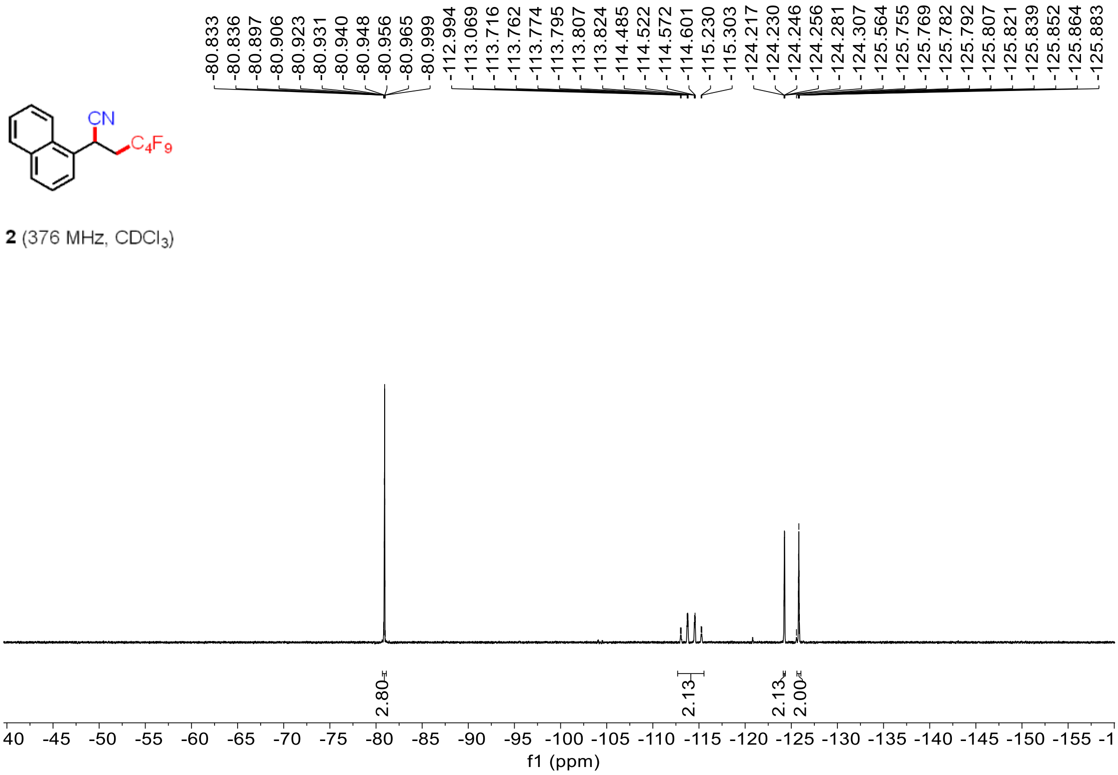

2 (376 MHz, $CDCl_3$)

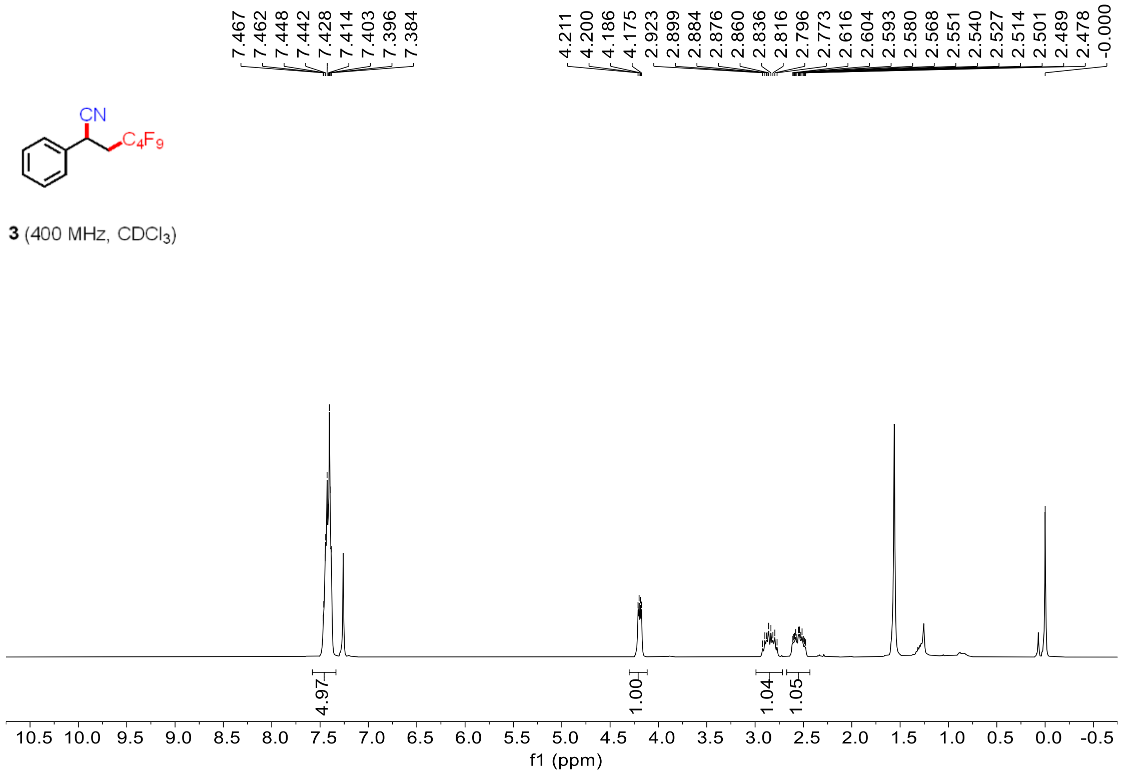

CN
C4F9
3 (400 MHz, CDCl3)

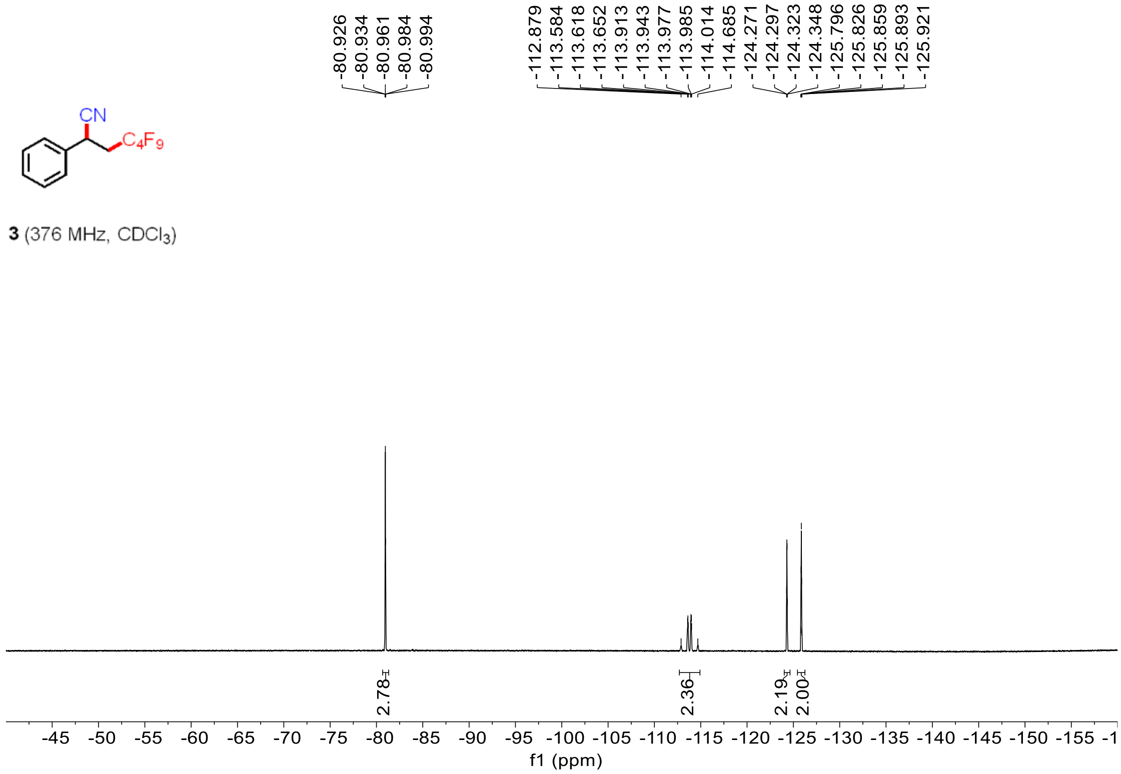

CN
C4F9
3 (376 MHz, CDCl3)

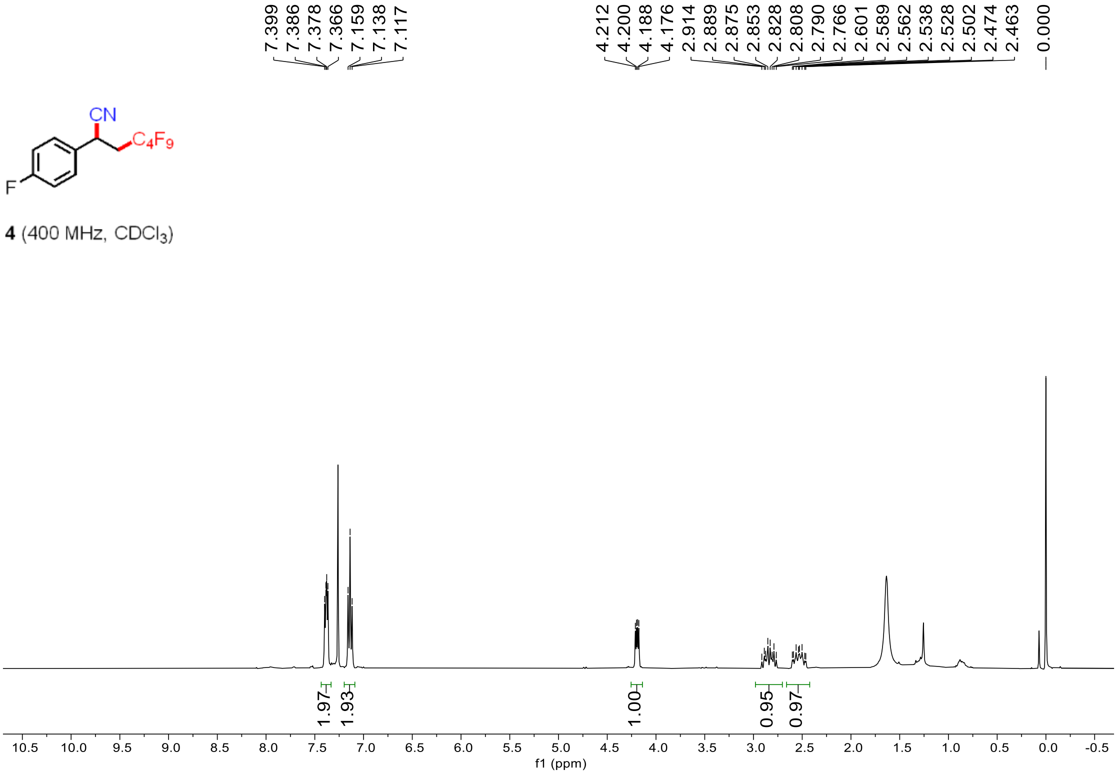

4 (400 MHz, $CDCl_3$)

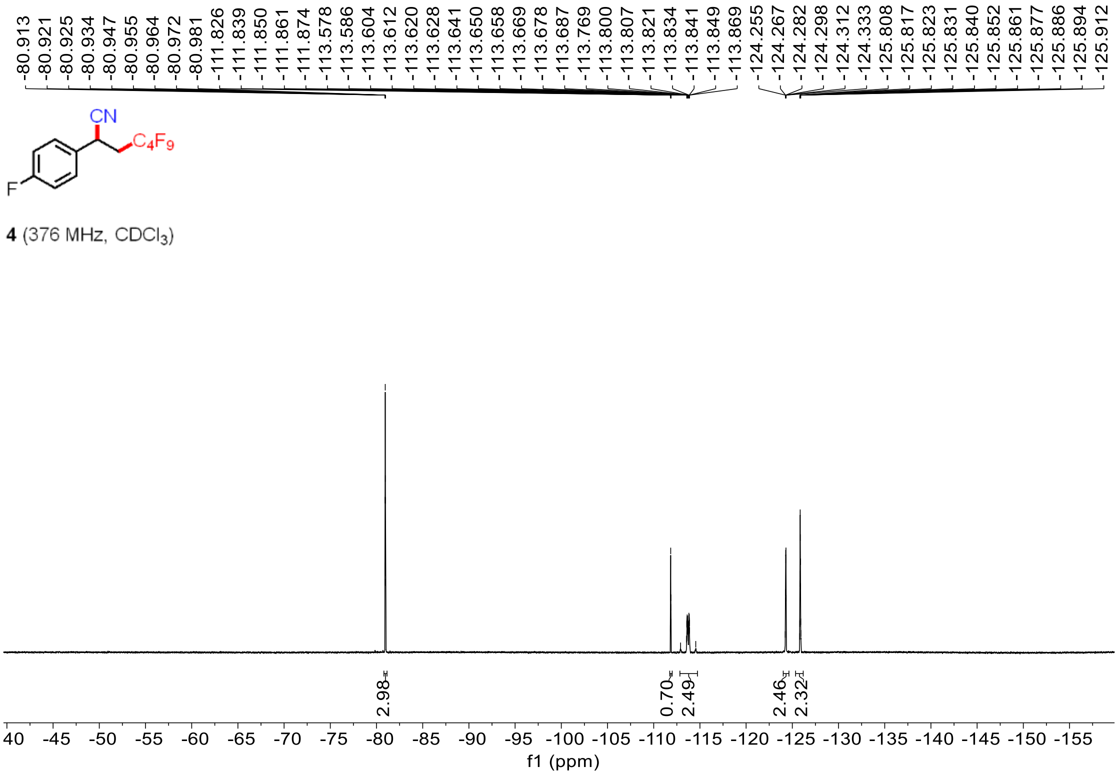

4 (376 MHz, $CDCl_3$)

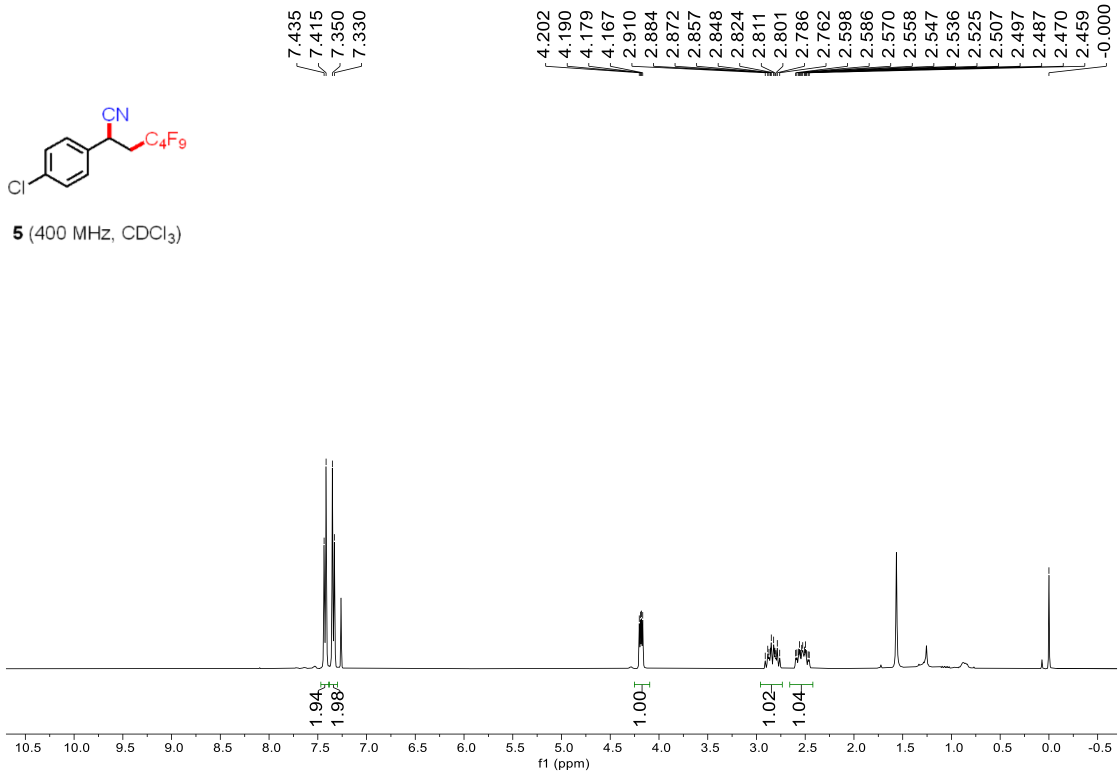

5 (400 MHz, $CDCl_3$)

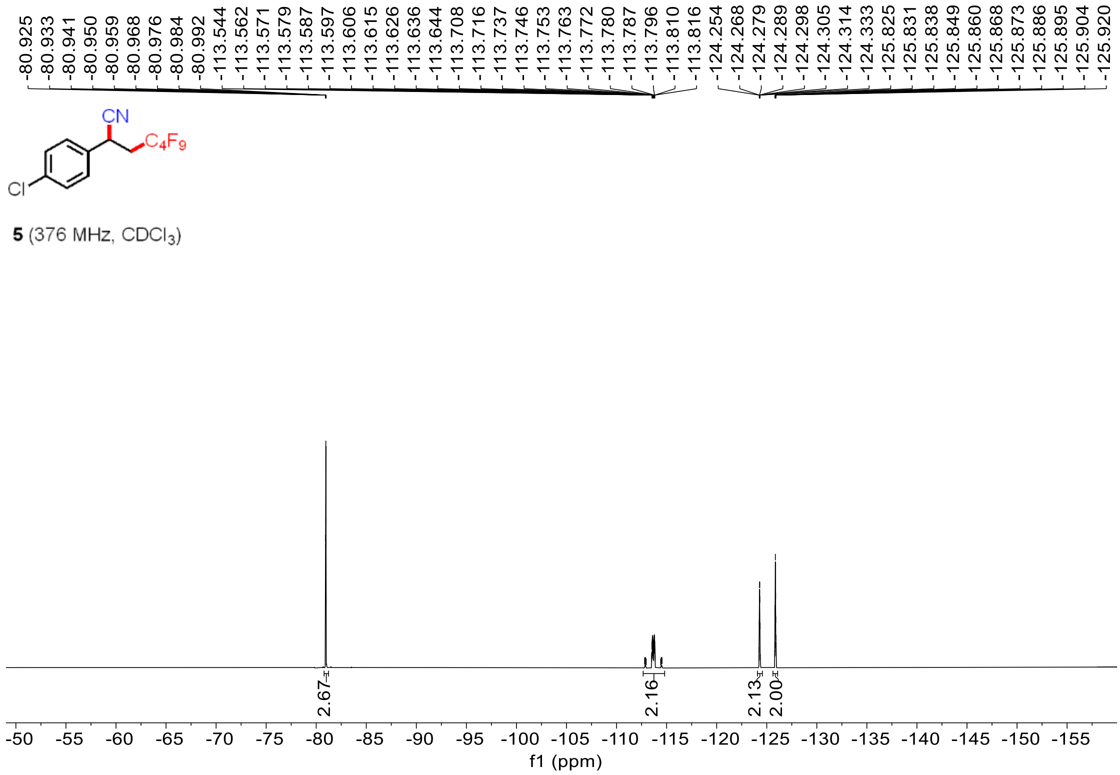

5 (376 MHz, $CDCl_3$)

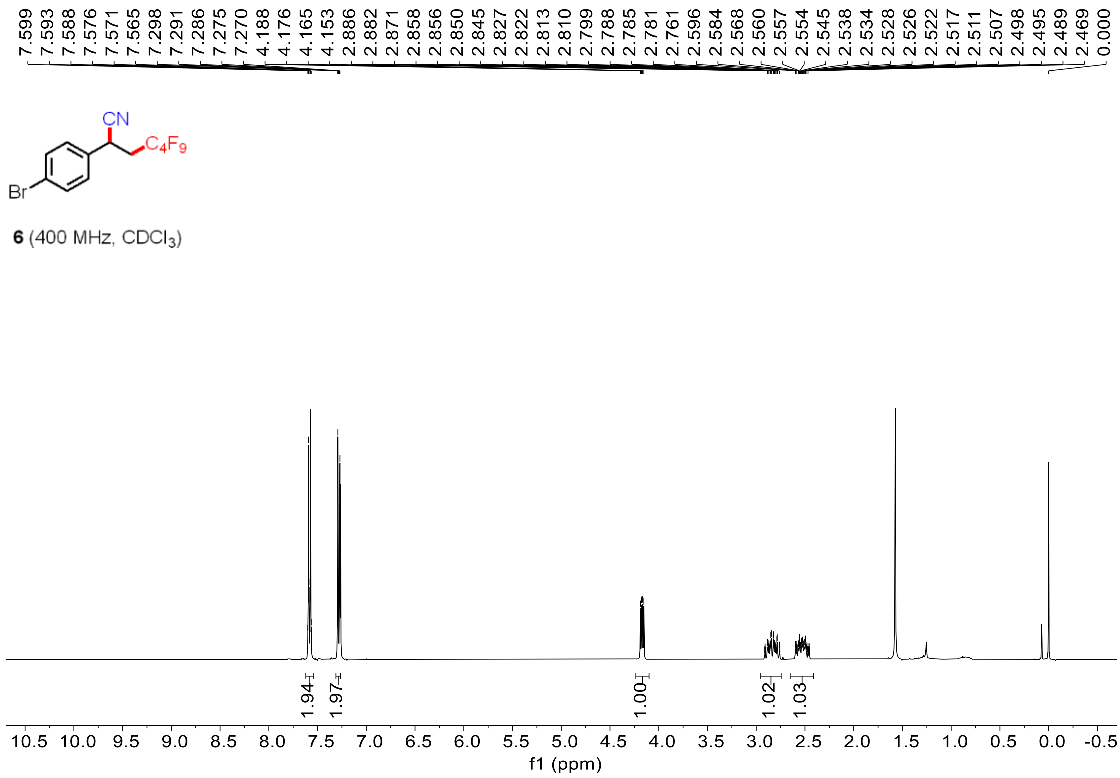

6 (400 MHz, $CDCl_3$)

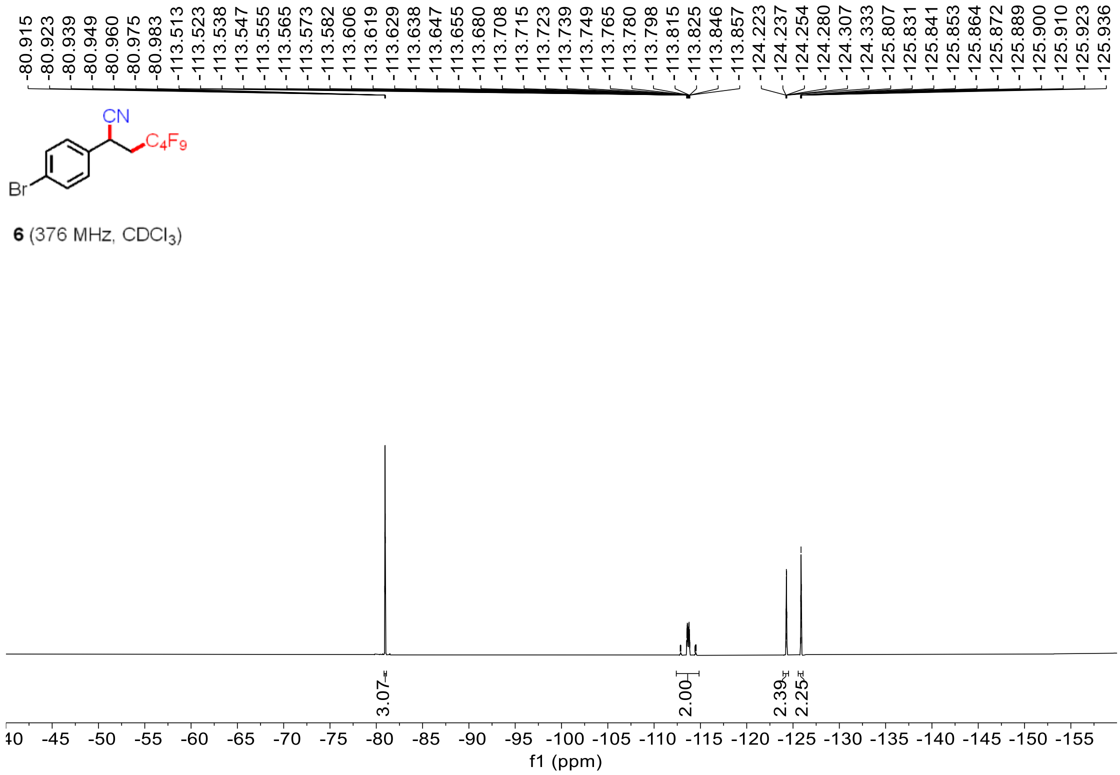

6 (376 MHz, $CDCl_3$)

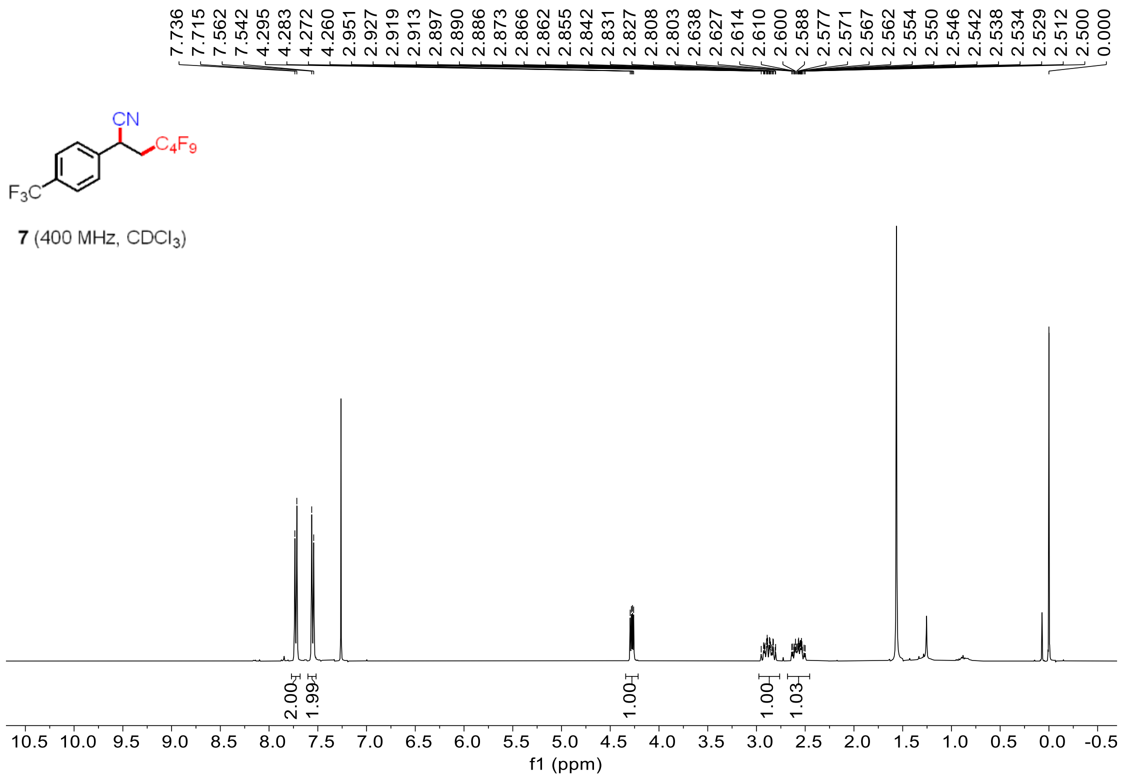

7 (400 MHz, $CDCl_3$)

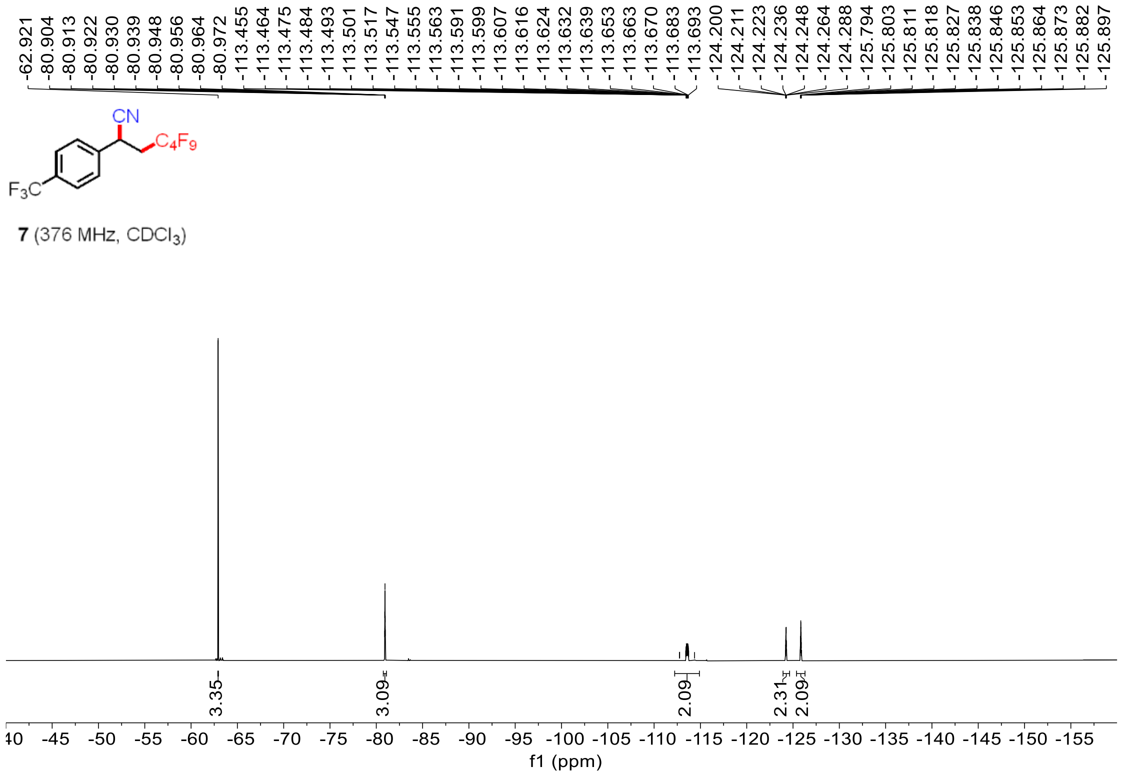

7 (376 MHz, $CDCl_3$)

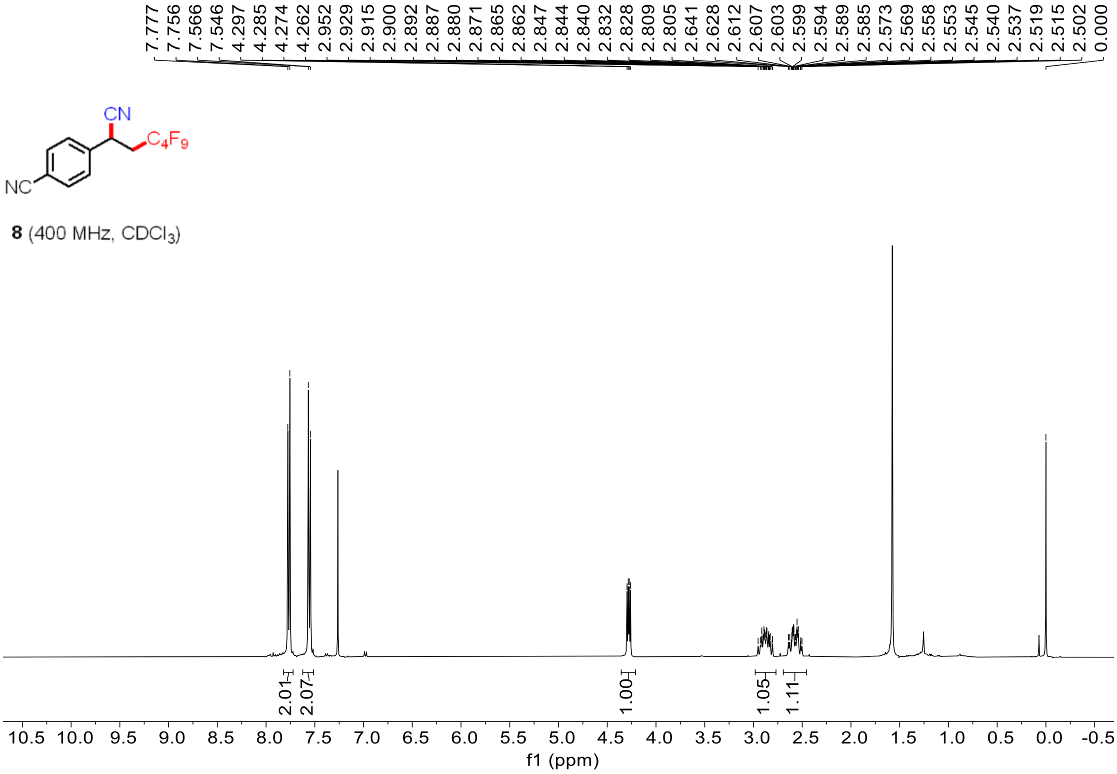
7.777
7.756
7.566
7.546
4.297
4.285
4.274
4.262
2.952
2.929
2.915
2.900
2.892
2.887
2.880
2.871
2.865
2.862
2.847
2.844
2.840
2.832
2.828
2.809
2.805
2.641
2.628
2.612
2.607
2.603
2.599
2.594
2.589
2.585
2.573
2.569
2.558
2.553
2.545
2.540
2.537
2.519
2.515
2.502
0.000
CN
C4F9
NC
8 (400 MHz, CDCl3)
2.01
2.07
1.00
1.05
1.11
10.5 10.0 9.5 9.0 8.5 8.0 7.5 7.0 6.5 6.0 5.5 5.0 4.5 4.0 3.5 3.0 2.5 2.0 1.5 1.0 0.5 0.0 -0.5
f1 (ppm)

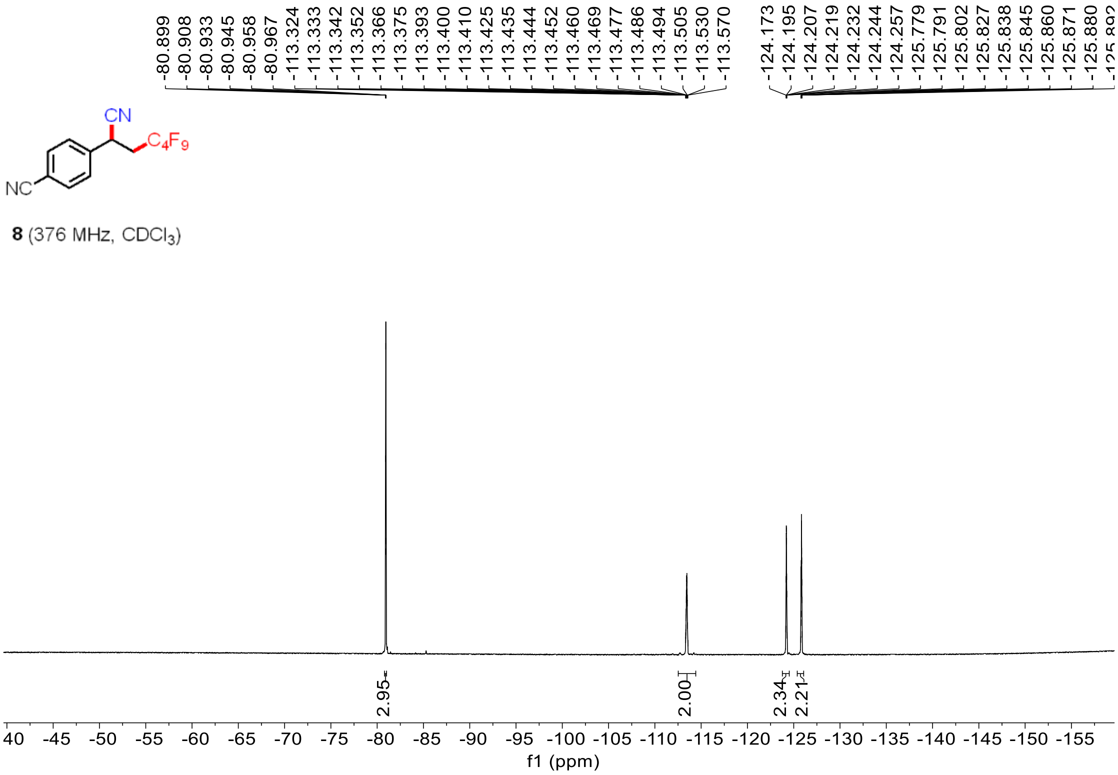
-80.899
-80.908
-80.933
-80.945
-80.958
-80.967
-113.324
-113.333
-113.342
-113.352
-113.366
-113.375
-113.393
-113.400
-113.410
-113.425
-113.435
-113.444
-113.452
-113.460
-113.469
-113.477
-113.486
-113.494
-113.505
-113.530
-113.570
-124.173
-124.195
-124.207
-124.219
-124.232
-124.244
-124.257
-125.779
-125.791
-125.802
-125.827
-125.838
-125.845
-125.860
-125.871
-125.880
CN
C4F9
NC
8 (376 MHz, CDCl3)
2.95
2.00
2.34
2.21
40 -45 -50 -55 -60 -65 -70 -75 -80 -85 -90 -95 -100 -105 -110 -115 -120 -125 -130 -135 -140 -145 -150 -155
f1 (ppm)

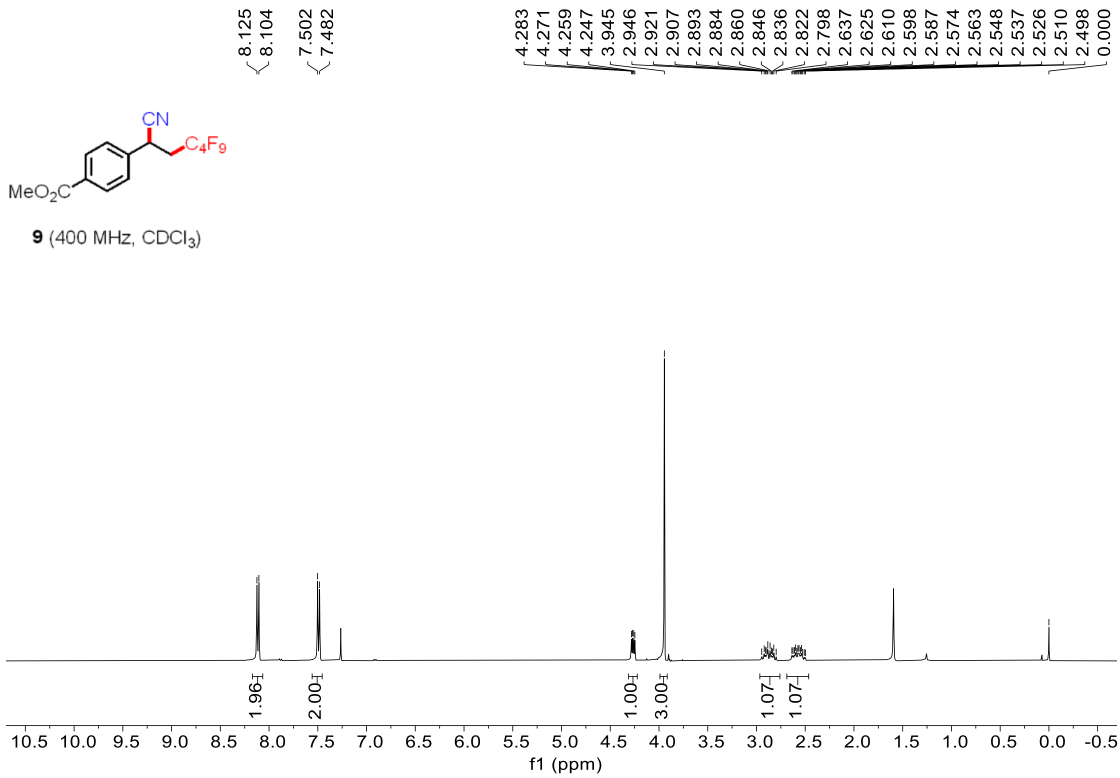

9 (400 MHz, $CDCl_3$)

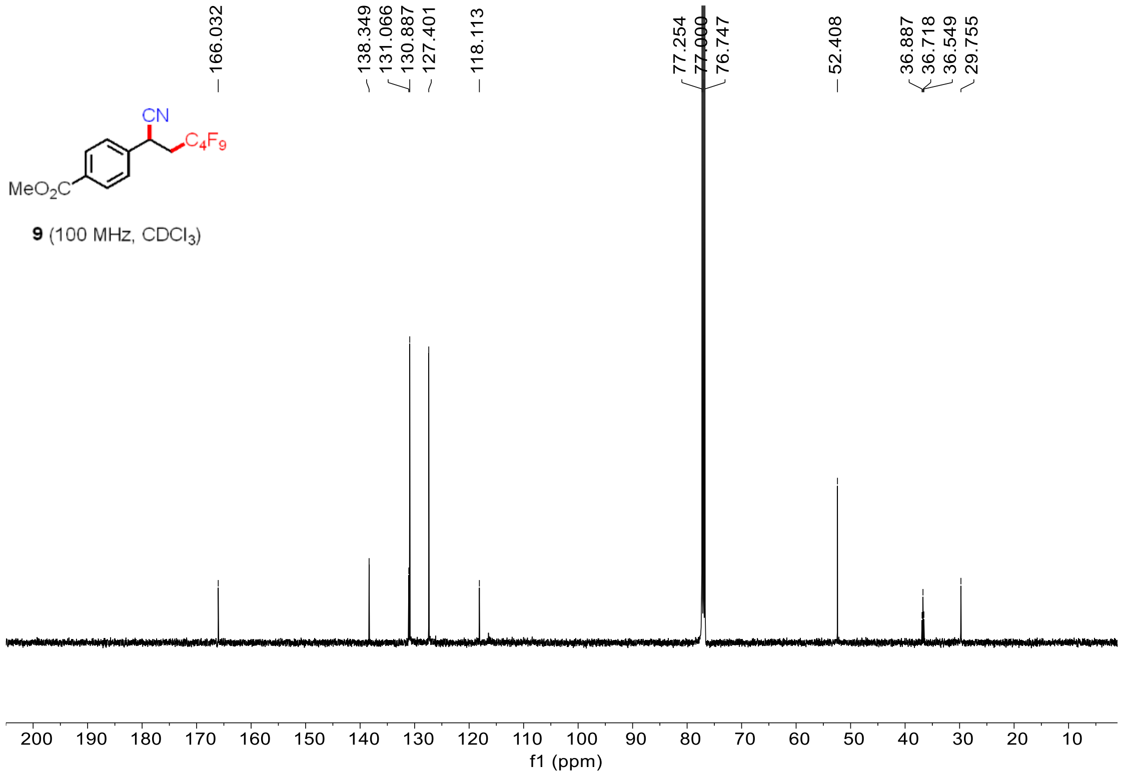

9 (100 MHz, $CDCl_3$)

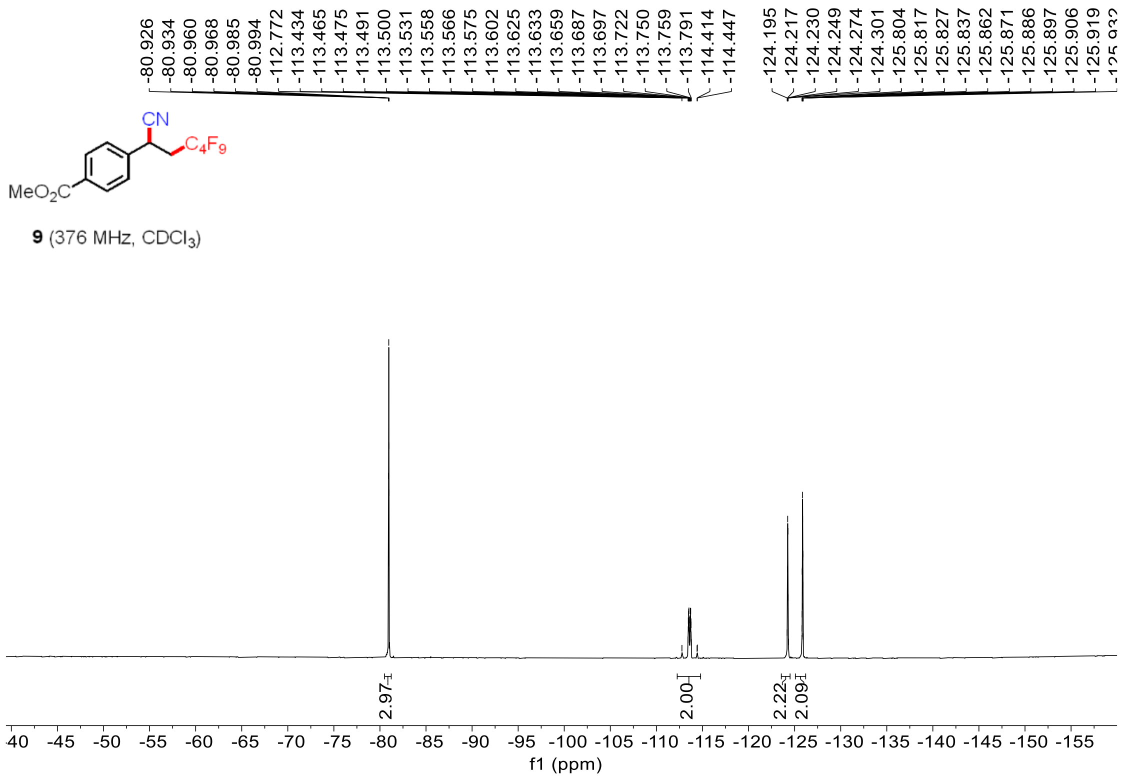

9 (376 MHz, $CDCl_3$)

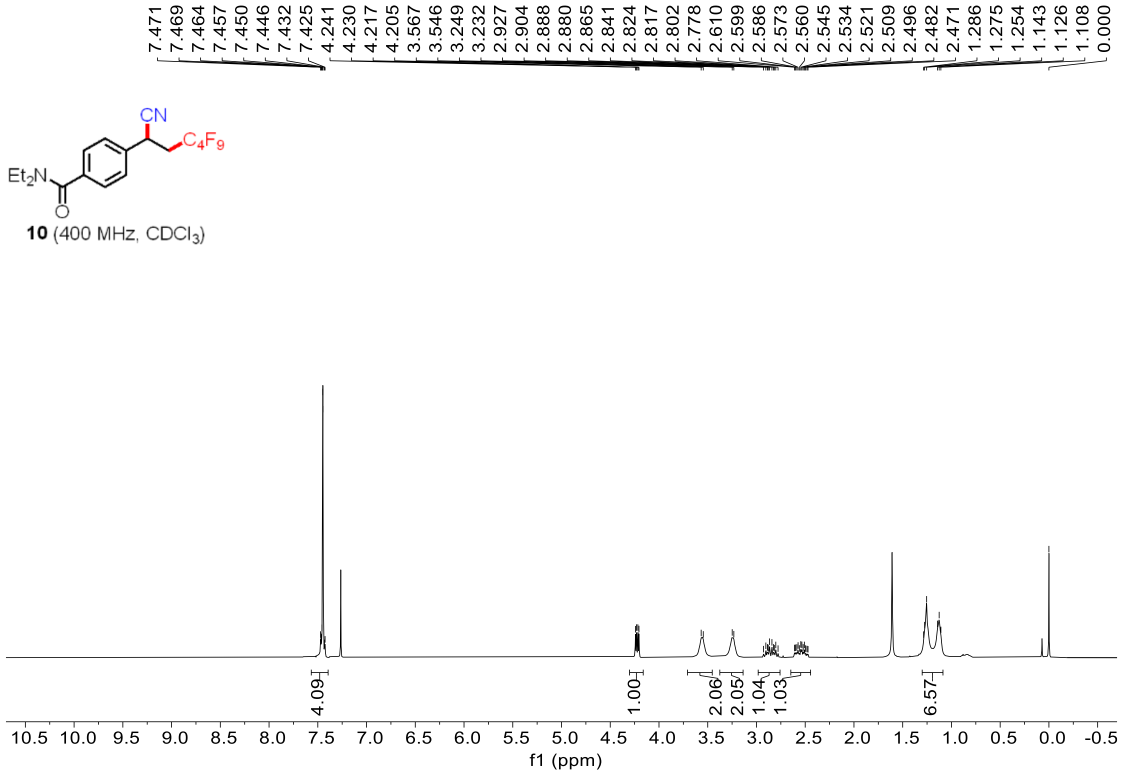

10 (400 MHz, $CDCl_3$)

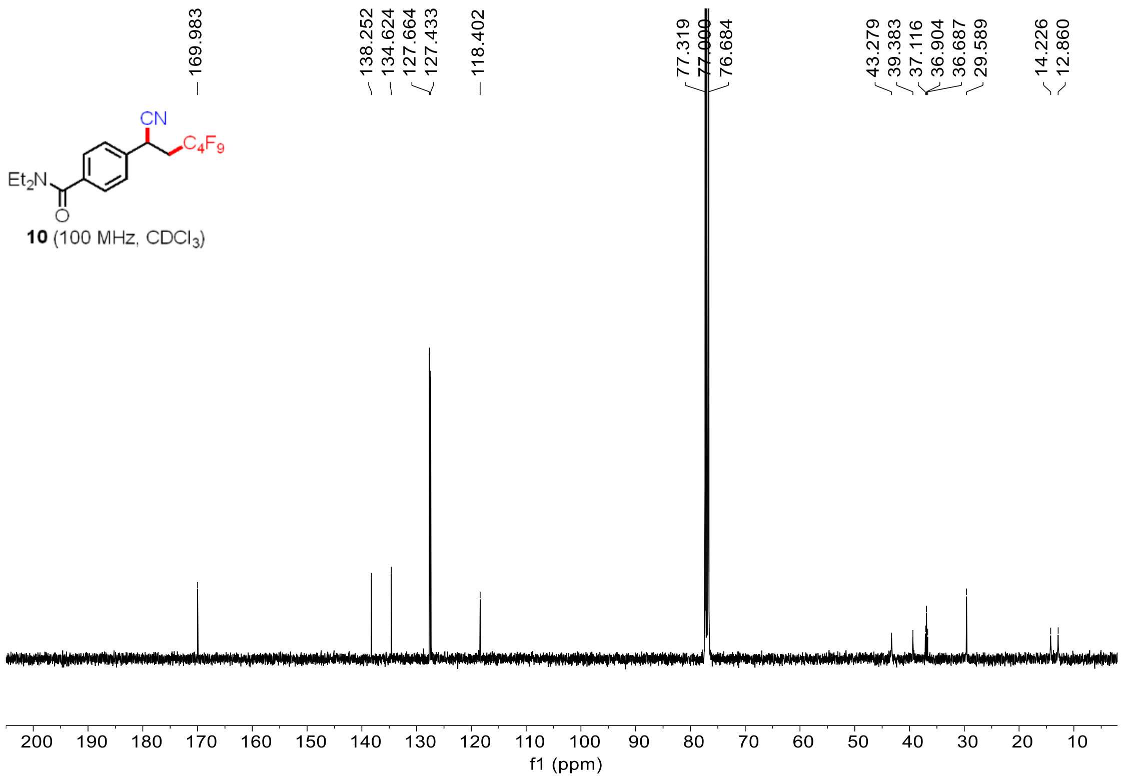
169.983
138.252
134.624
127.664
127.433
118.402
77.319
77.000
76.684
43.279
39.383
37.116
36.904
36.687
29.589
14.226
12.860
CN
C4F9
Et2N
O
10 (100 MHz, CDCl3)
200 190 180 170 160 150 140 130 120 110 100 90 80 70 60 50 40 30 20 10
f1 (ppm)

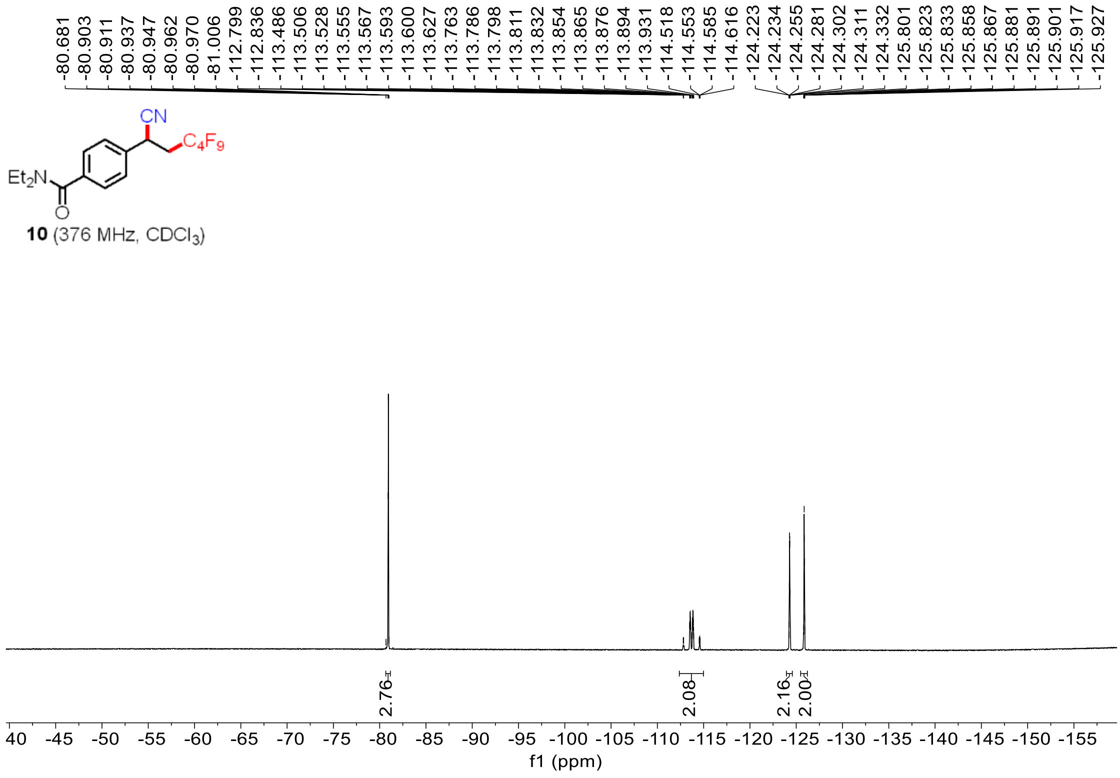
-80.681
-80.903
-80.911
-80.937
-80.947
-80.962
-80.970
-81.006
-112.799
-112.836
-113.486
-113.506
-113.528
-113.555
-113.567
-113.593
-113.600
-113.627
-113.763
-113.786
-113.798
-113.811
-113.832
-113.854
-113.865
-113.876
-113.894
-113.931
-114.518
-114.553
-114.585
-114.616
-124.223
-124.234
-124.255
-124.281
-124.302
-124.311
-124.332
-125.801
-125.823
-125.833
-125.858
-125.867
-125.881
-125.891
-125.901
-125.917
-125.927
CN
C4F9
Et2N
O
10 (376 MHz, CDCl3)
2.76
2.08
2.16
2.00
40 -45 -50 -55 -60 -65 -70 -75 -80 -85 -90 -95 -100 -105 -110 -115 -120 -125 -130 -135 -140 -145 -150 -155
f1 (ppm)

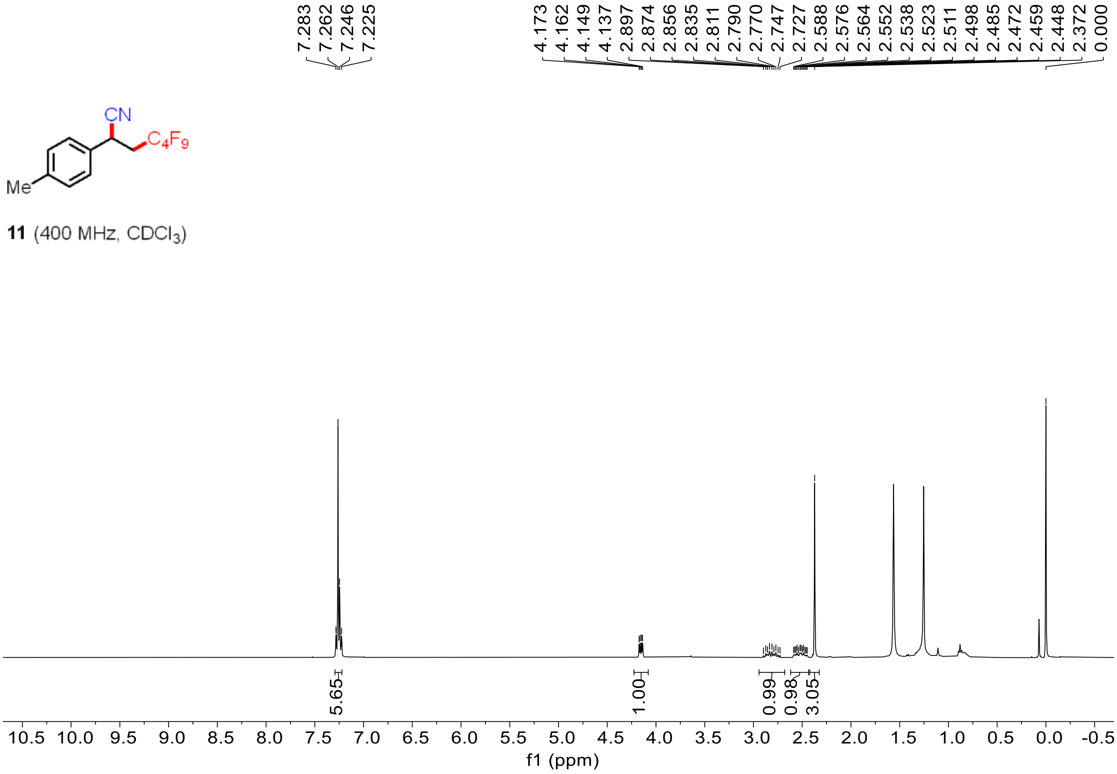

11 (400 MHz, $CDCl_3$)

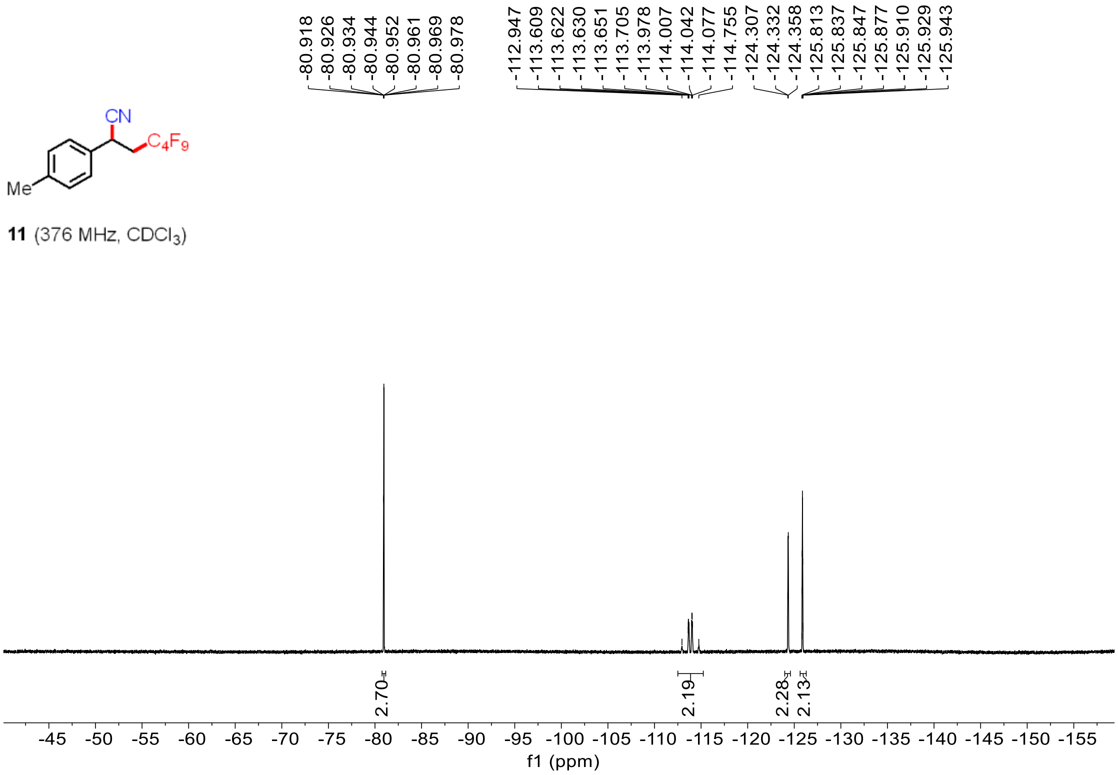

11 (376 MHz, $CDCl_3$)

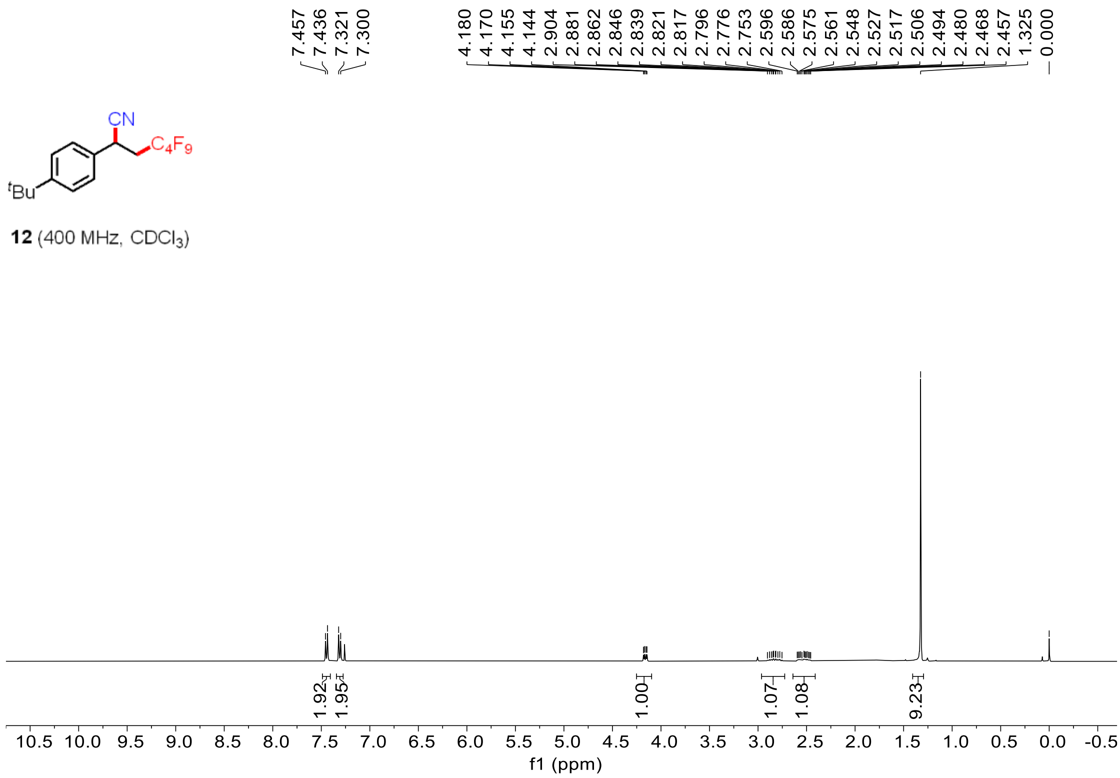
CN
C_4F_9
^{t}Bu
12 (400 MHz, $CDCl_3$)

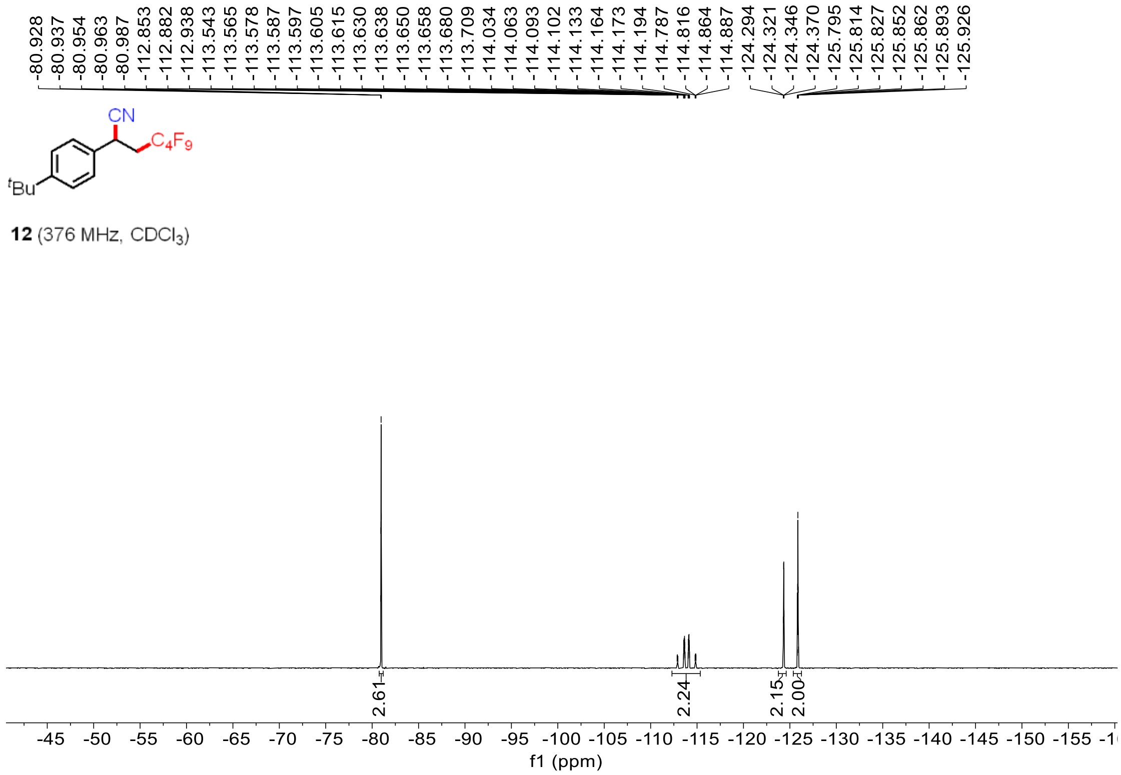
CN
C_4F_9
^{t}Bu
12 (376 MHz, $CDCl_3$)

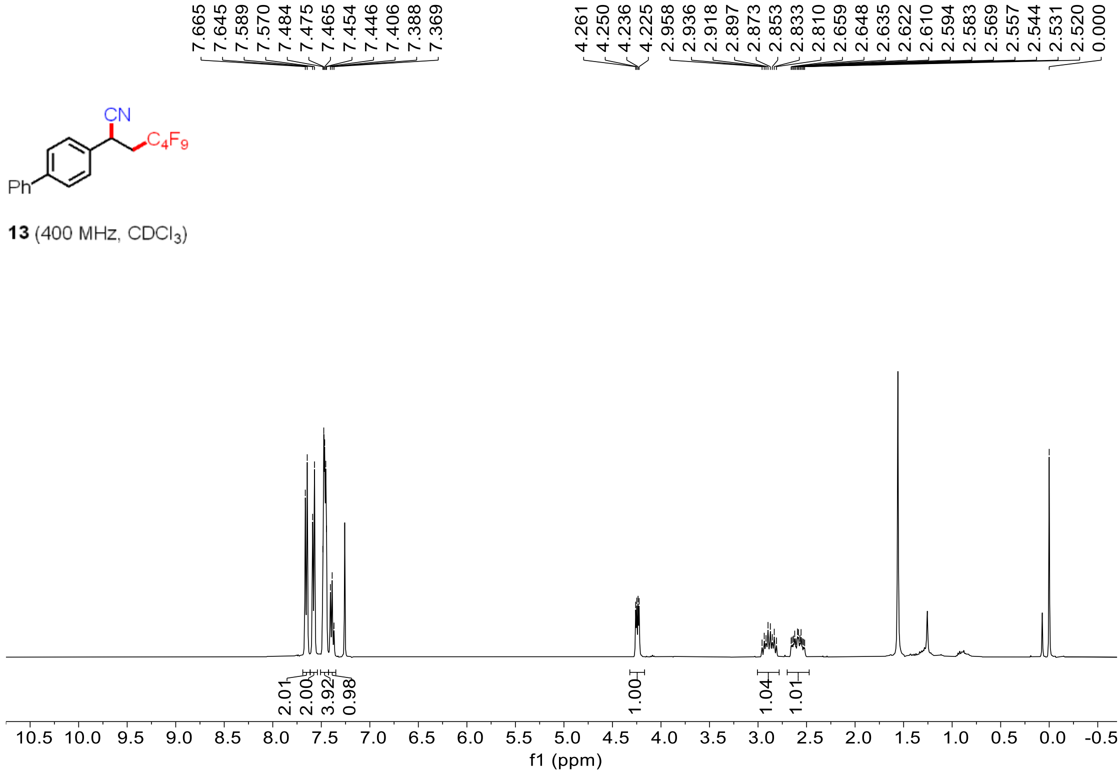

13 (400 MHz, $CDCl_3$)

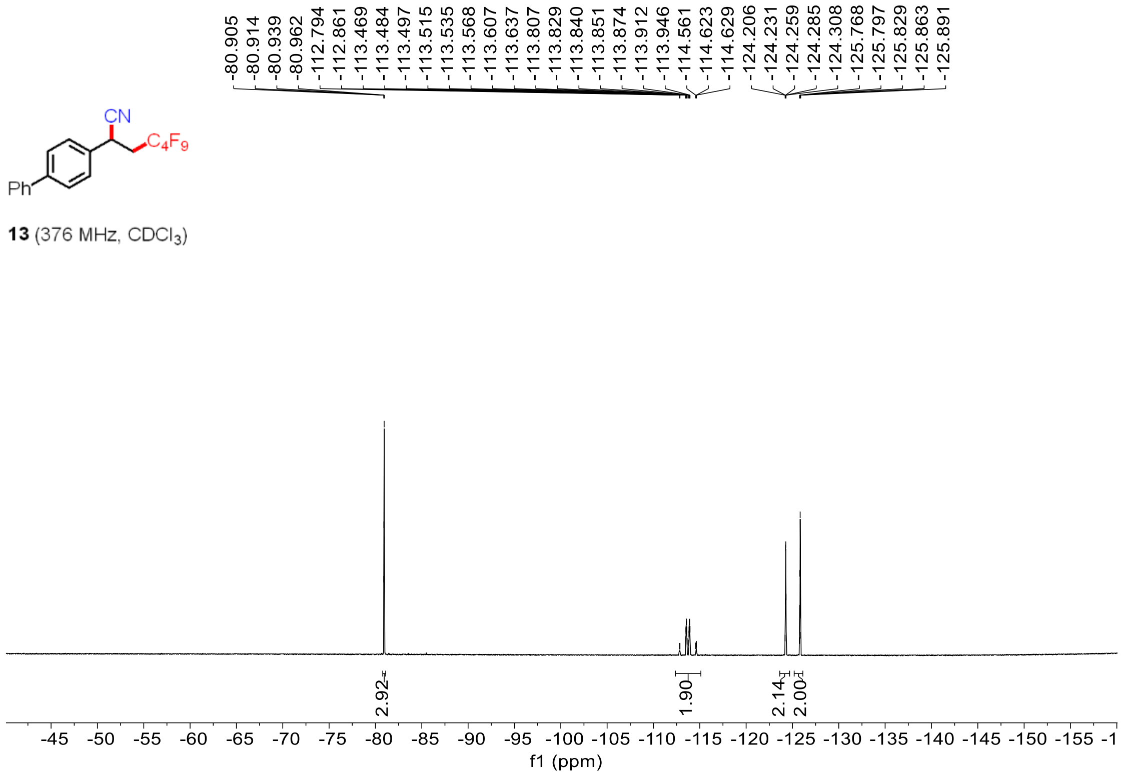

13 (376 MHz, $CDCl_3$)

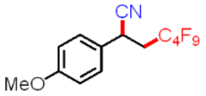

14 (400 MHz, $CDCl_3$)

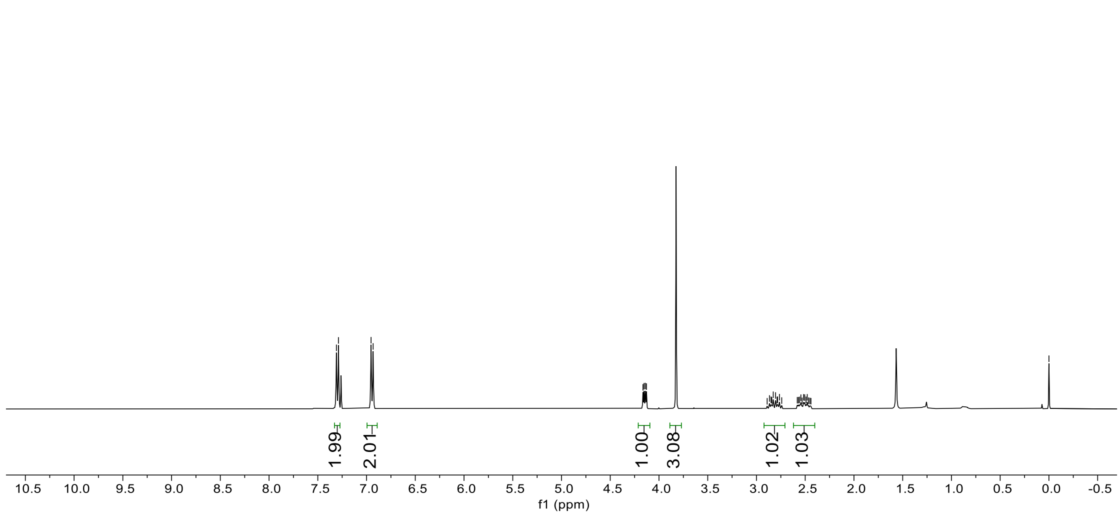

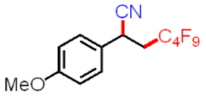

14 (376 MHz, $CDCl_3$)

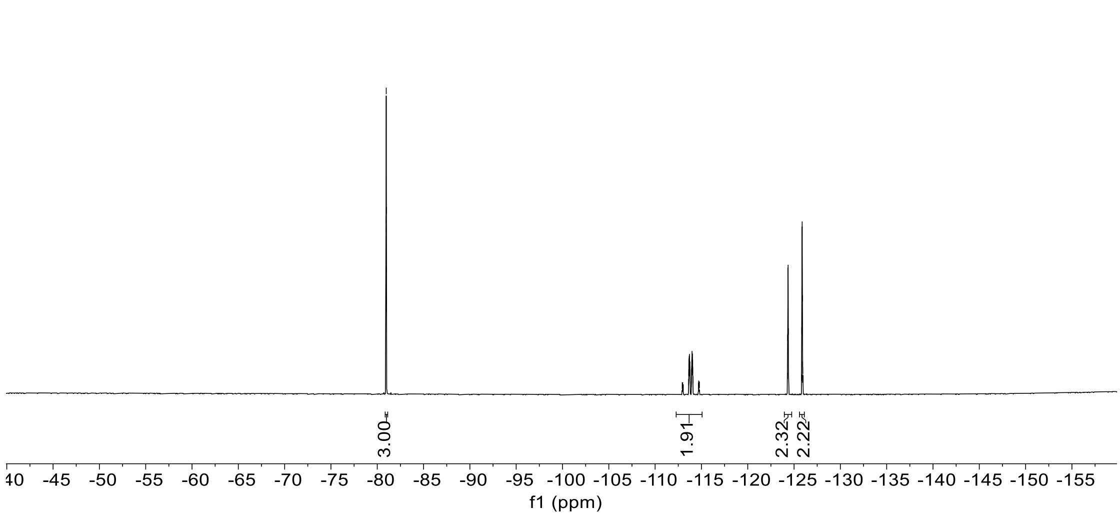

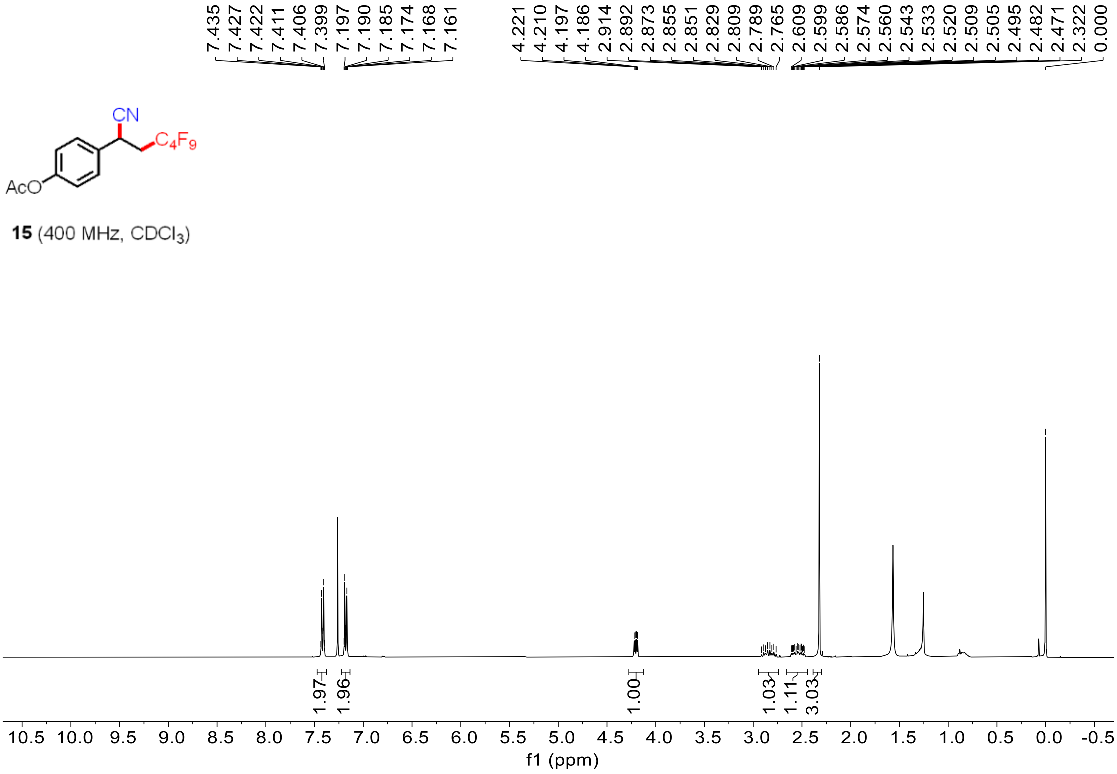

15 (400 MHz, $CDCl_3$)

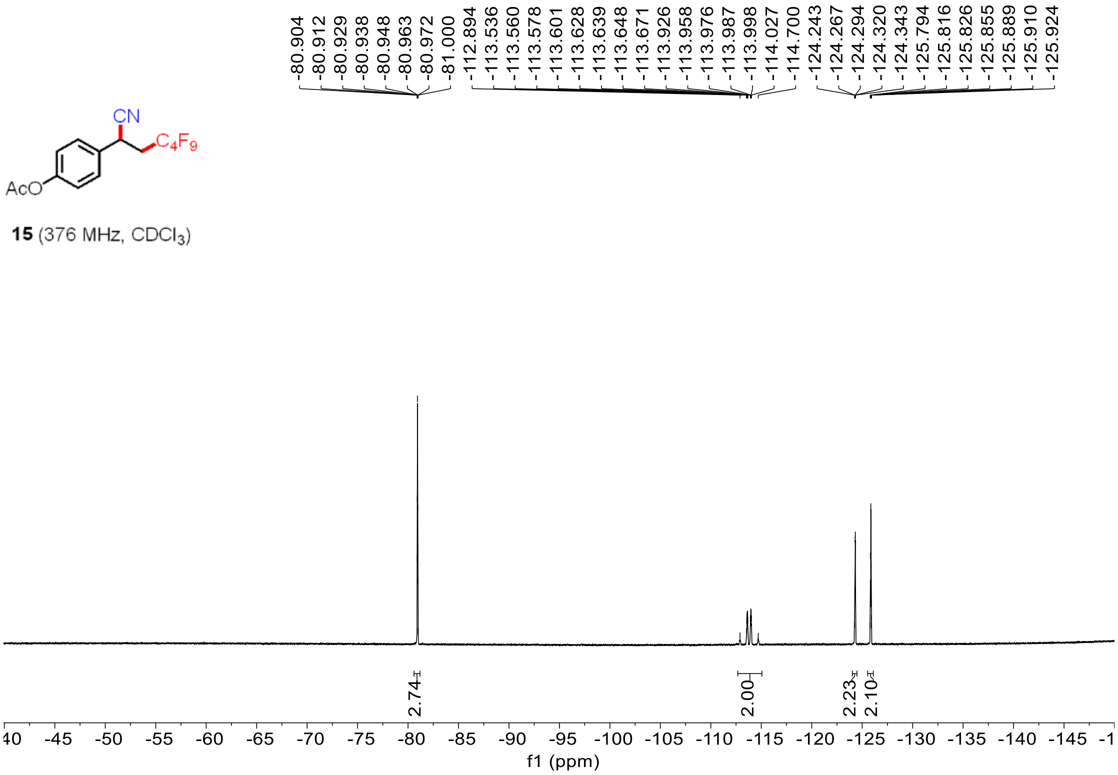

15 (376 MHz, $CDCl_3$)

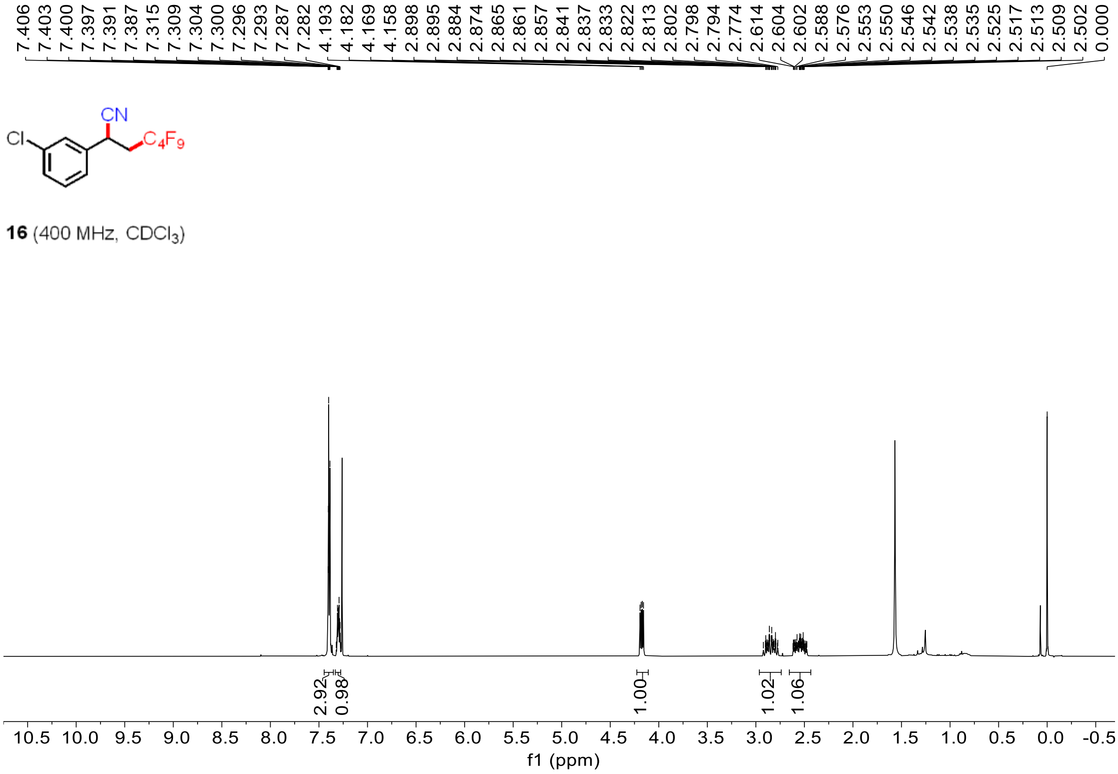

16 (400 MHz, $CDCl_3$)

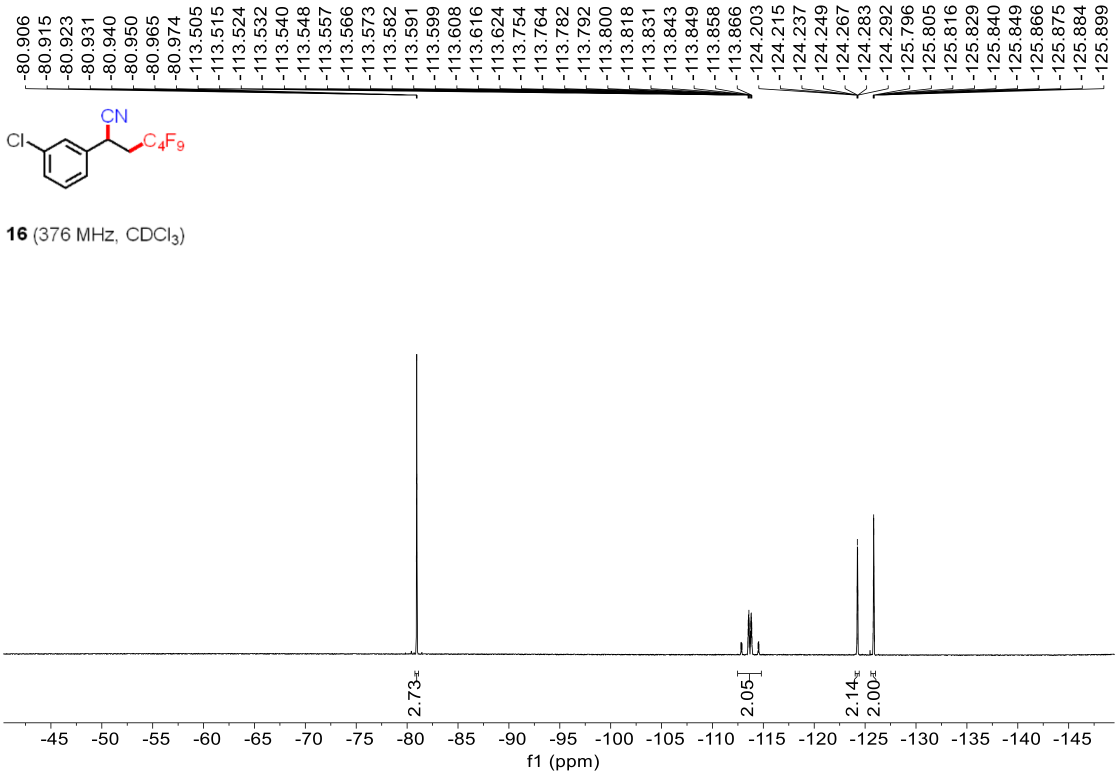

16 (376 MHz, $CDCl_3$)

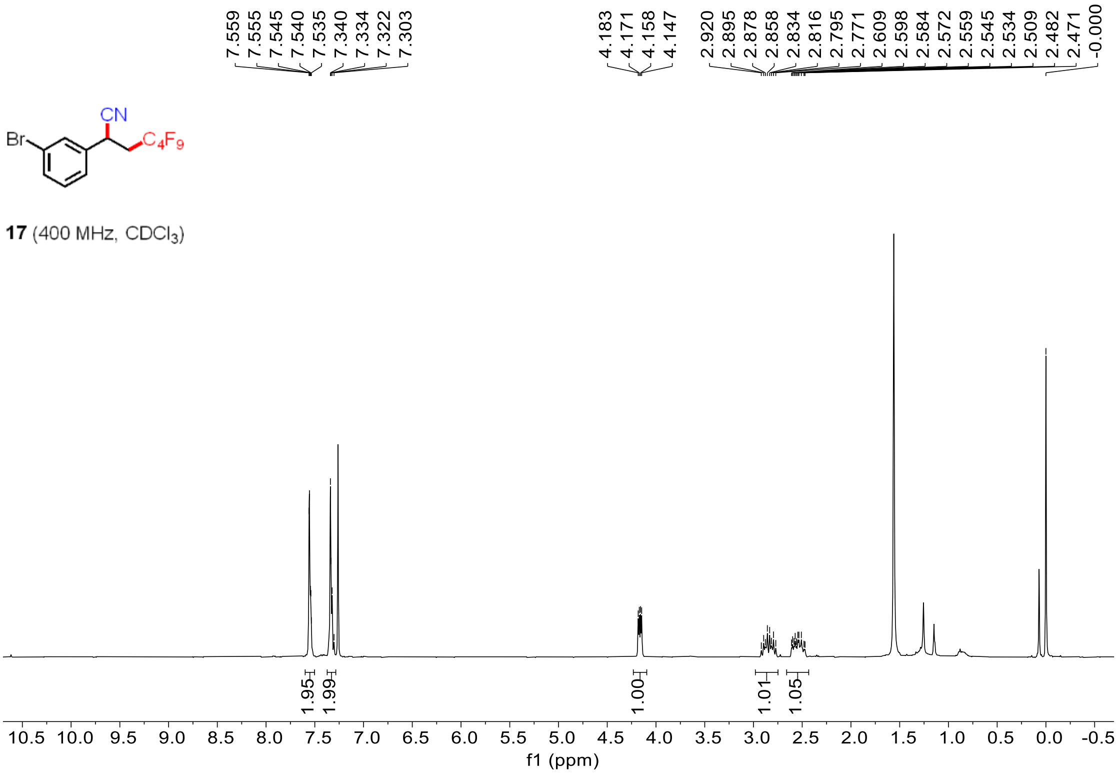

17 (400 MHz, $CDCl_3$)

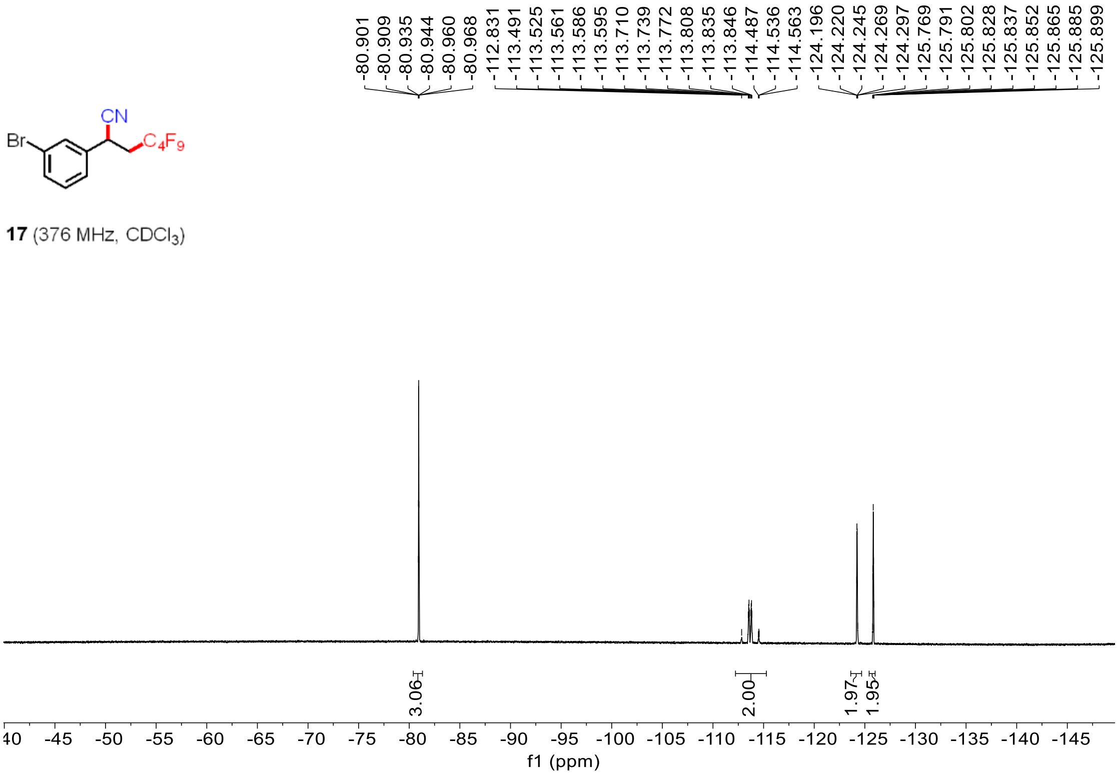

17 (376 MHz, $CDCl_3$)

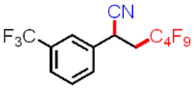

18 (400 MHz, $CDCl_3$)

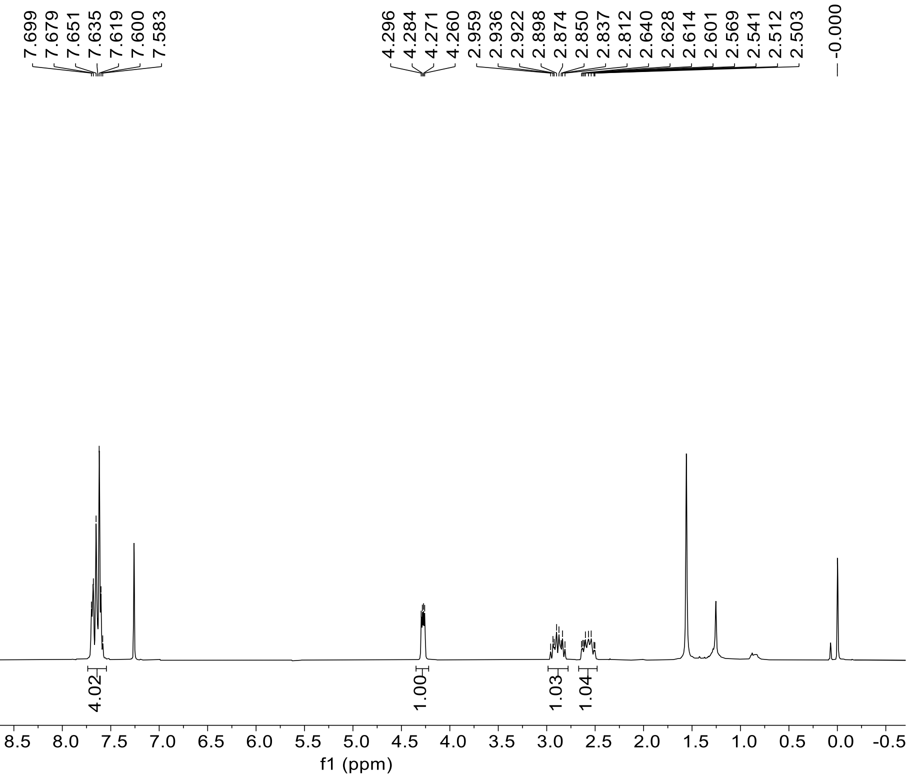

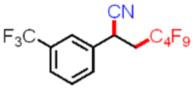

18 (100 MHz, $CDCl_3$)

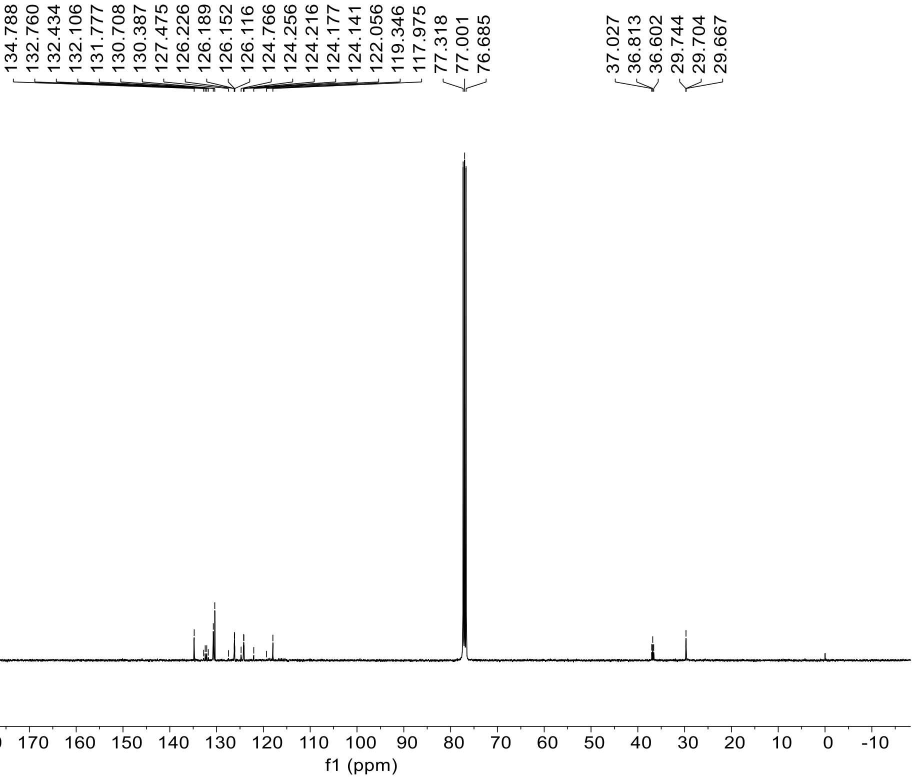

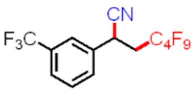

18 (376 MHz, $CDCl_3$)

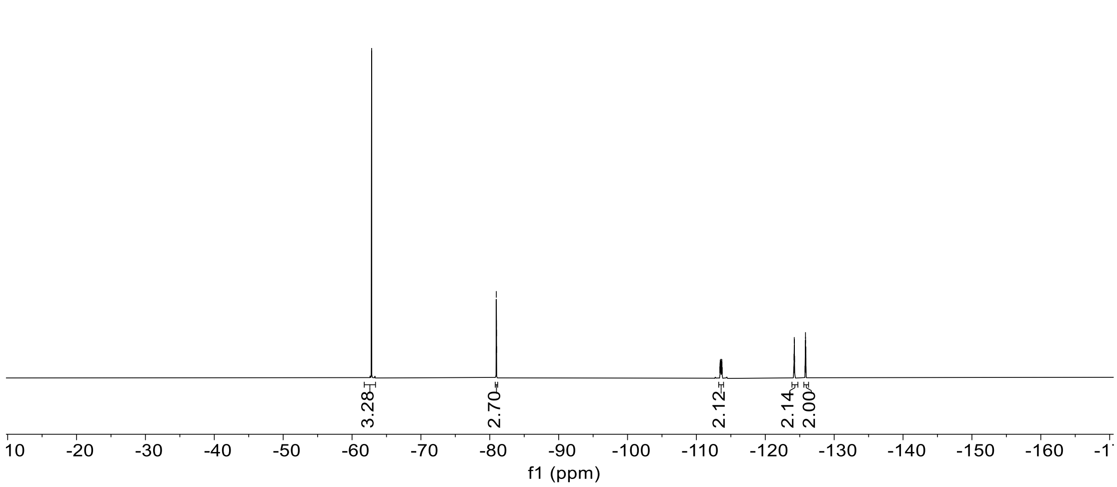

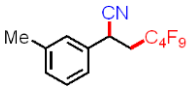

19 (400 MHz, $CDCl_3$)

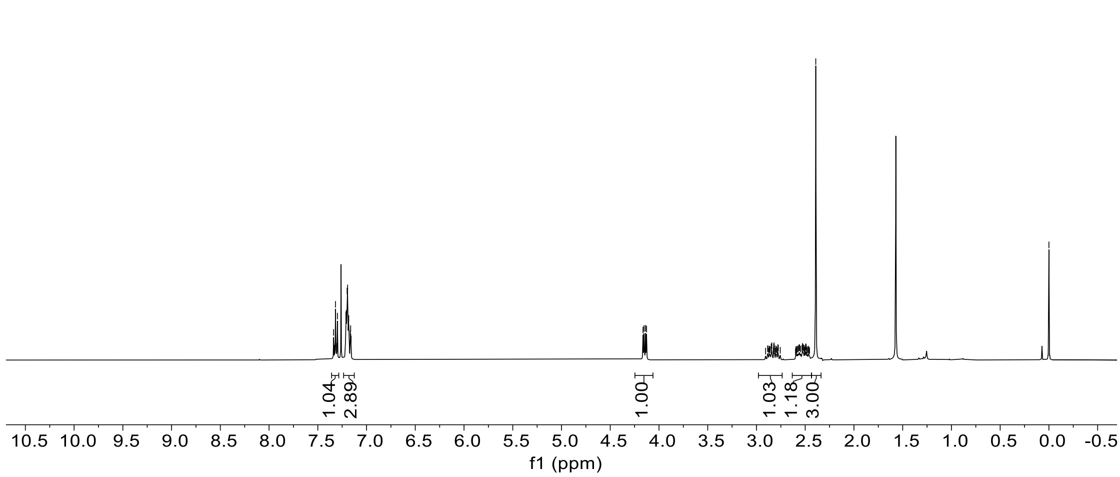

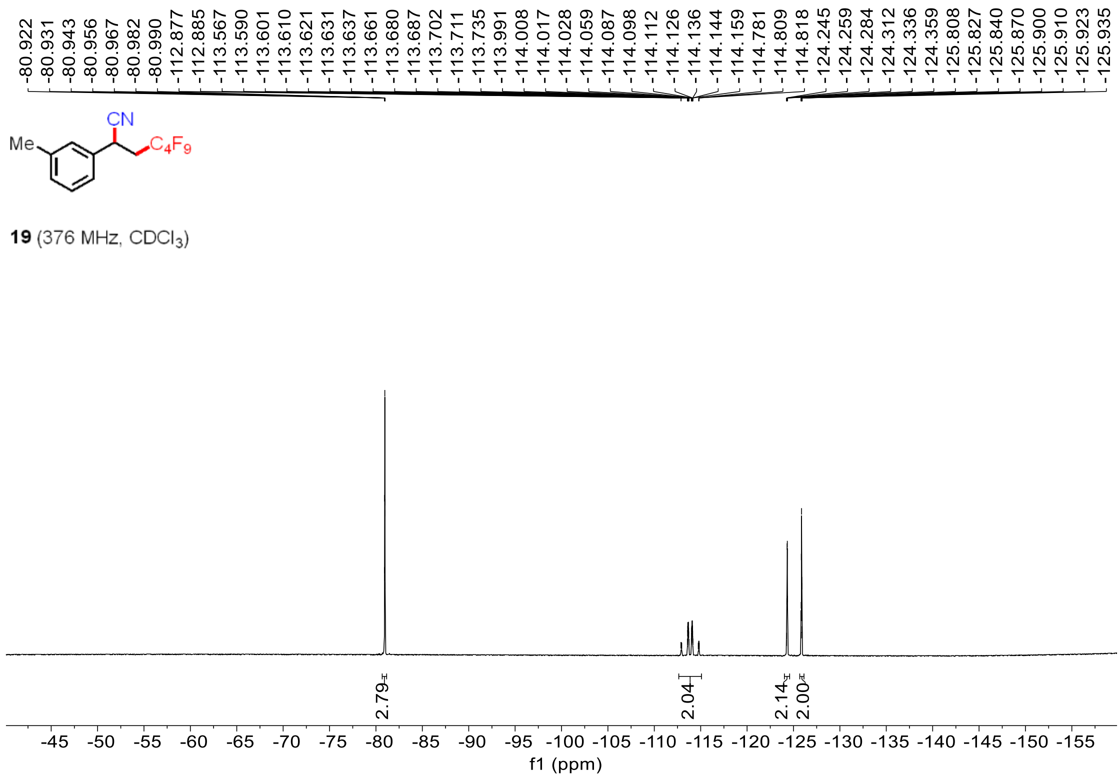

19 (376 MHz, $CDCl_3$)

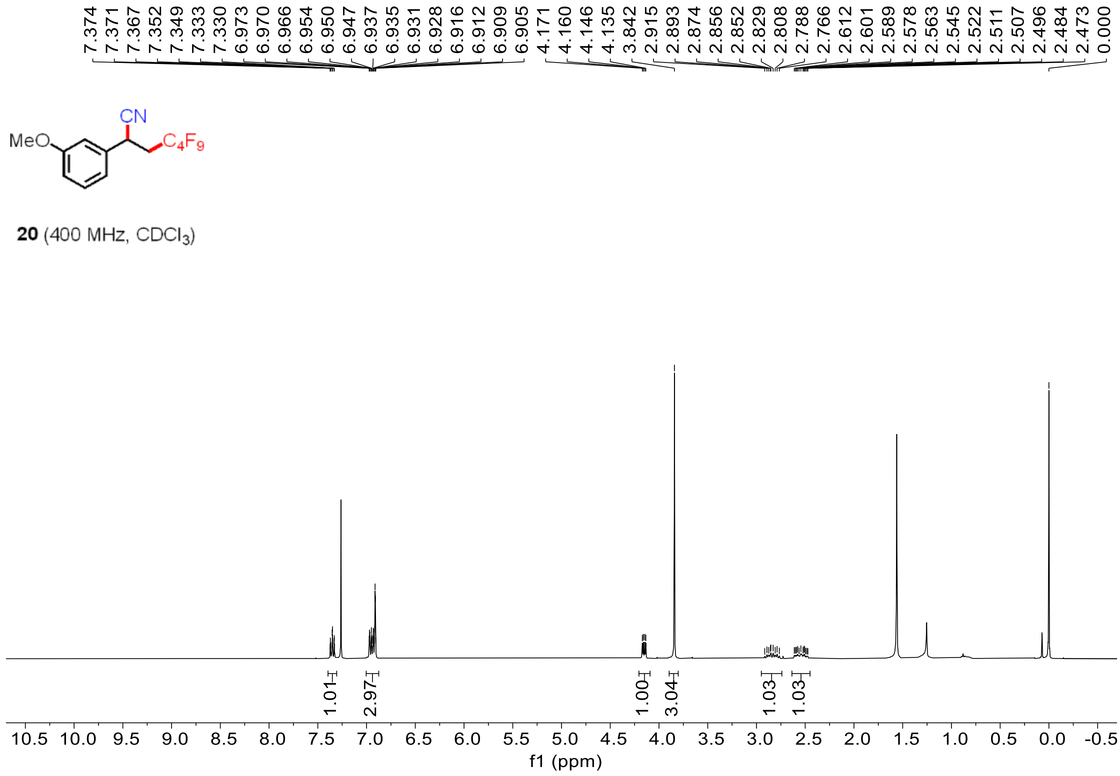

20 (400 MHz, $CDCl_3$)

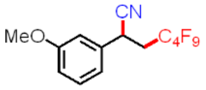

20 (376 MHz, CDCl$_3$)

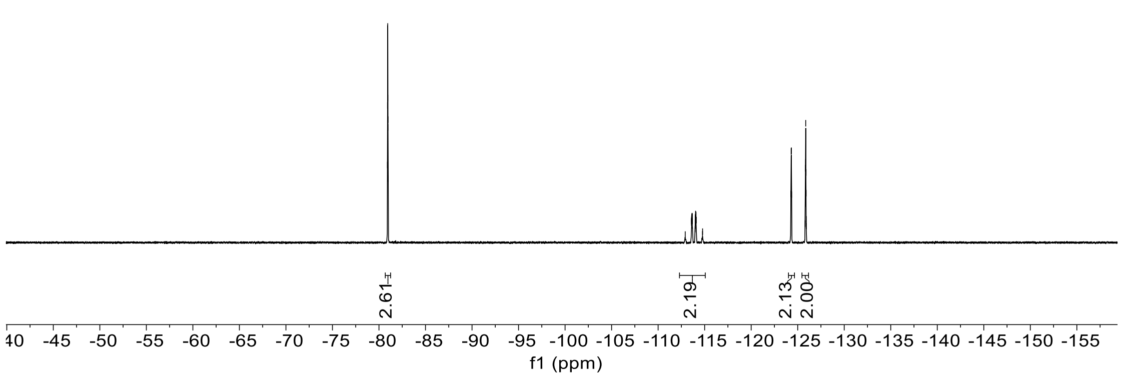

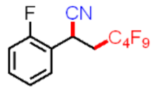

21 (400 MHz, CDCl$_3$)

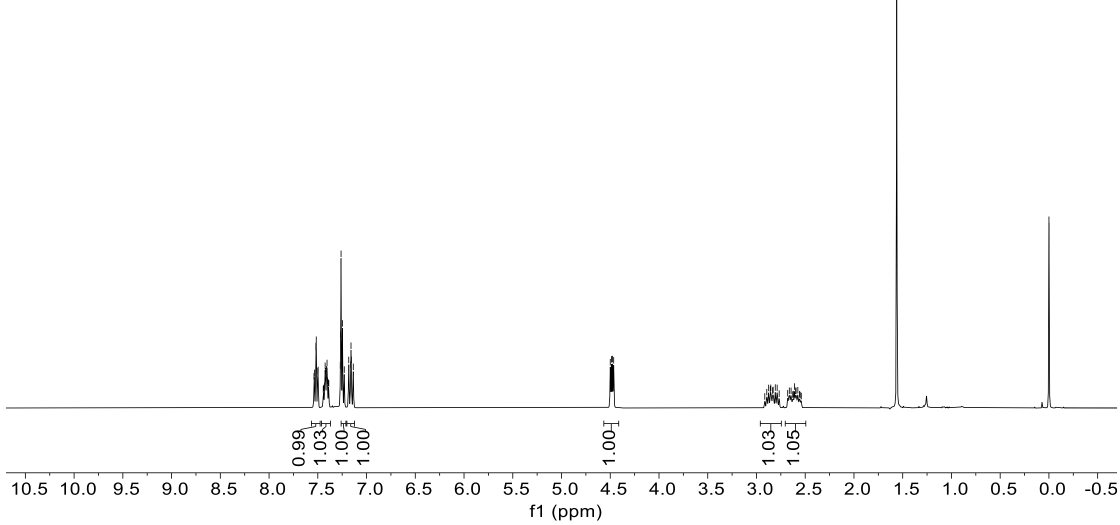

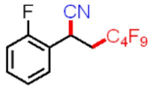

21 (376 MHz, $CDCl_3$)

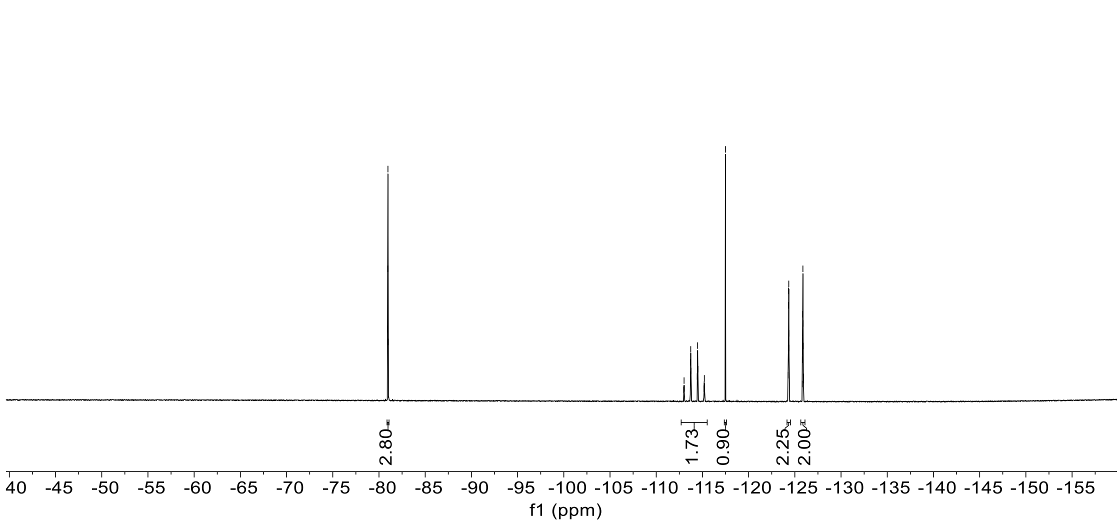

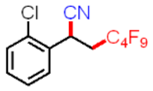

22 (400 MHz, $CDCl_3$)

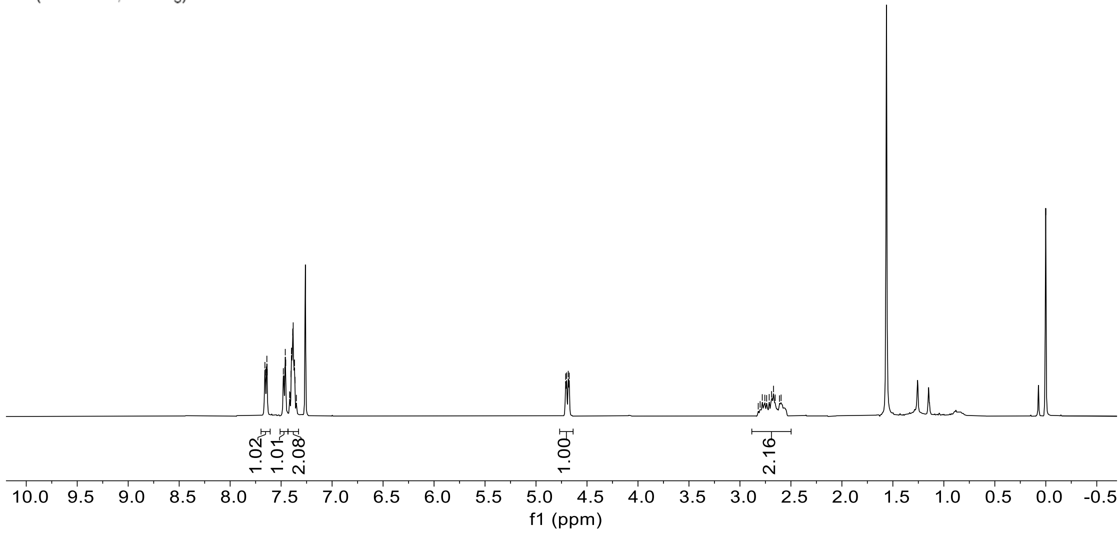

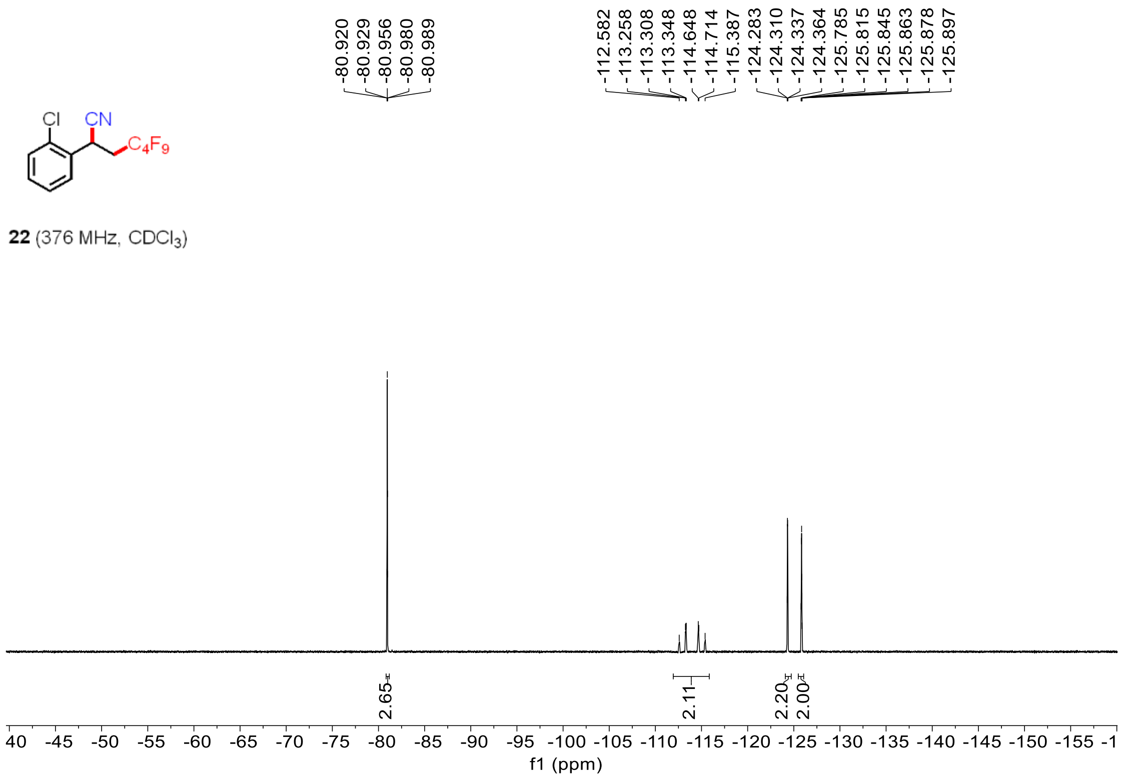

22 (376 MHz, $CDCl_3$)

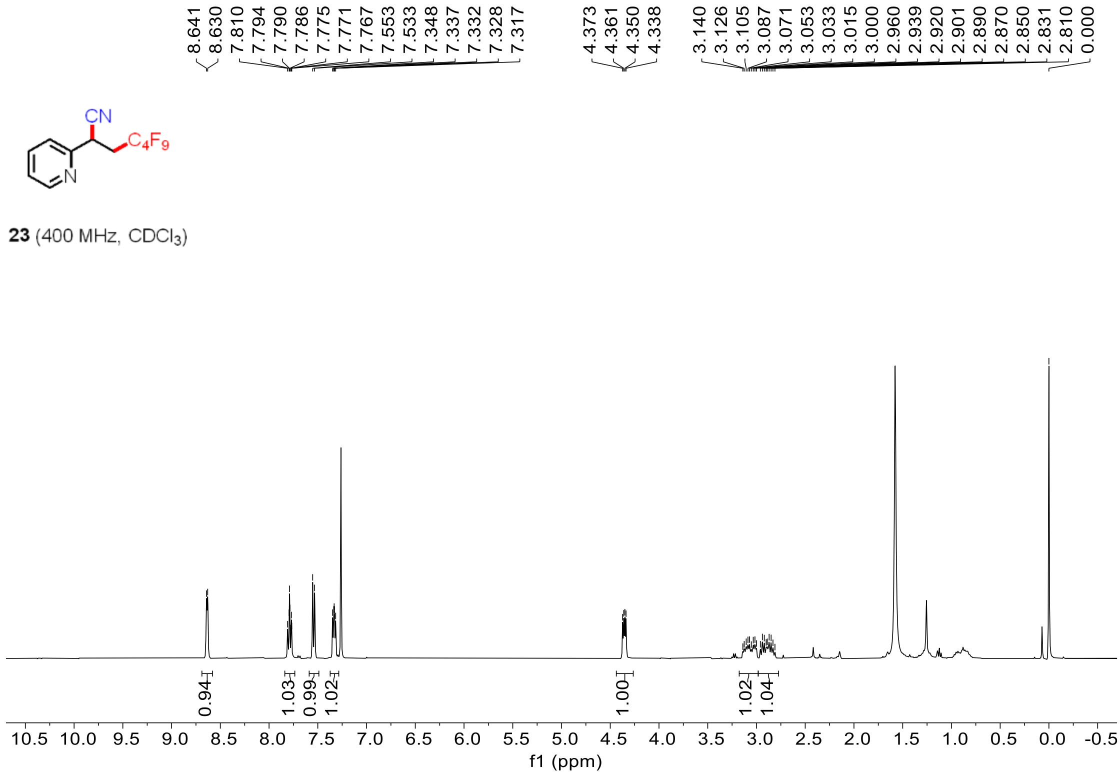

23 (400 MHz, $CDCl_3$)

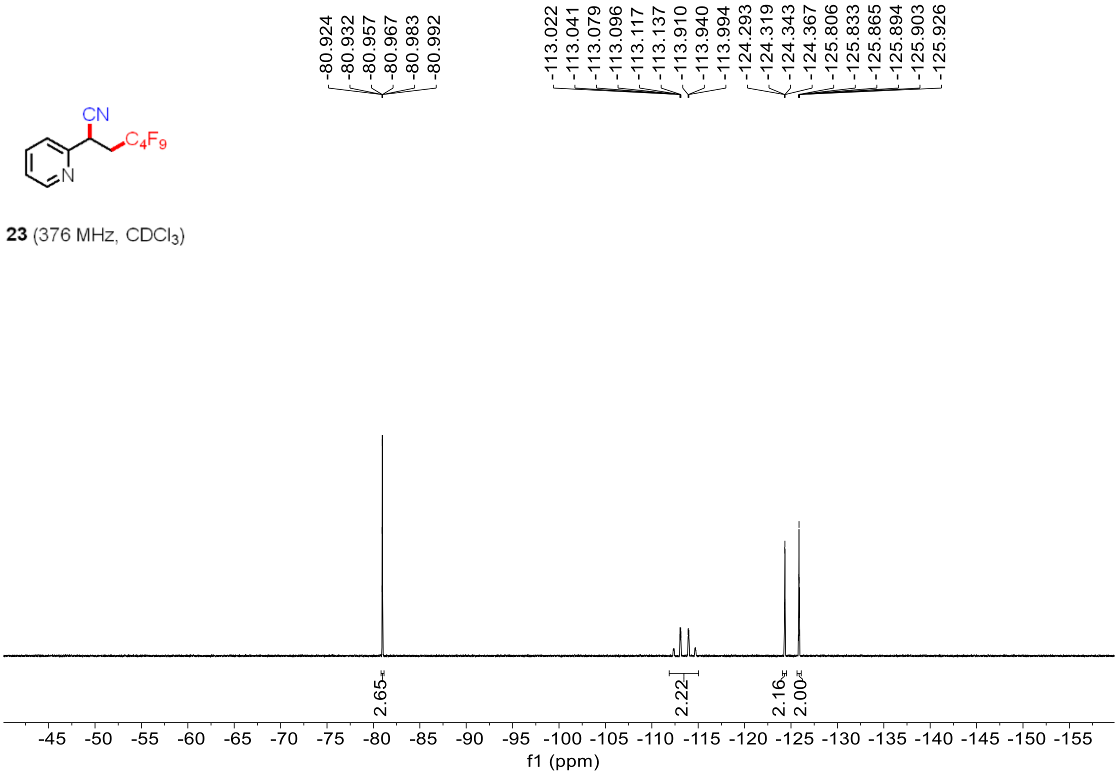

23 (376 MHz, $CDCl_3$)

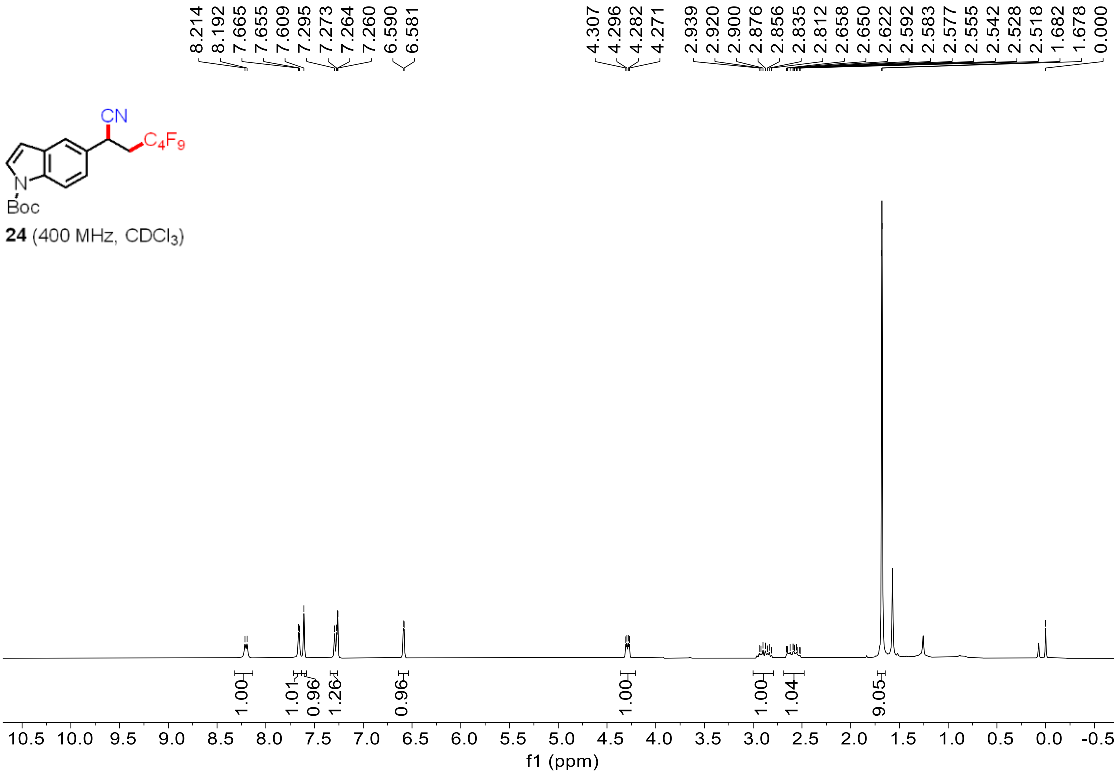

24 (400 MHz, $CDCl_3$)

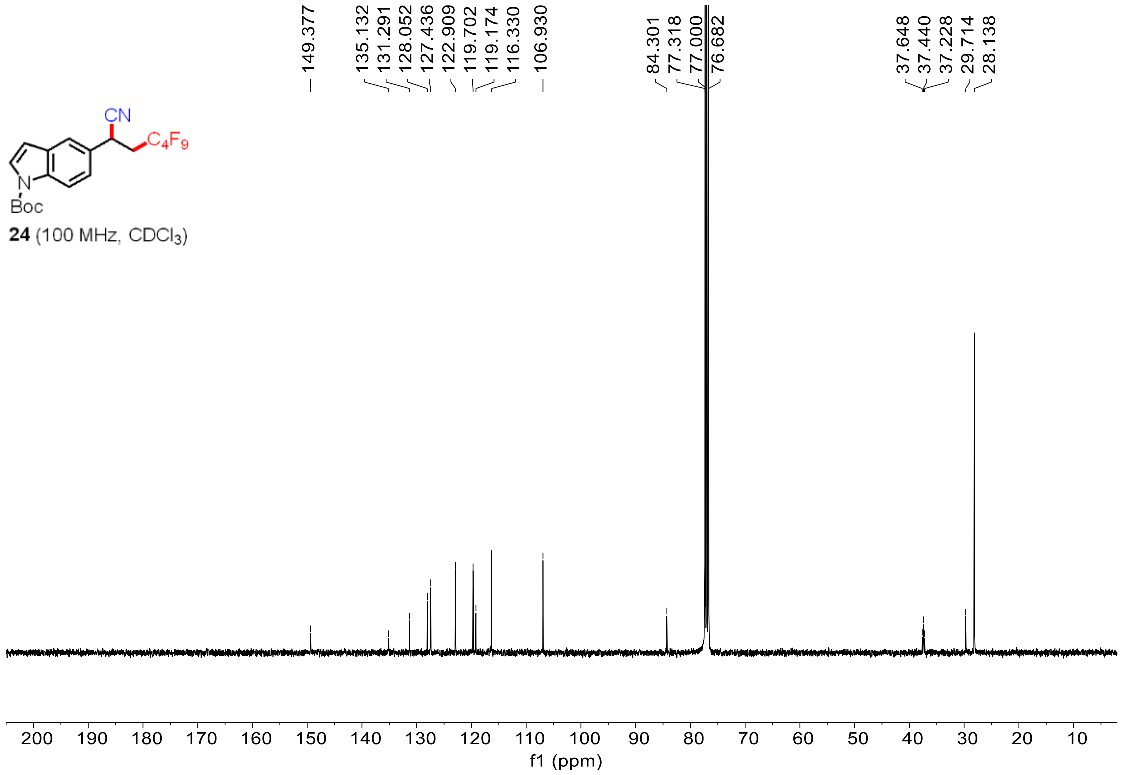

24 (100 MHz, $CDCl_3$)

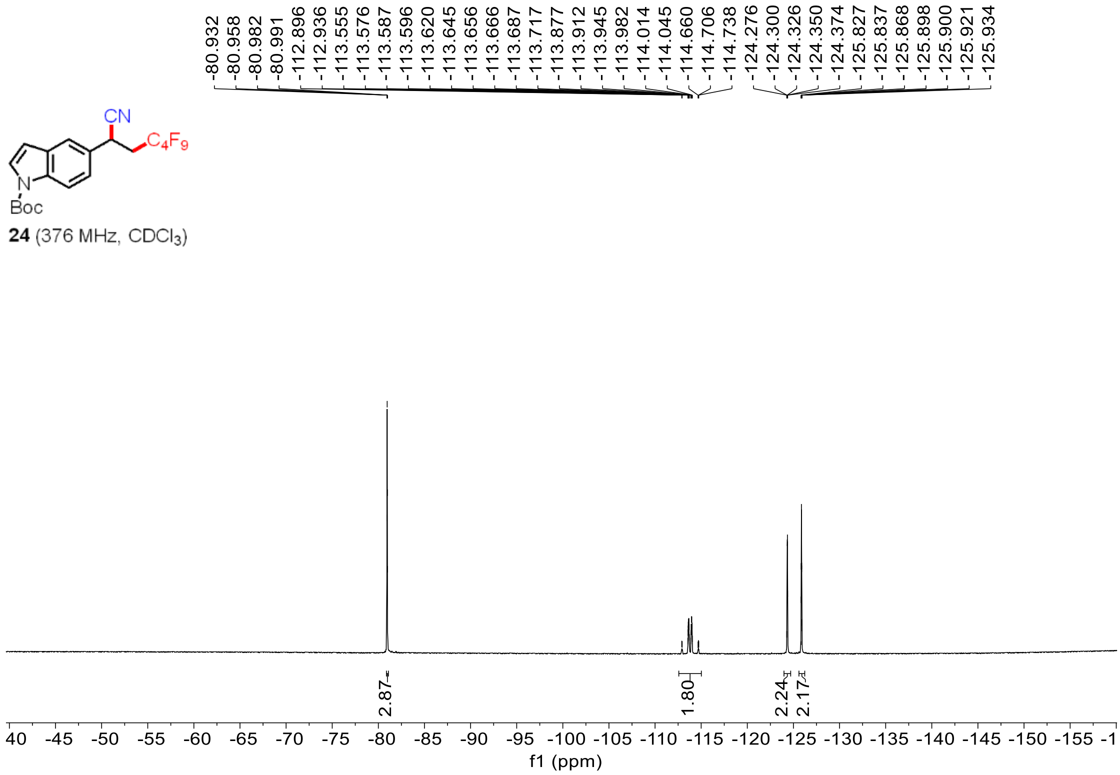

24 (376 MHz, $CDCl_3$)

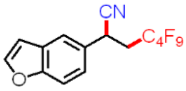

25 (400 MHz, $CDCl_3$)

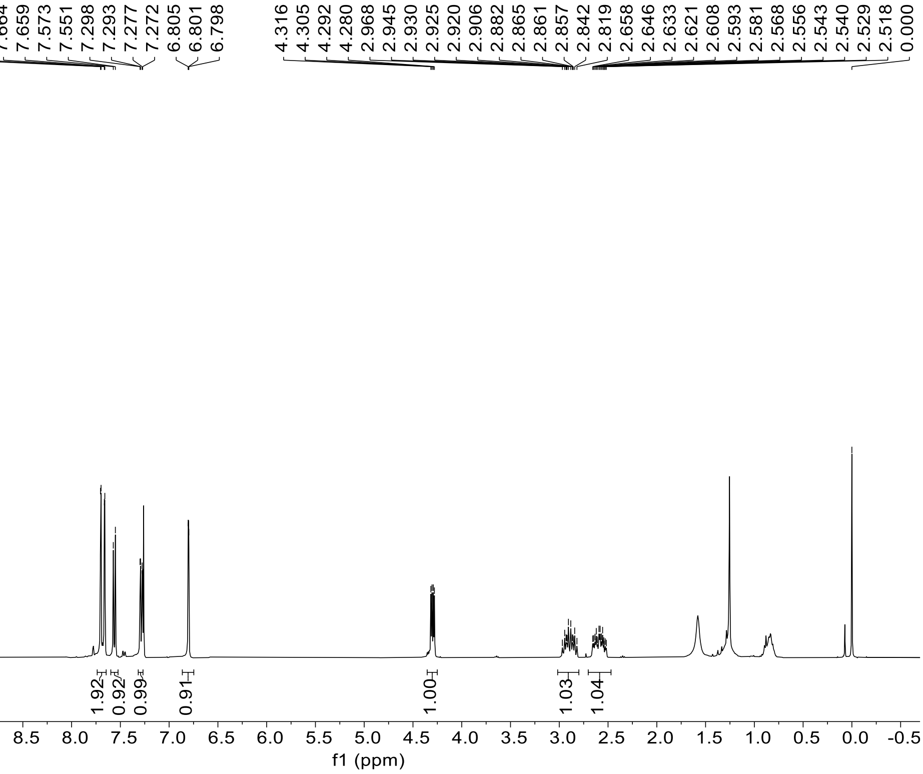

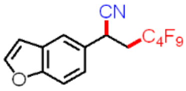

25 (100 MHz, $CDCl_3$)

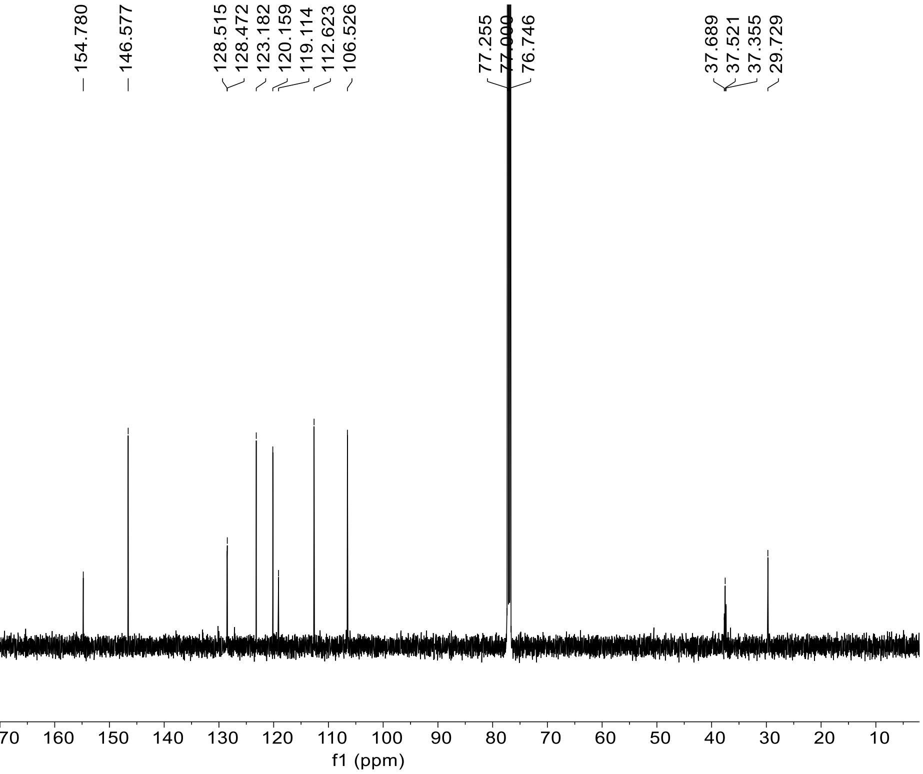

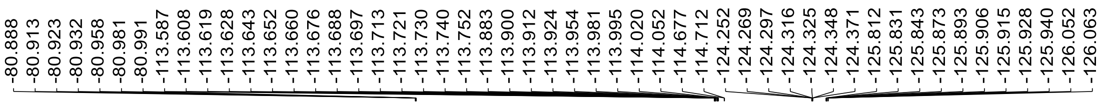

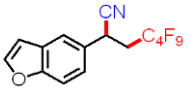

25 (376 MHz, $CDCl_3$)

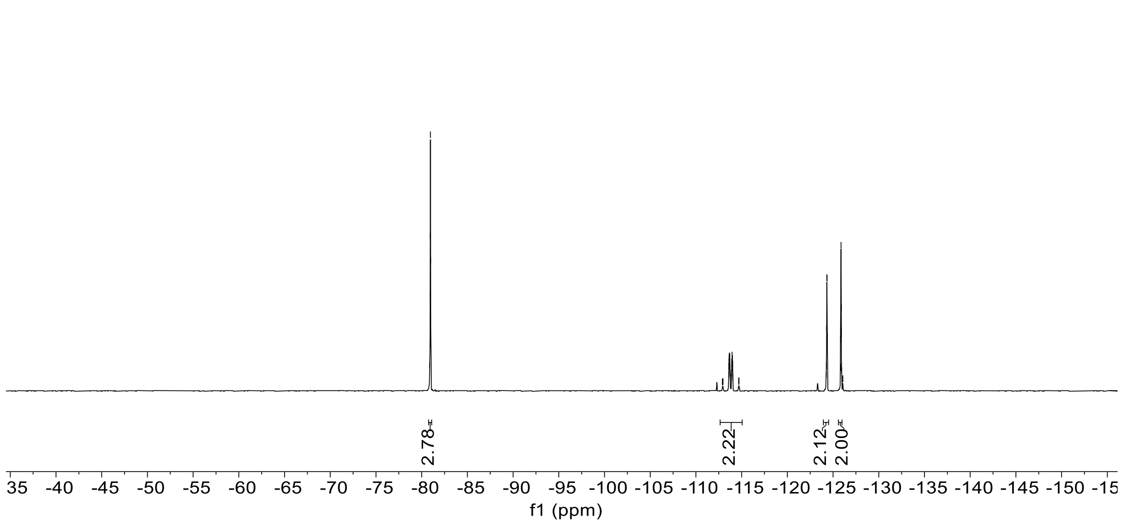

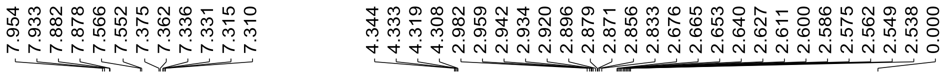

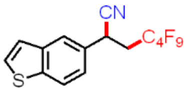

26 (400 MHz, $CDCl_3$)

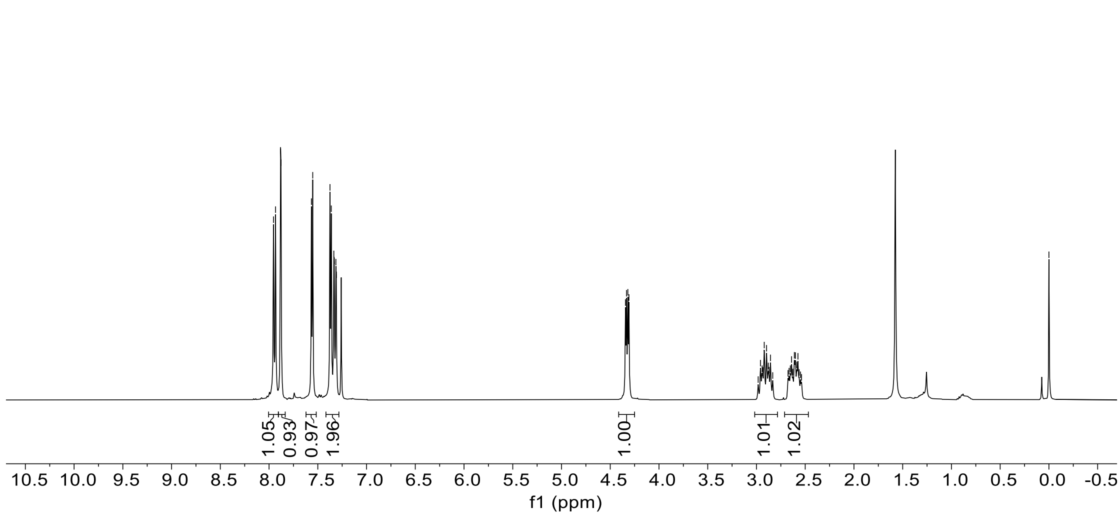

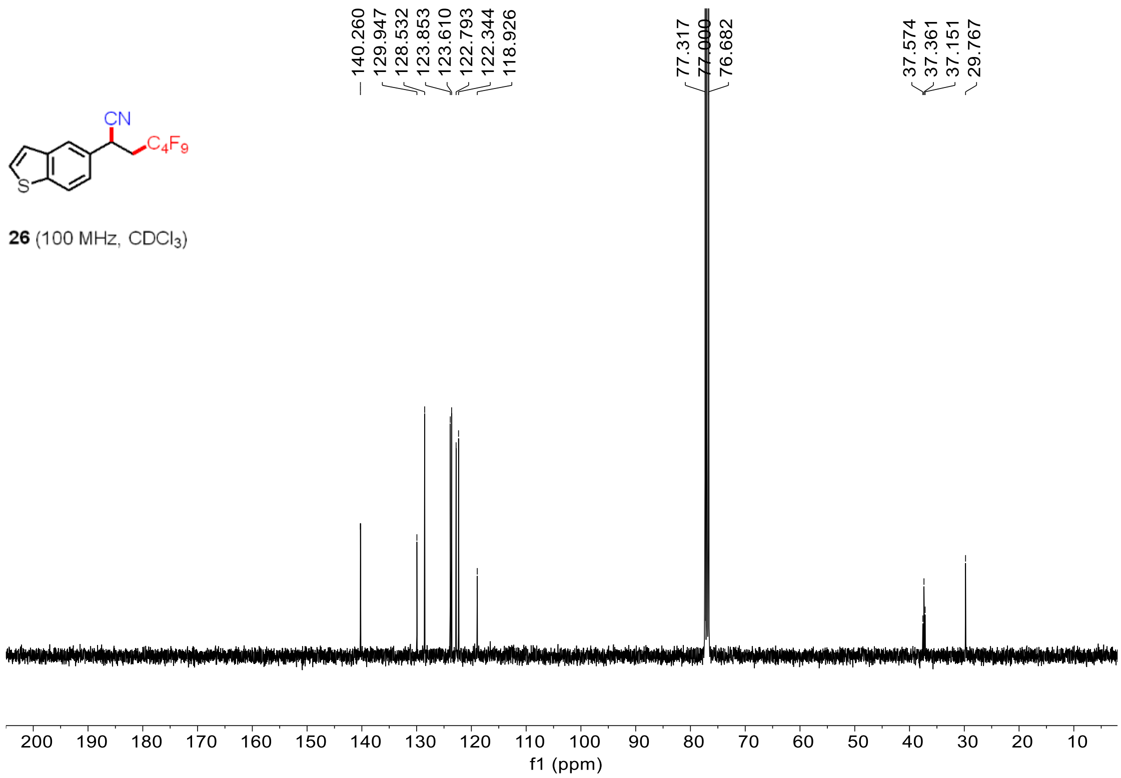
140.260
129.947
128.532
123.853
123.610
122.793
122.344
118.926
77.317
77.000
76.682
37.574
37.361
37.151
29.767
CN
C4F9
S
26 (100 MHz, CDCl3)
200 190 180 170 160 150 140 130 120 110 100 90 80 70 60 50 40 30 20 10
f1 (ppm)

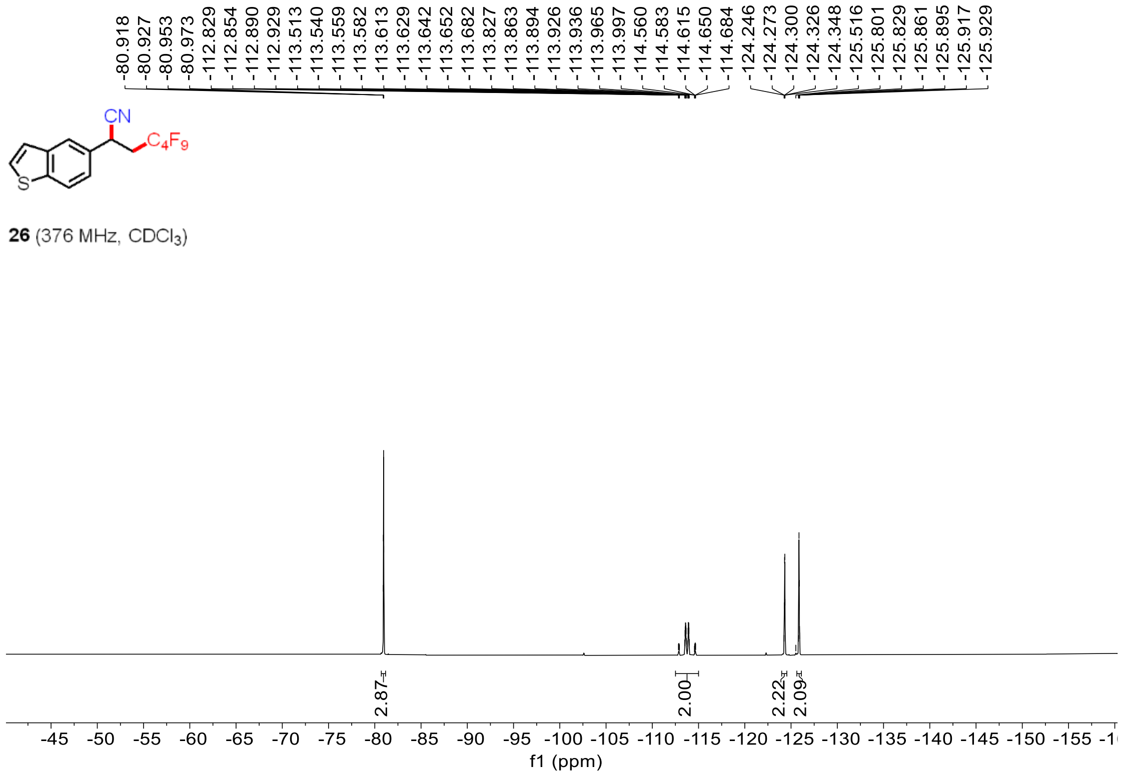
-80.918
-80.927
-80.953
-80.973
-112.829
-112.854
-112.890
-112.929
-113.513
-113.540
-113.559
-113.582
-113.613
-113.629
-113.642
-113.652
-113.682
-113.827
-113.863
-113.894
-113.926
-113.936
-113.965
-113.997
-114.560
-114.583
-114.615
-114.650
-114.684
-124.246
-124.273
-124.300
-124.326
-124.348
-125.516
-125.801
-125.829
-125.861
-125.895
-125.917
-125.929
CN
C4F9
S
26 (376 MHz, CDCl3)
2.87
2.00
2.22
2.09
-45 -50 -55 -60 -65 -70 -75 -80 -85 -90 -95 -100 -105 -110 -115 -120 -125 -130 -135 -140 -145 -150 -155
f1 (ppm)

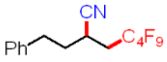

27 (400 MHz, $CDCl_3$)

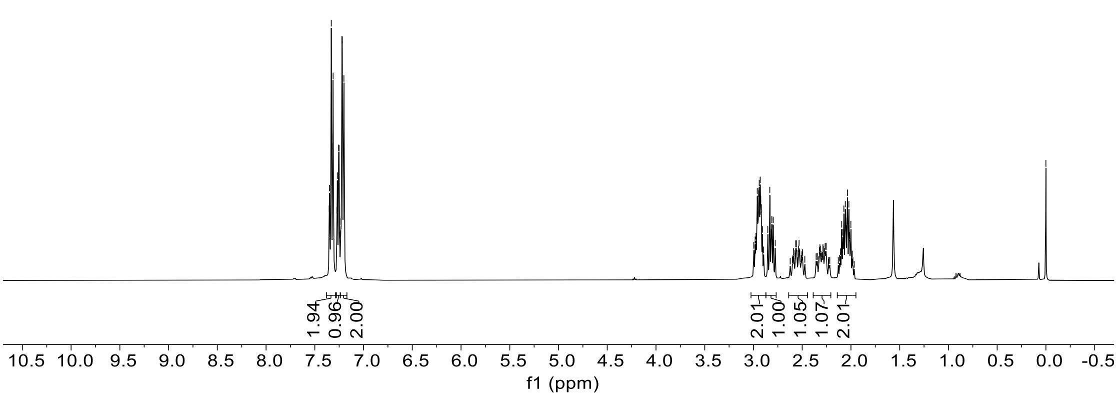

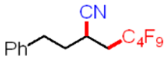

27 (376 MHz, $CDCl_3$)

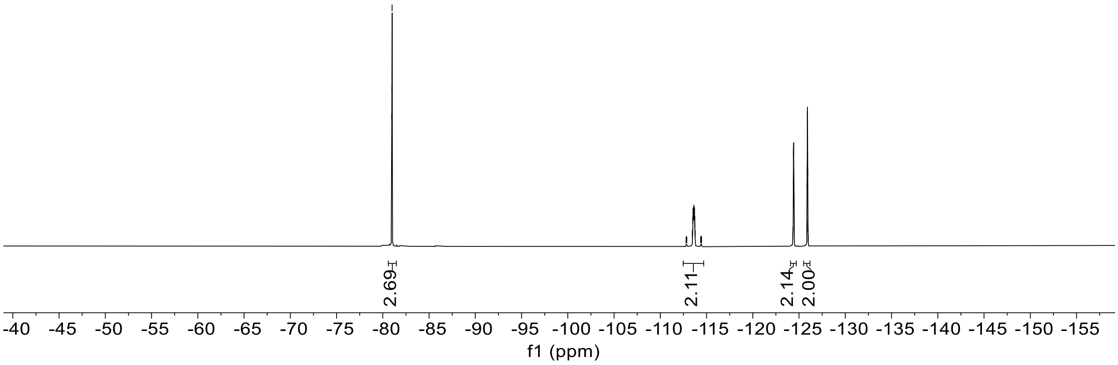

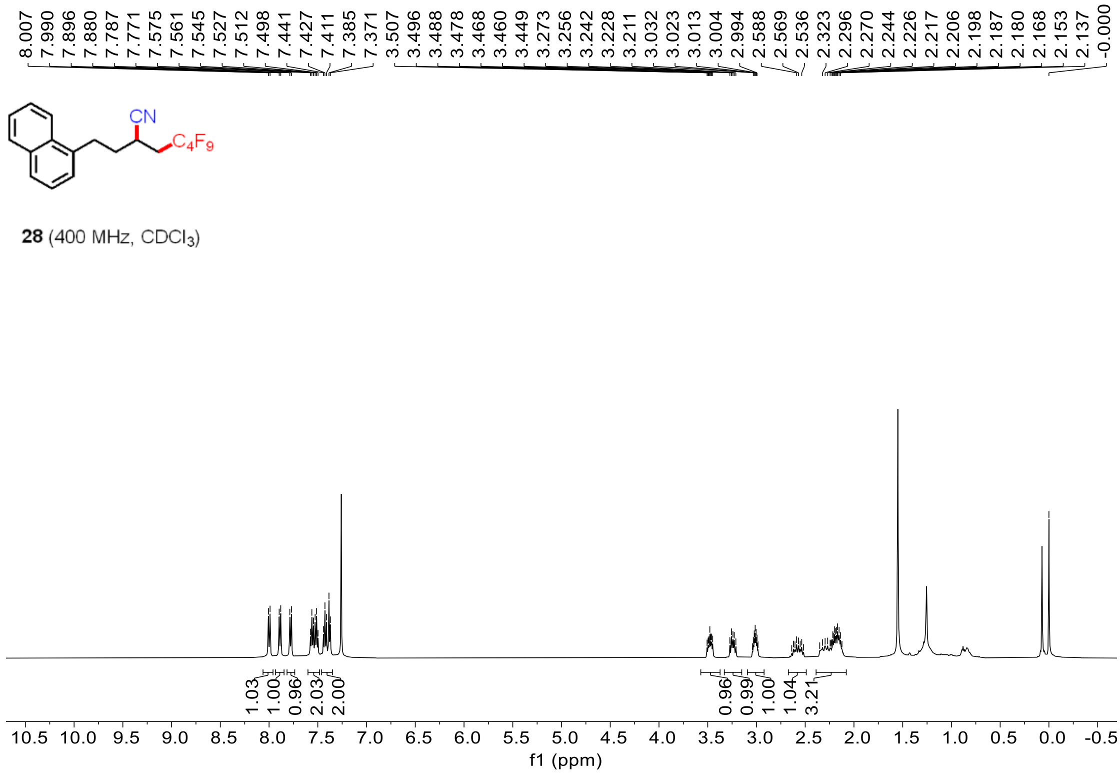

28 (400 MHz, $CDCl_3$)

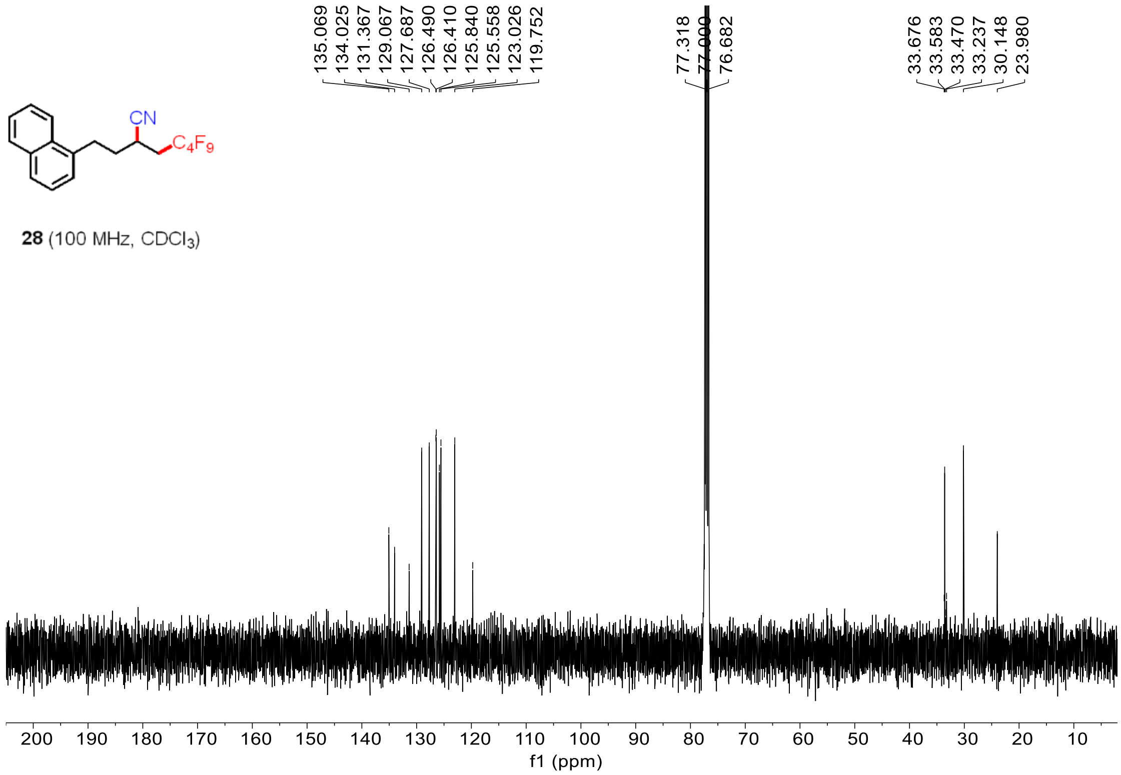

28 (100 MHz, $CDCl_3$)

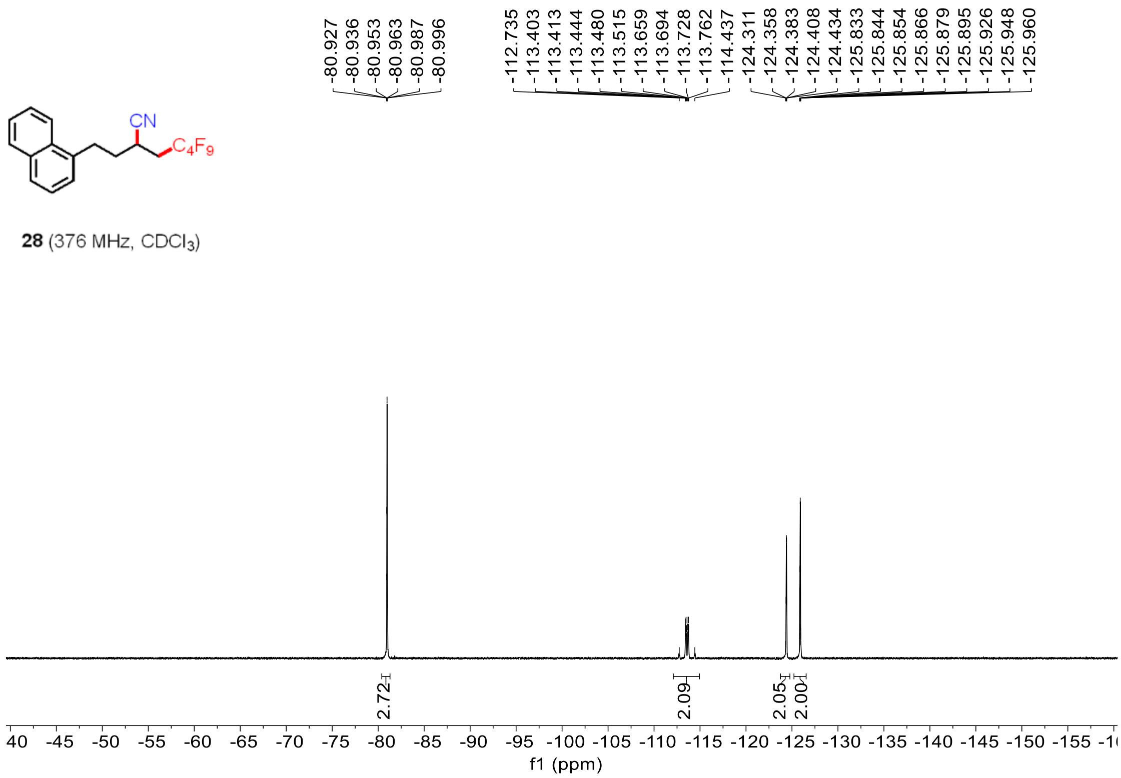

28 (376 MHz, $CDCl_3$)

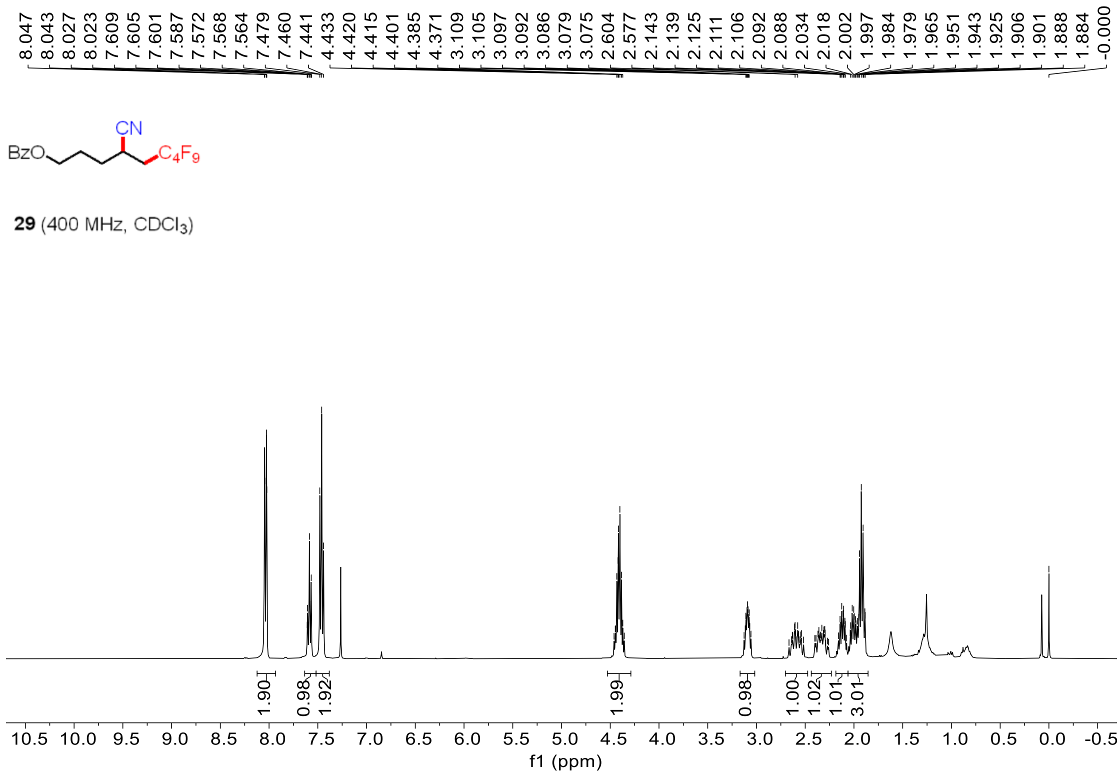

29 (400 MHz, $CDCl_3$)

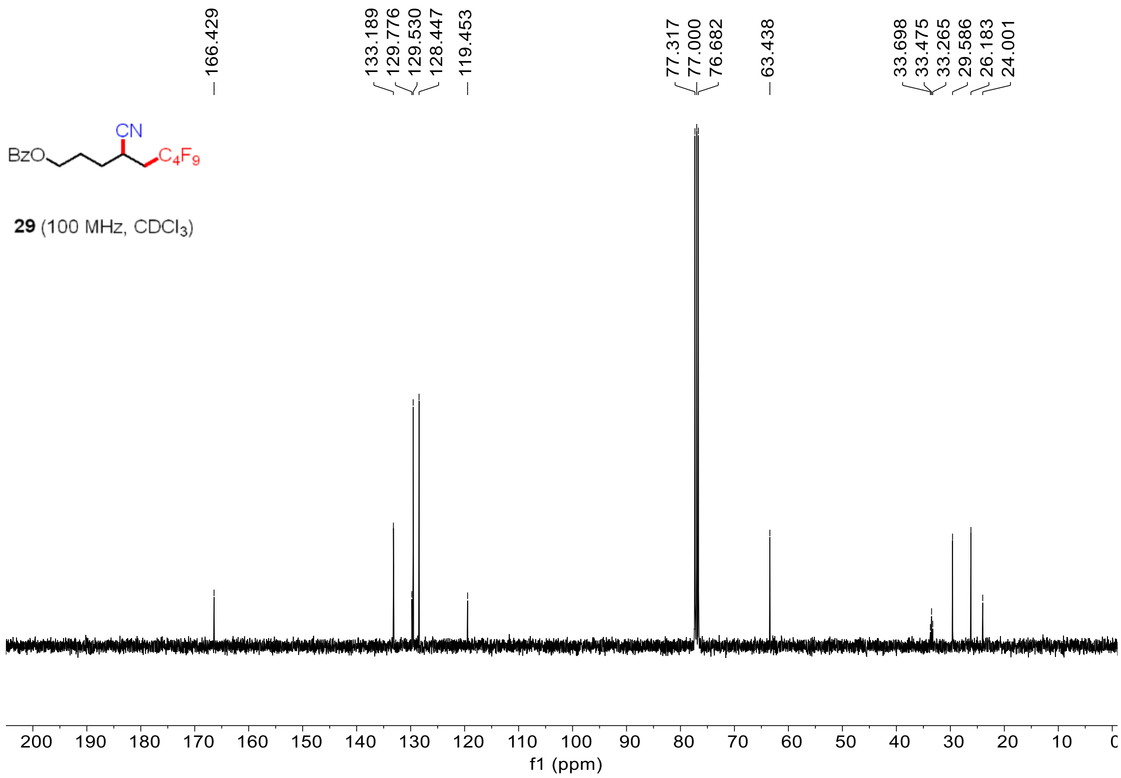
166.429
133.189
129.776
129.530
128.447
119.453
77.317
77.000
76.682
63.438
33.698
33.475
33.265
29.586
26.183
24.001
BzO
CN
C4F9
29 (100 MHz, CDCl3)
f1 (ppm)

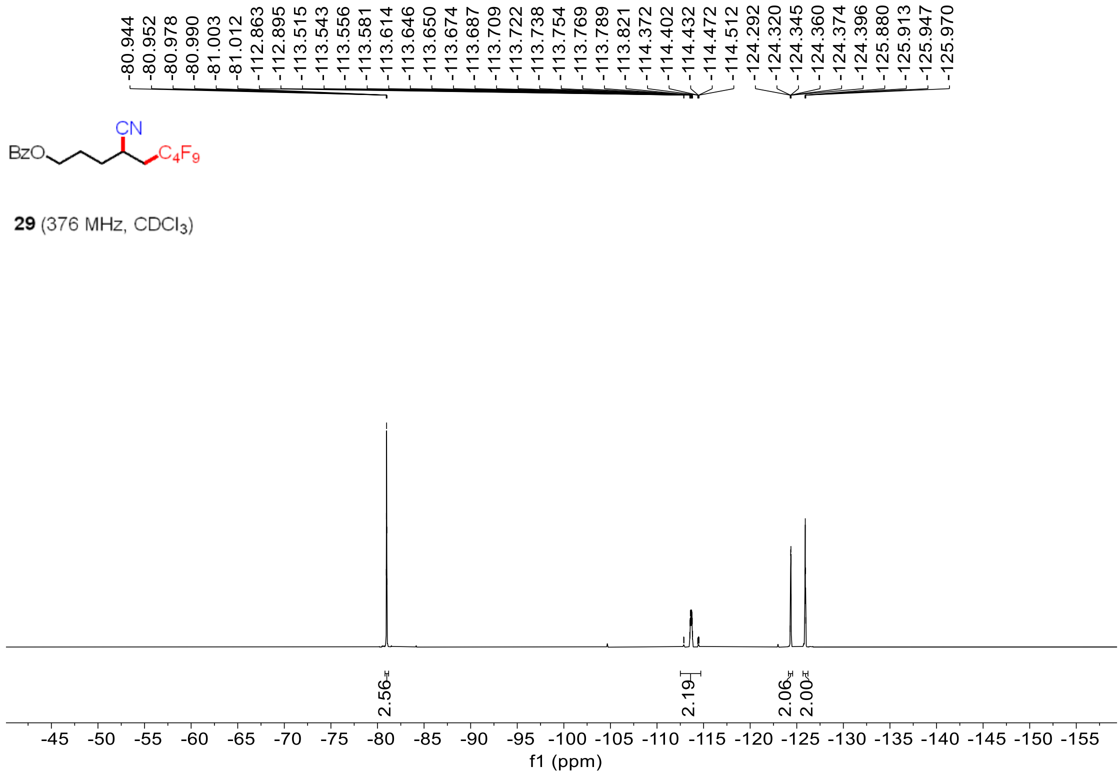
-80.944
-80.952
-80.978
-80.990
-81.003
-81.012
-112.863
-112.895
-113.515
-113.543
-113.556
-113.581
-113.614
-113.646
-113.650
-113.674
-113.687
-113.709
-113.722
-113.738
-113.754
-113.769
-113.789
-113.821
-114.372
-114.402
-114.432
-114.472
-114.512
-124.292
-124.320
-124.345
-124.360
-124.374
-124.396
-125.880
-125.913
-125.947
-125.970
BzO
CN
C4F9
29 (376 MHz, CDCl3)
2.56
2.19
2.06
2.00
f1 (ppm)

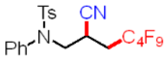

30 (400 MHz, $CDCl_3$)

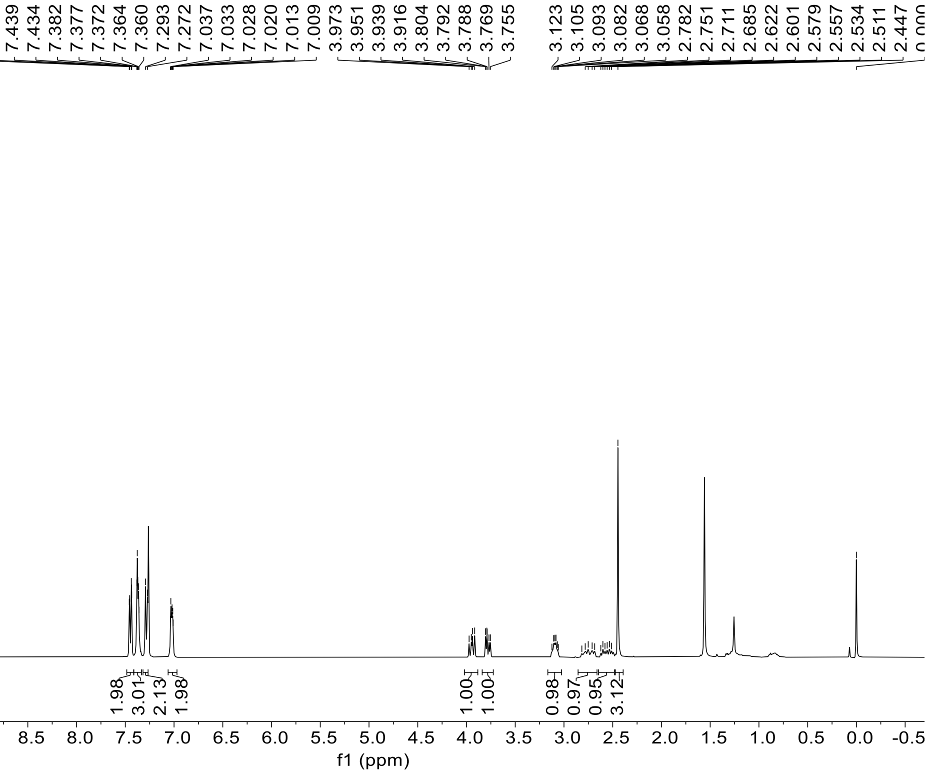

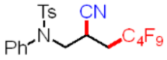

30 (100 MHz, $CDCl_3$)

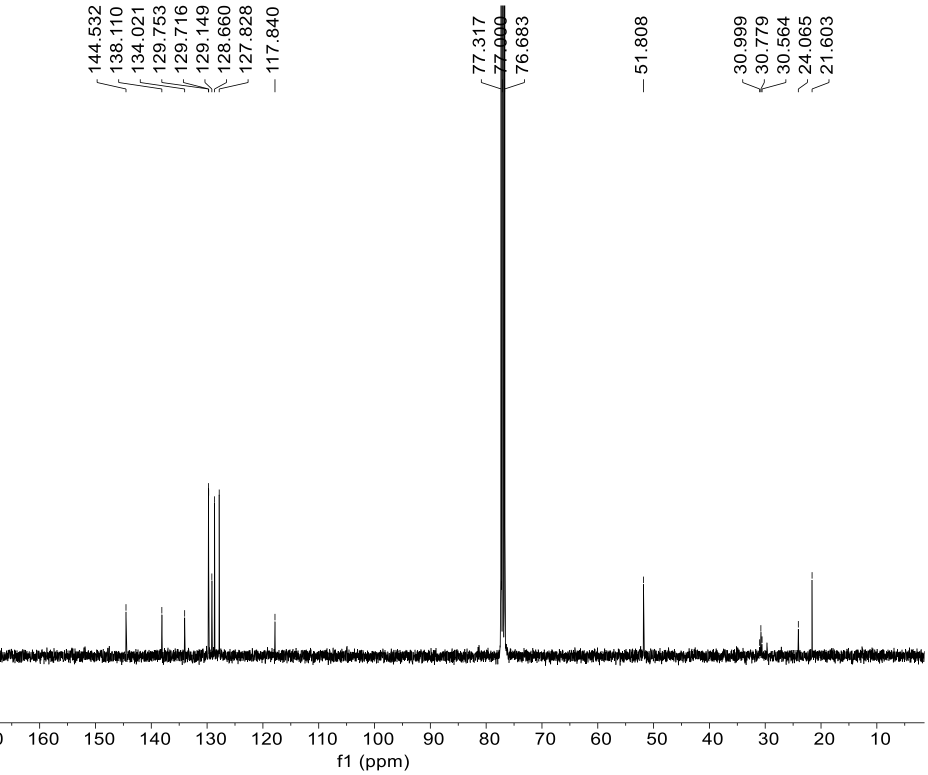

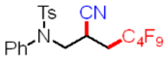

30 (376 MHz, $CDCl_3$)

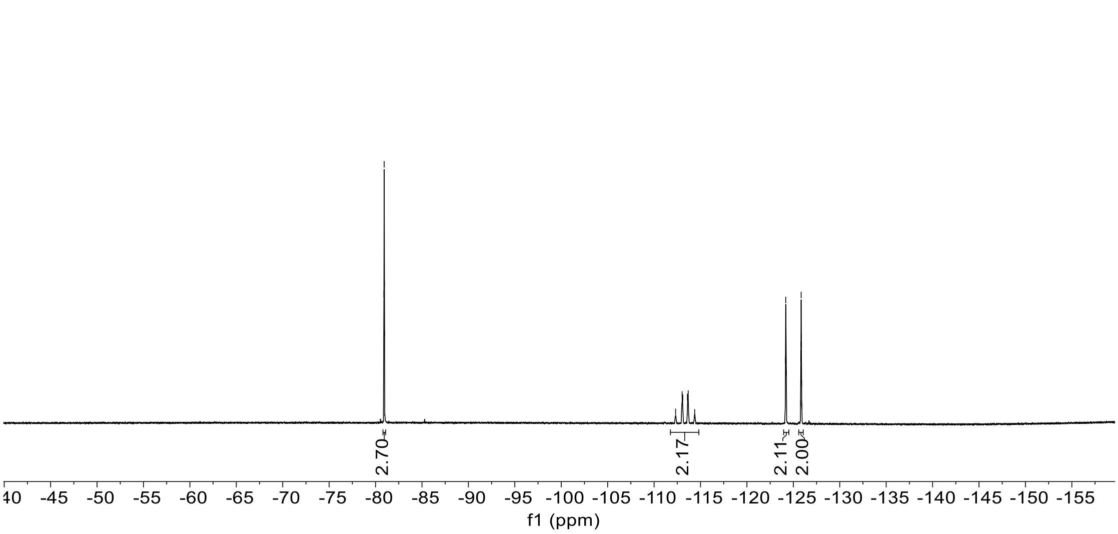

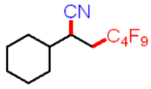

31 (400 MHz, $CDCl_3$)

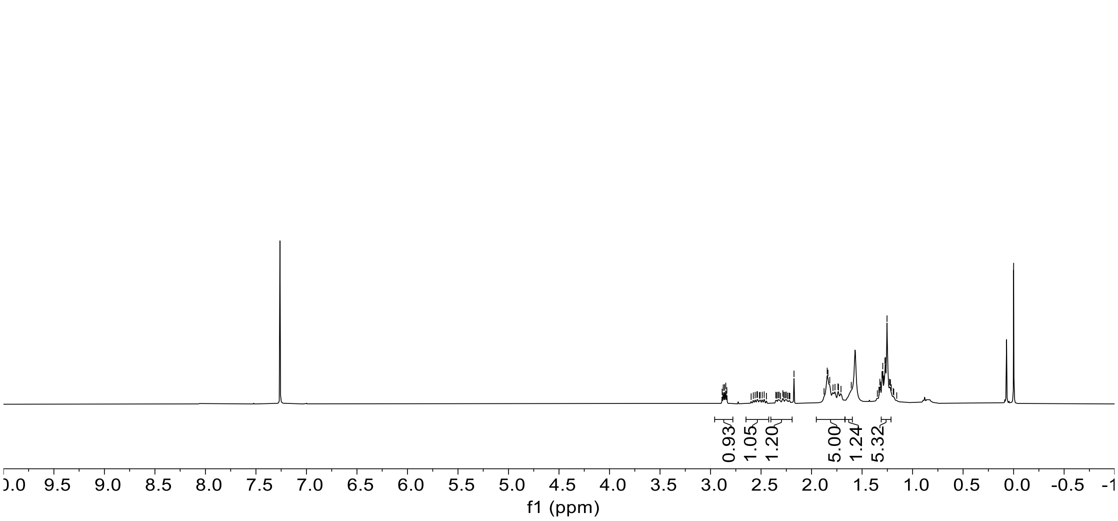

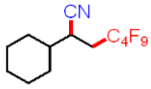

31 (376 MHz, $CDCl_3$)

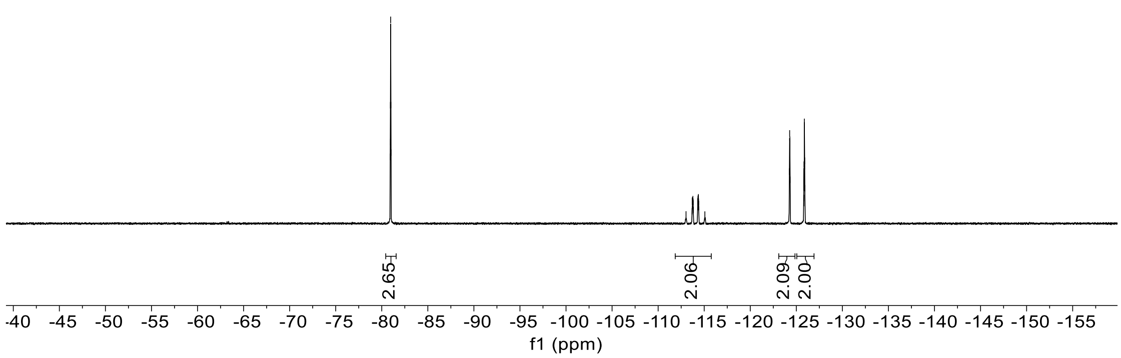

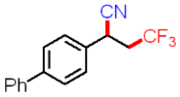

32 (400 MHz, $CDCl_3$)

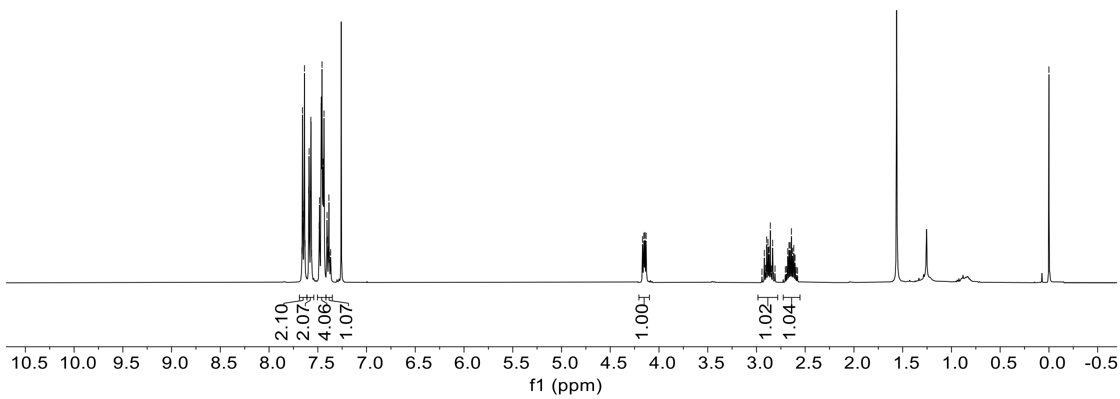

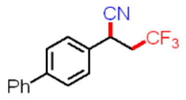

32 (376 MHz, $CDCl_3$)

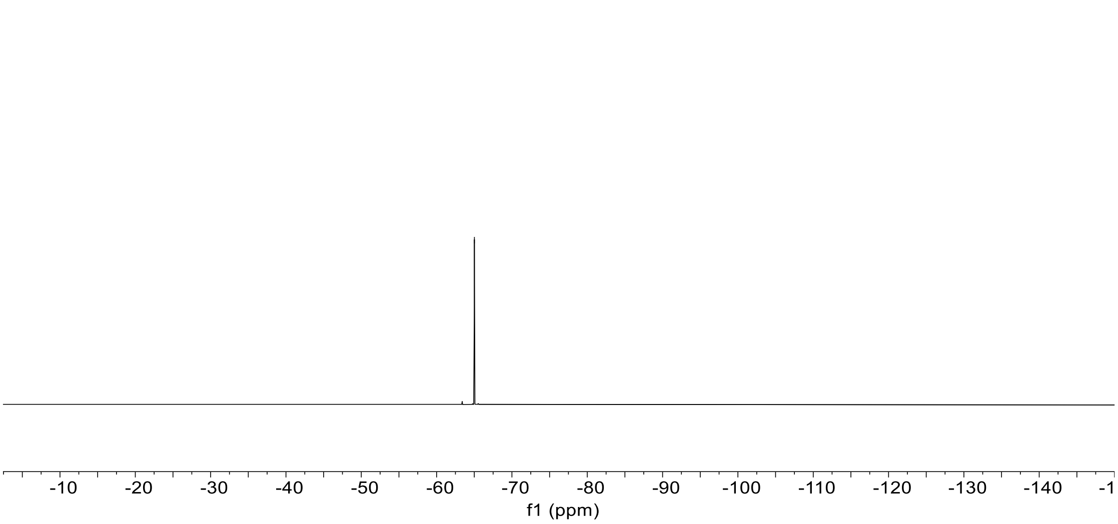

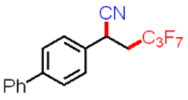

33 (400 MHz, $CDCl_3$)

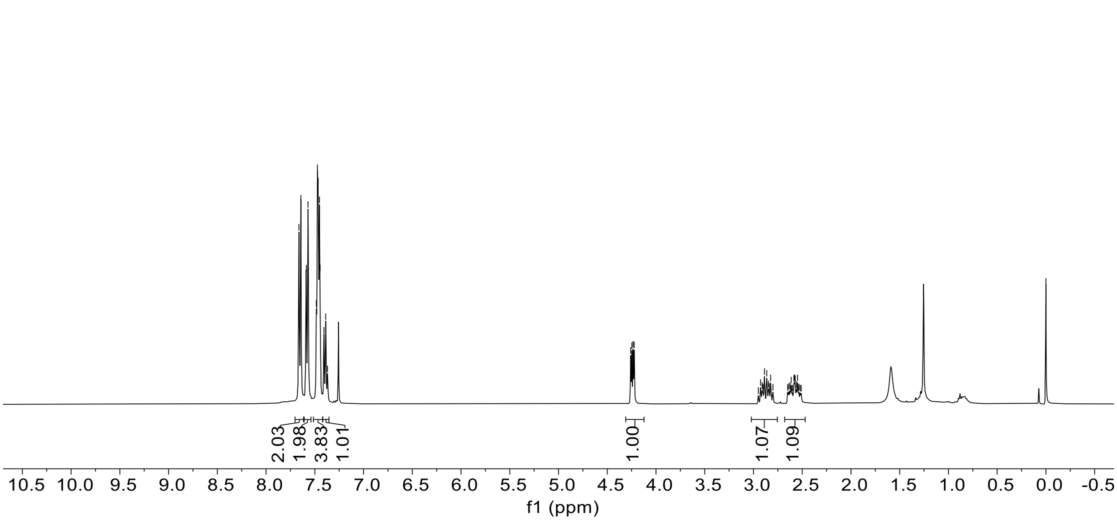

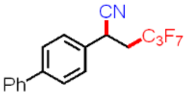

33 (100 MHz, $CDCl_3$)

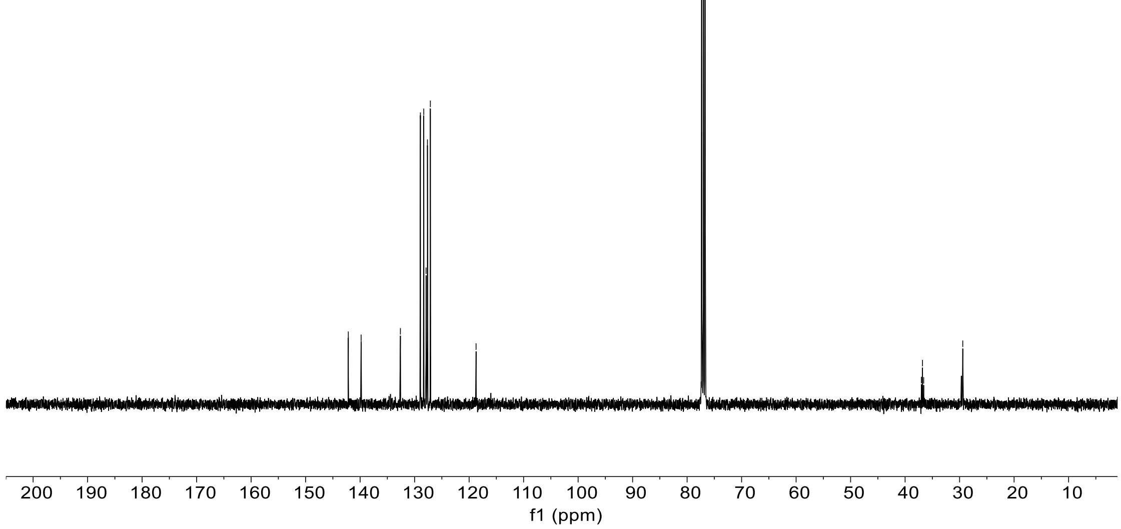

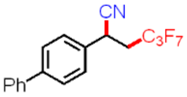

33 (376 MHz, $CDCl_3$)

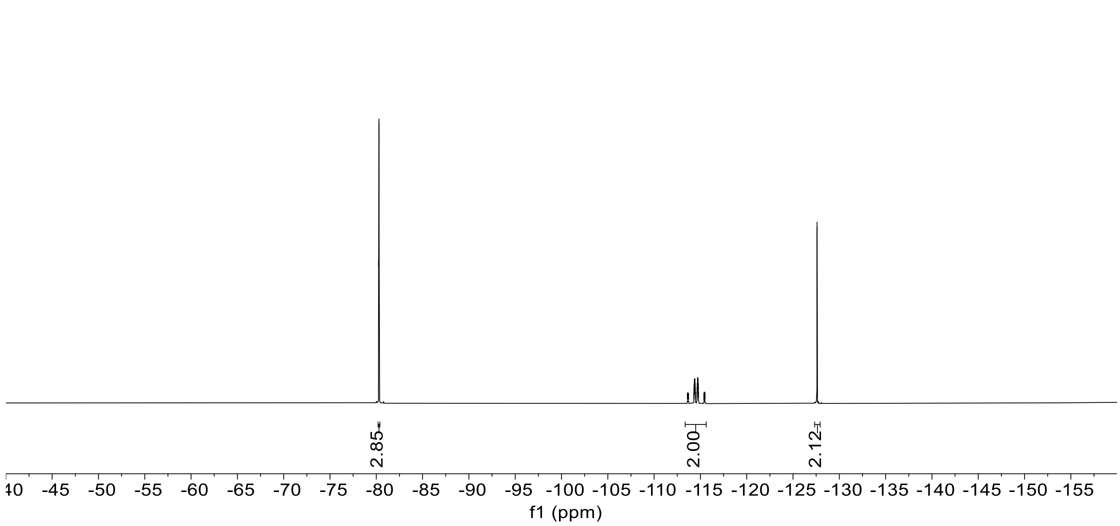

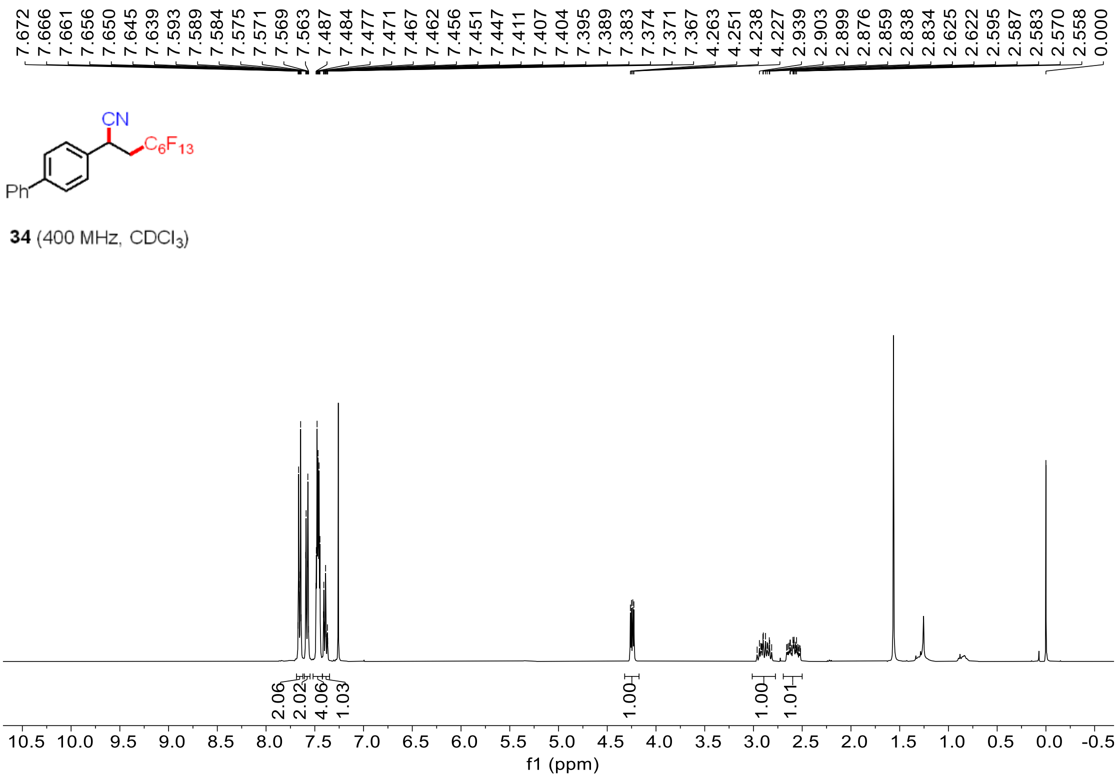

34 (400 MHz, $CDCl_3$)

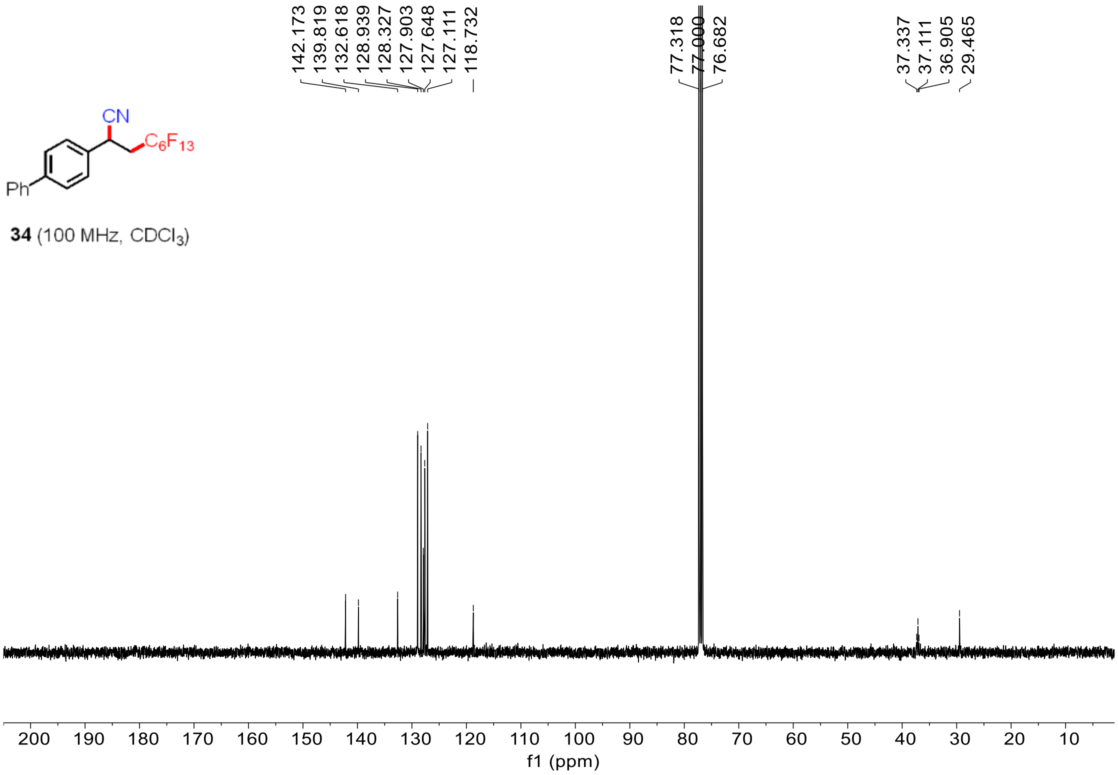

34 (100 MHz, $CDCl_3$)

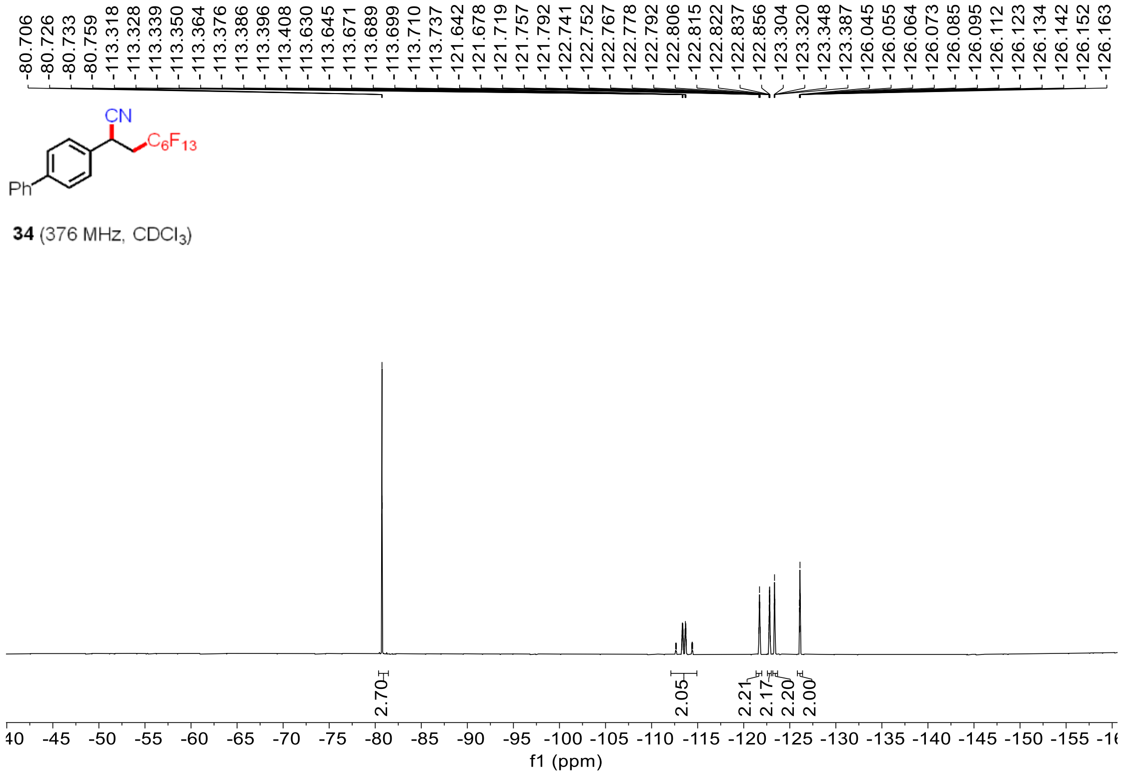

34 (376 MHz, $CDCl_3$)

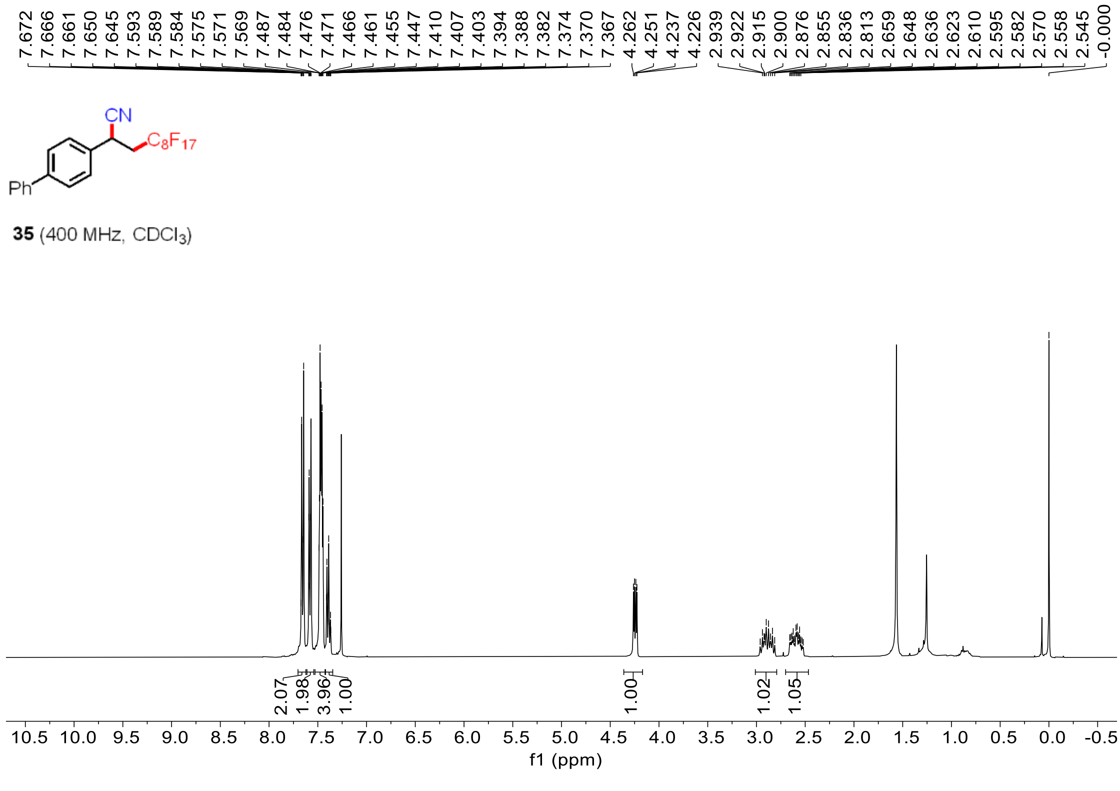

35 (400 MHz, $CDCl_3$)

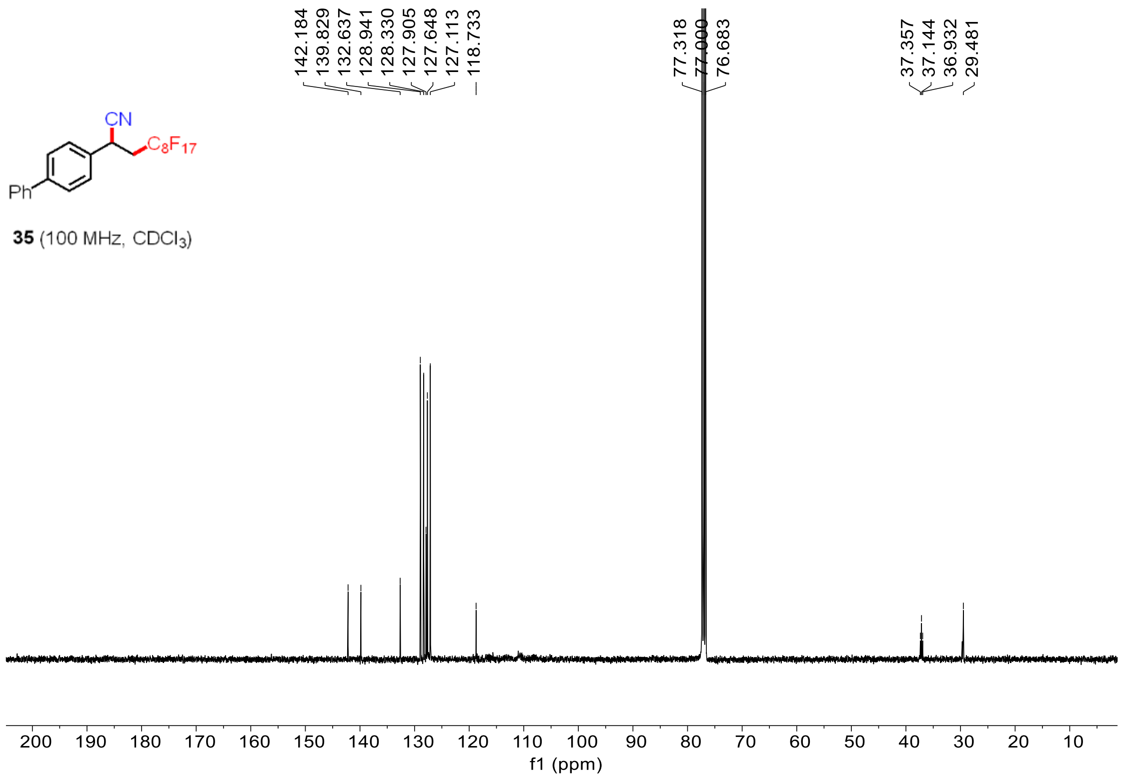

35 (100 MHz, $CDCl_3$)

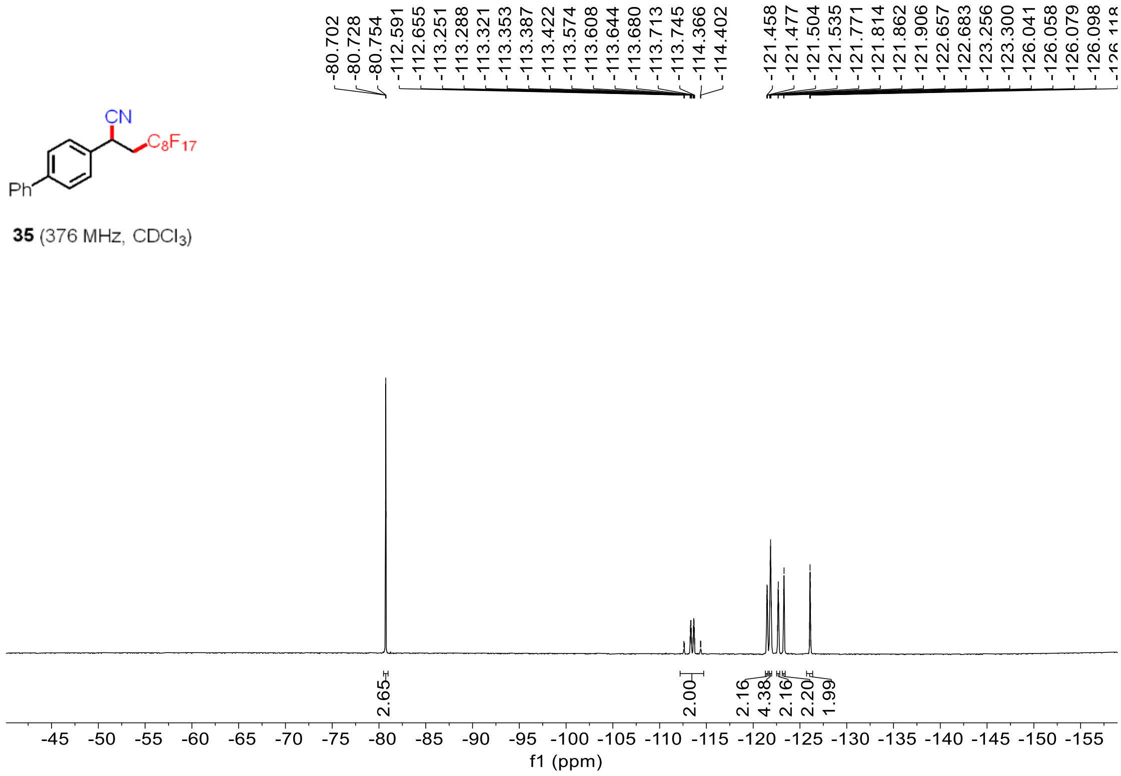

35 (376 MHz, $CDCl_3$)

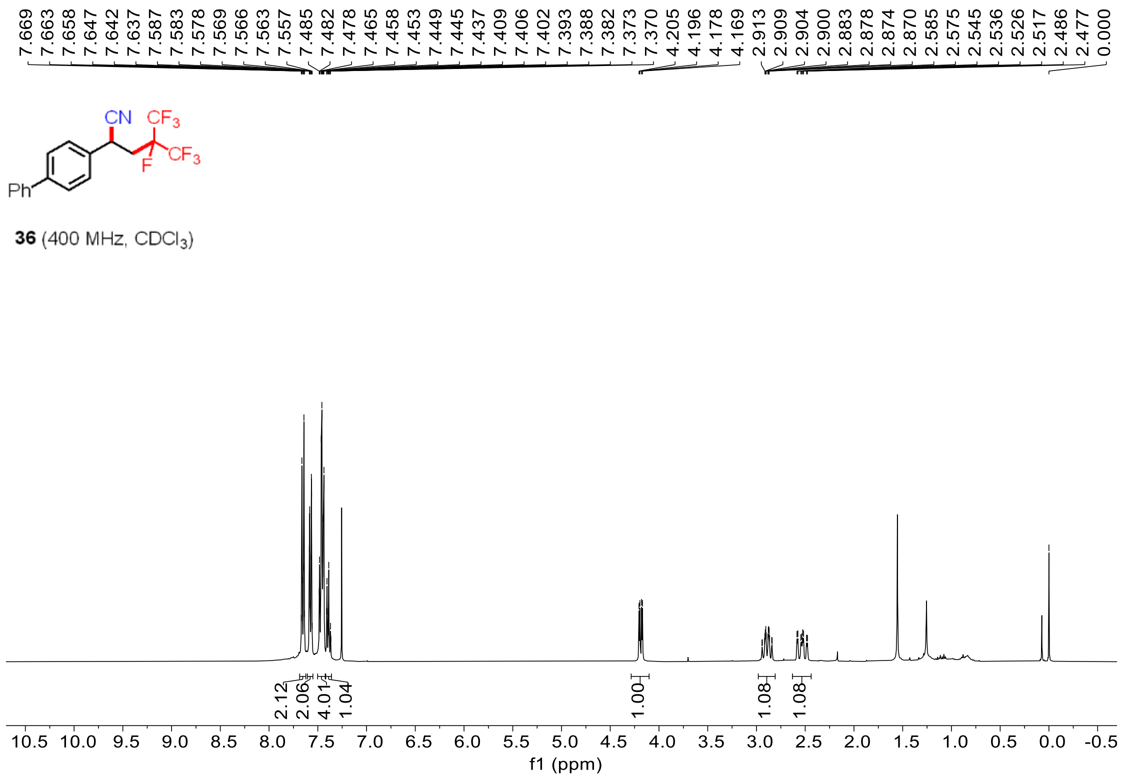
7.669 7.663 7.658 7.647 7.642 7.637 7.587 7.583 7.578 7.569 7.566 7.563 7.557 7.485 7.482 7.478 7.465 7.458 7.453 7.449 7.445 7.437 7.409 7.406 7.402 7.393 7.388 7.382 7.373 7.370 4.205 4.196 4.178 4.169 2.913 2.909 2.904 2.900 2.883 2.878 2.874 2.870 2.585 2.575 2.545 2.536 2.526 2.517 2.486 2.477 0.000
CN
CF3
CF3
F
Ph
36 (400 MHz, CDCl3)
2.12 2.06 4.01 1.04 1.00 1.08 1.08
f1 (ppm)

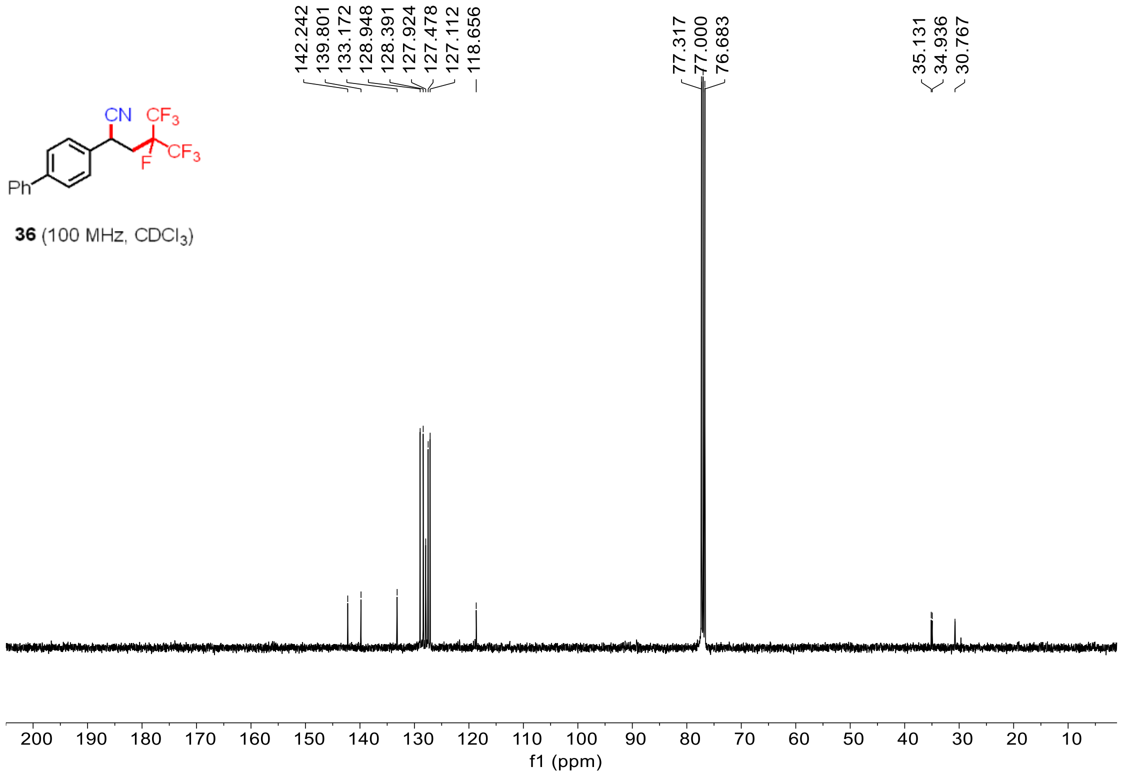
142.242 139.801 133.172 128.948 128.391 127.924 127.478 127.112 118.656 77.317 77.000 76.683 35.131 34.936 30.767
CN
CF3
CF3
F
Ph
36 (100 MHz, CDCl3)
f1 (ppm)

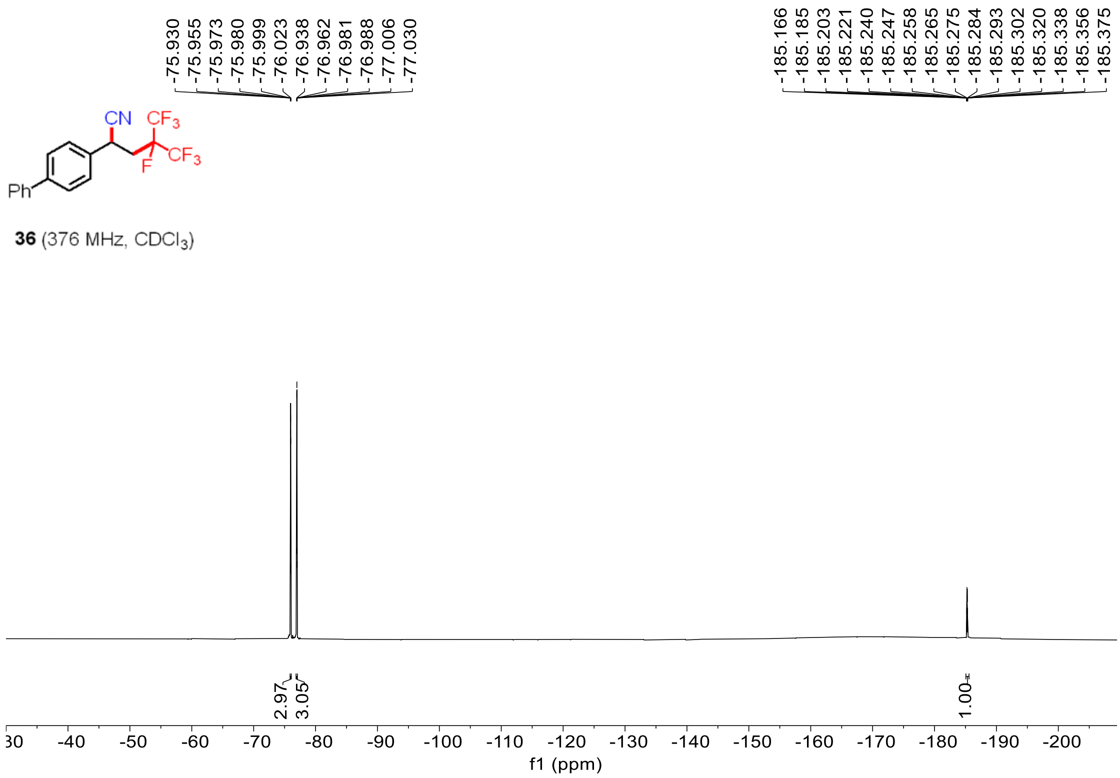

36 (376 MHz, $CDCl_3$)

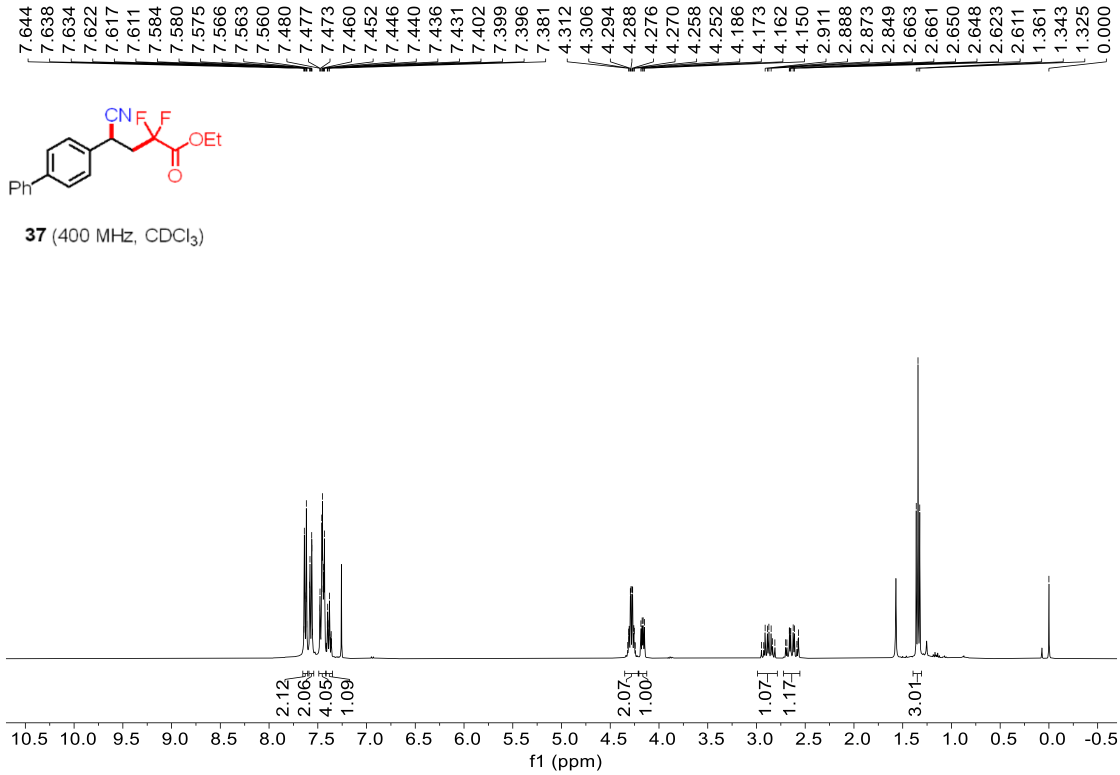

37 (400 MHz, $CDCl_3$)

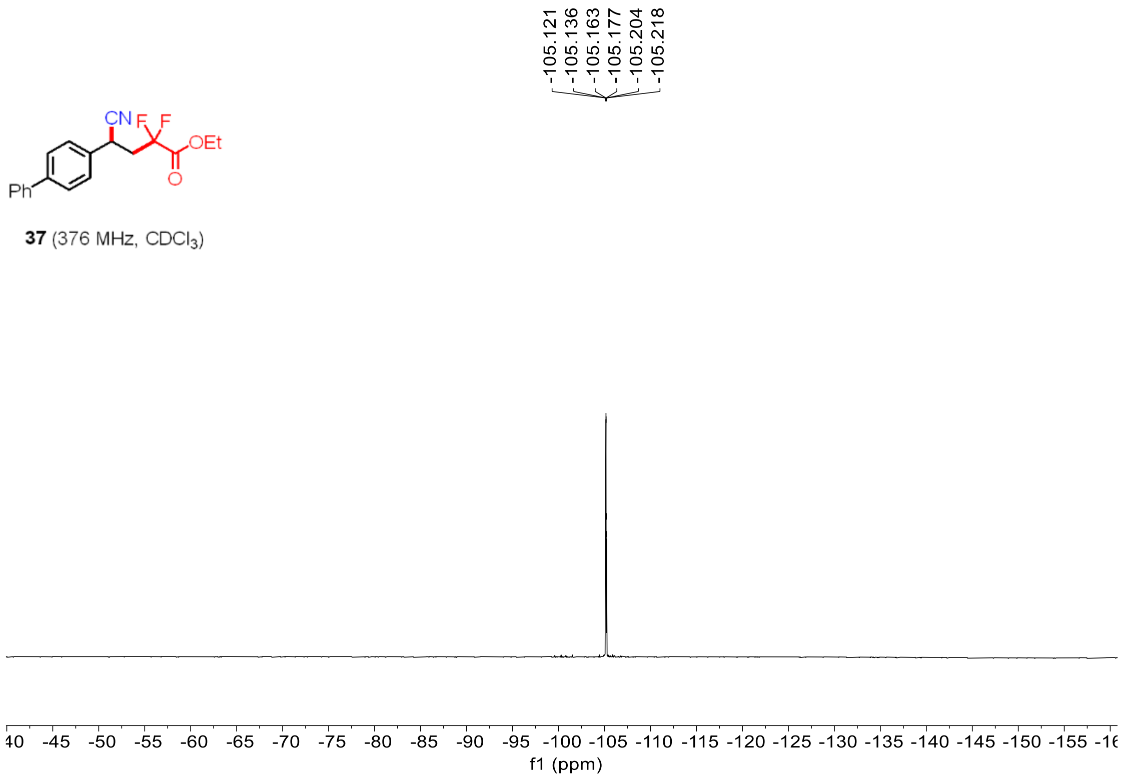

37 (376 MHz, $CDCl_3$)

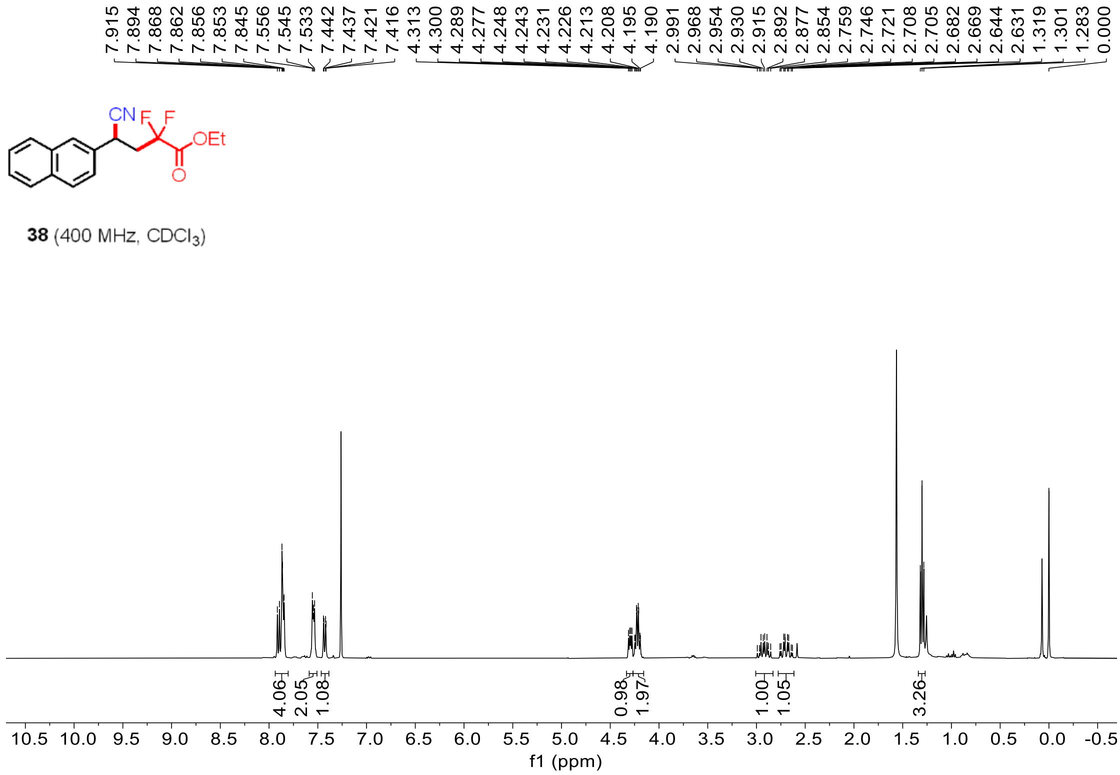

38 (400 MHz, $CDCl_3$)

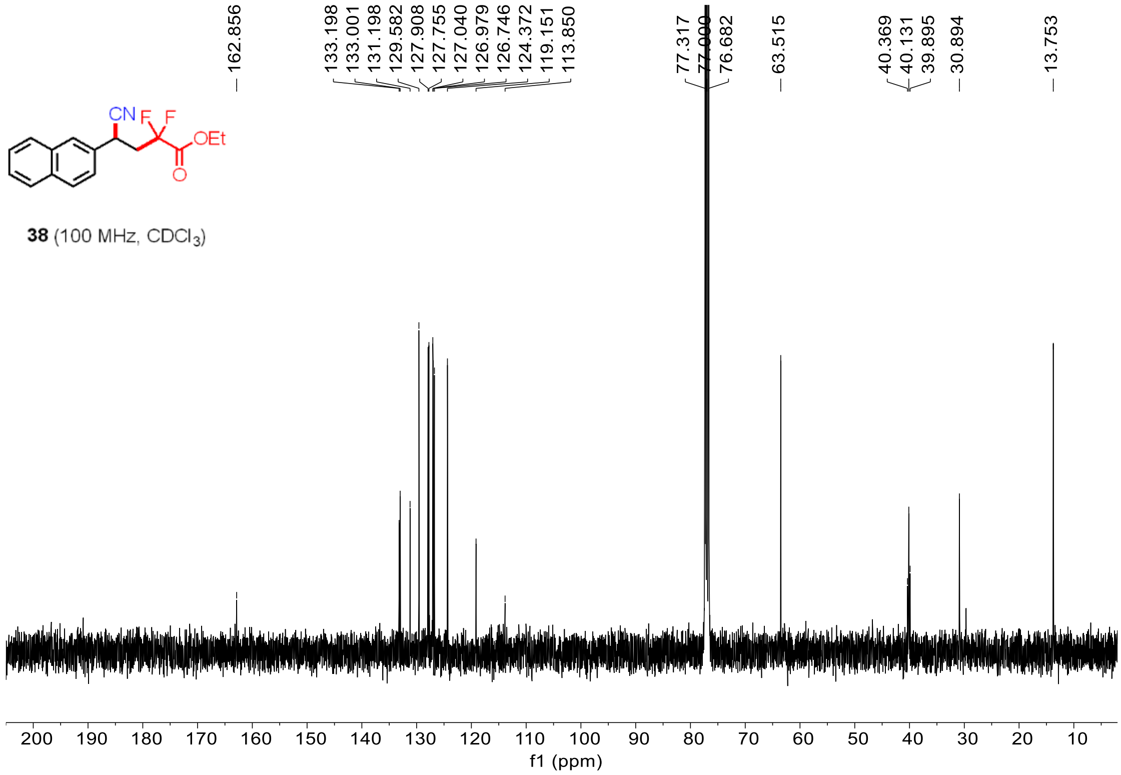

38 (100 MHz, $CDCl_3$)

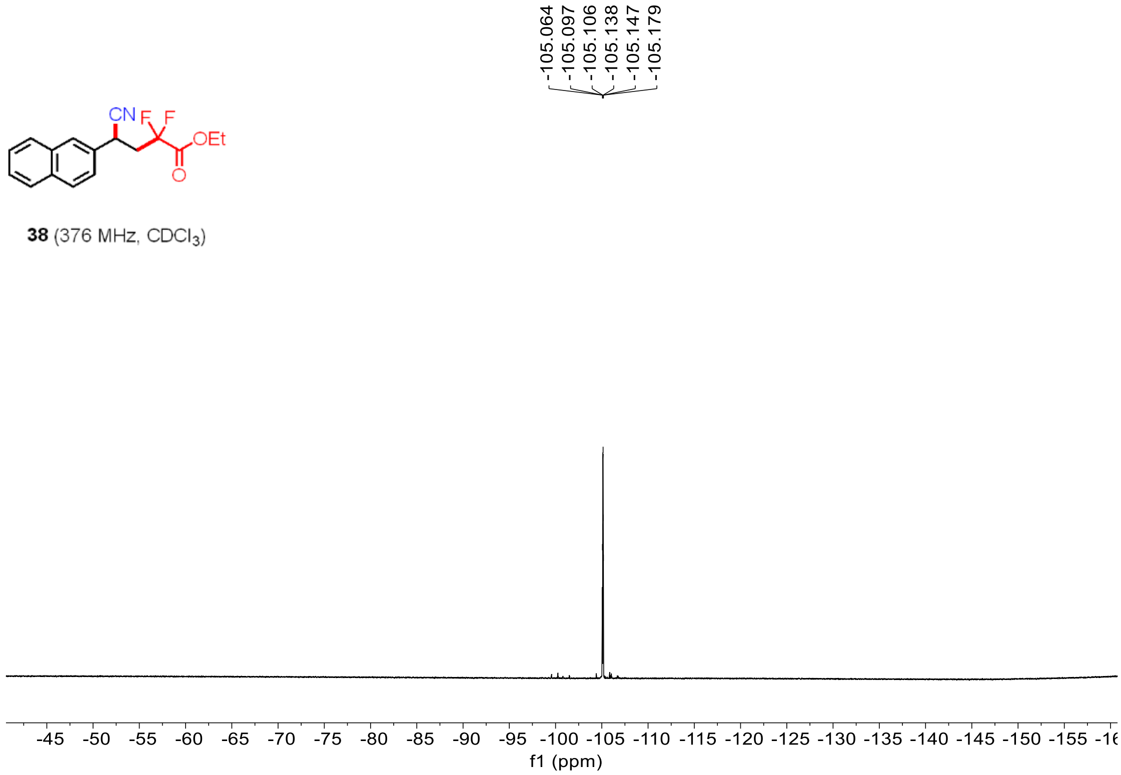

38 (376 MHz, $CDCl_3$)

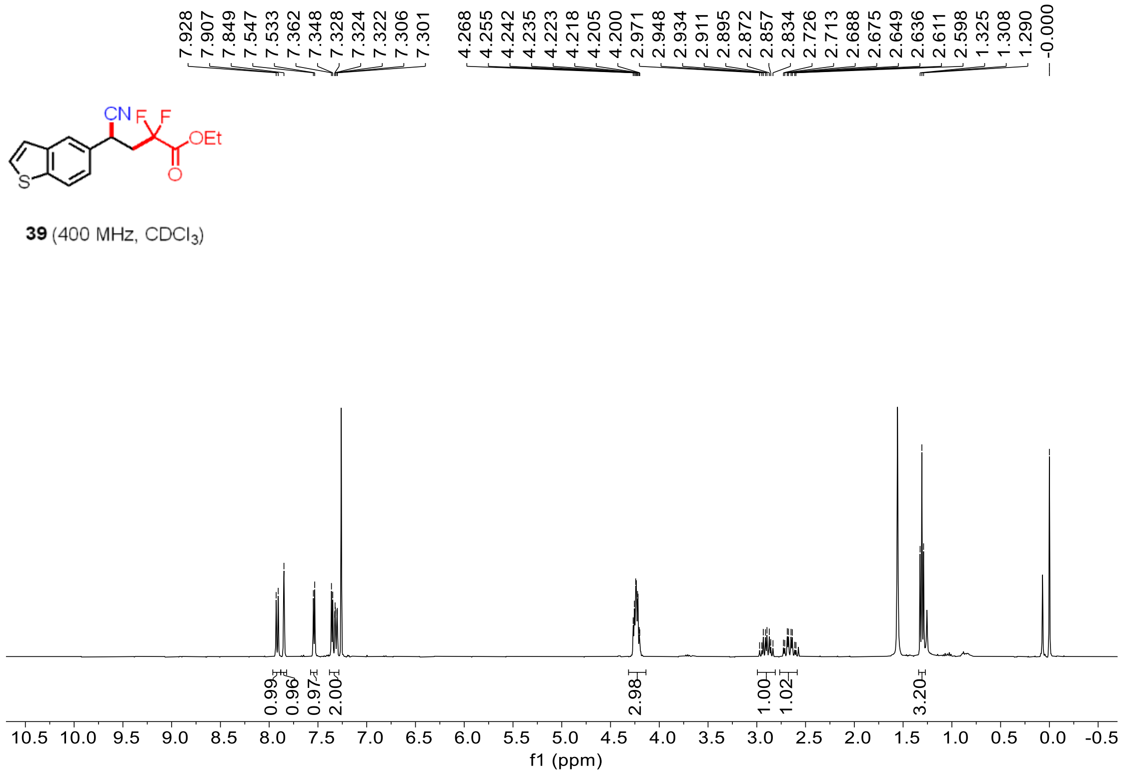

39 (400 MHz, $CDCl_3$)

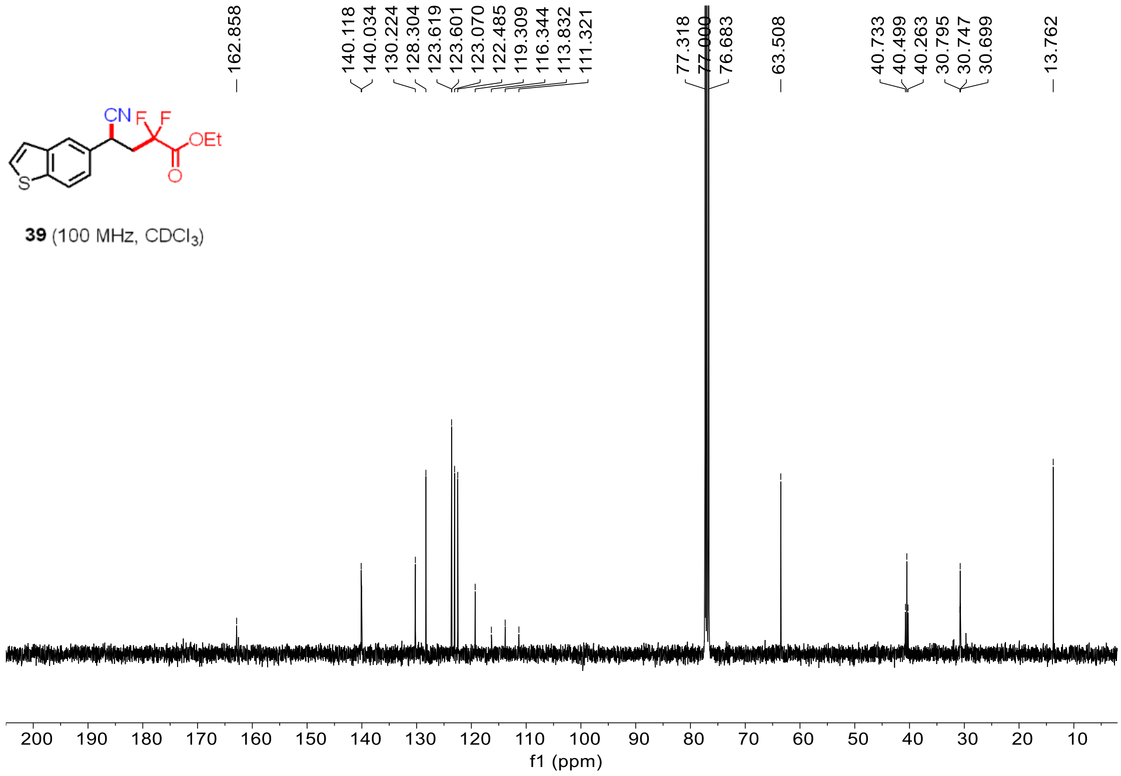

39 (100 MHz, $CDCl_3$)

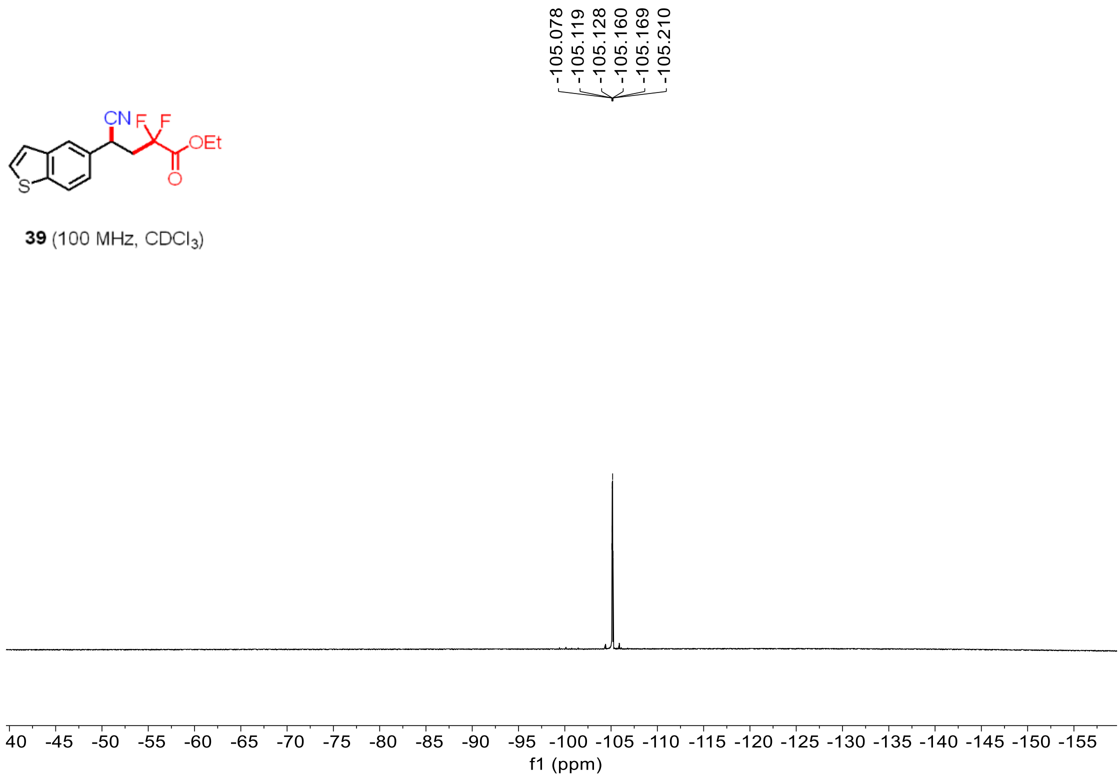

39 (100 MHz, $CDCl_3$)

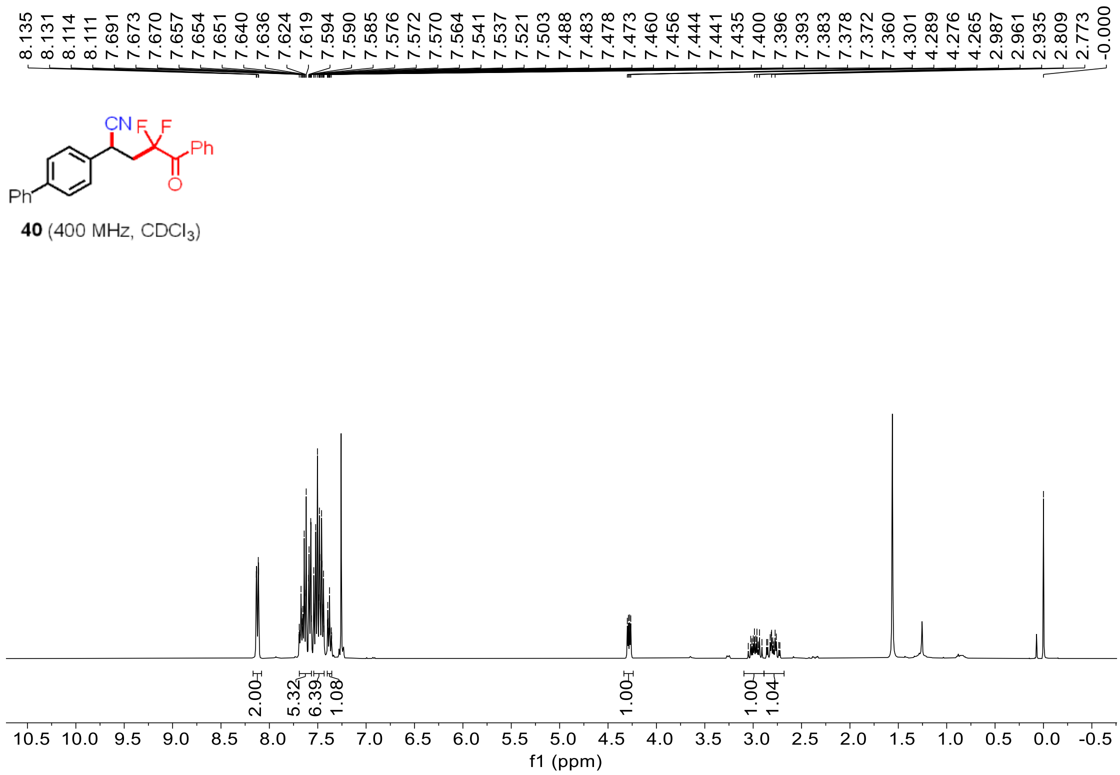

40 (400 MHz, $CDCl_3$)

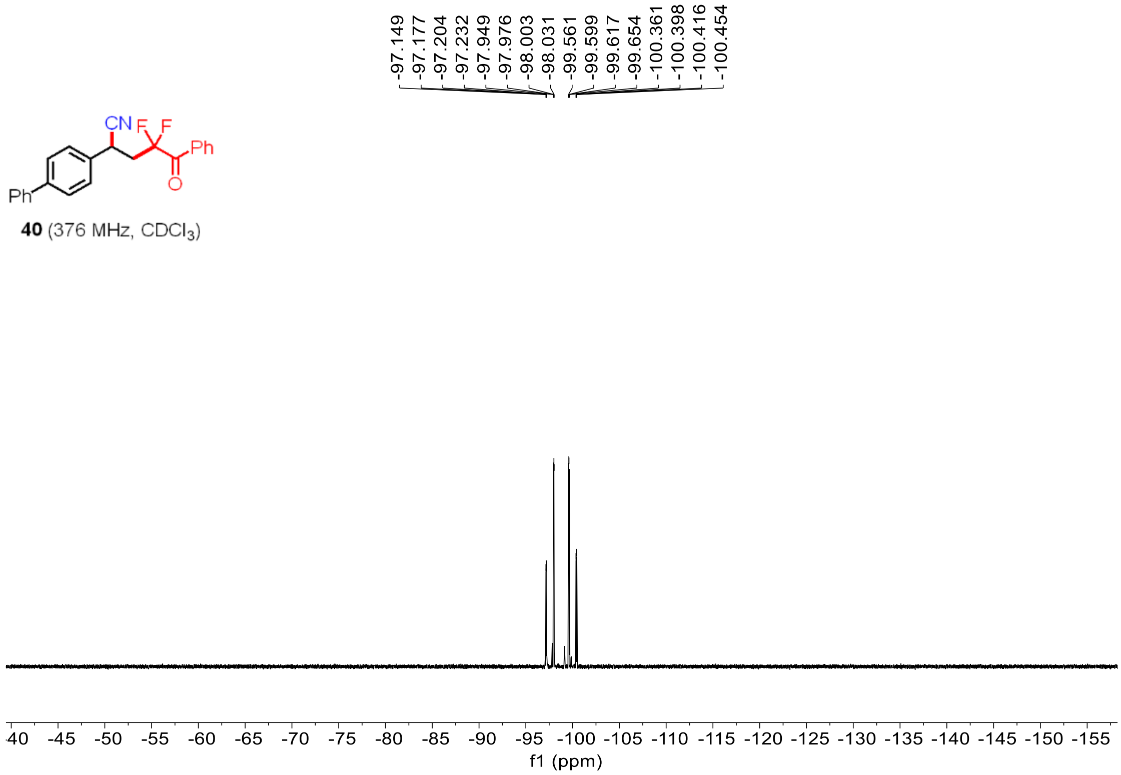
-97.149
-97.177
-97.204
-97.232
-97.949
-97.976
-98.003
-98.031
-99.561
-99.599
-99.617
-99.654
-100.361
-100.398
-100.416
-100.454
CN F F
Ph
O
Ph
40 (376 MHz, CDCl3)
40 -45 -50 -55 -60 -65 -70 -75 -80 -85 -90 -95 -100 -105 -110 -115 -120 -125 -130 -135 -140 -145 -150 -155
f1 (ppm)

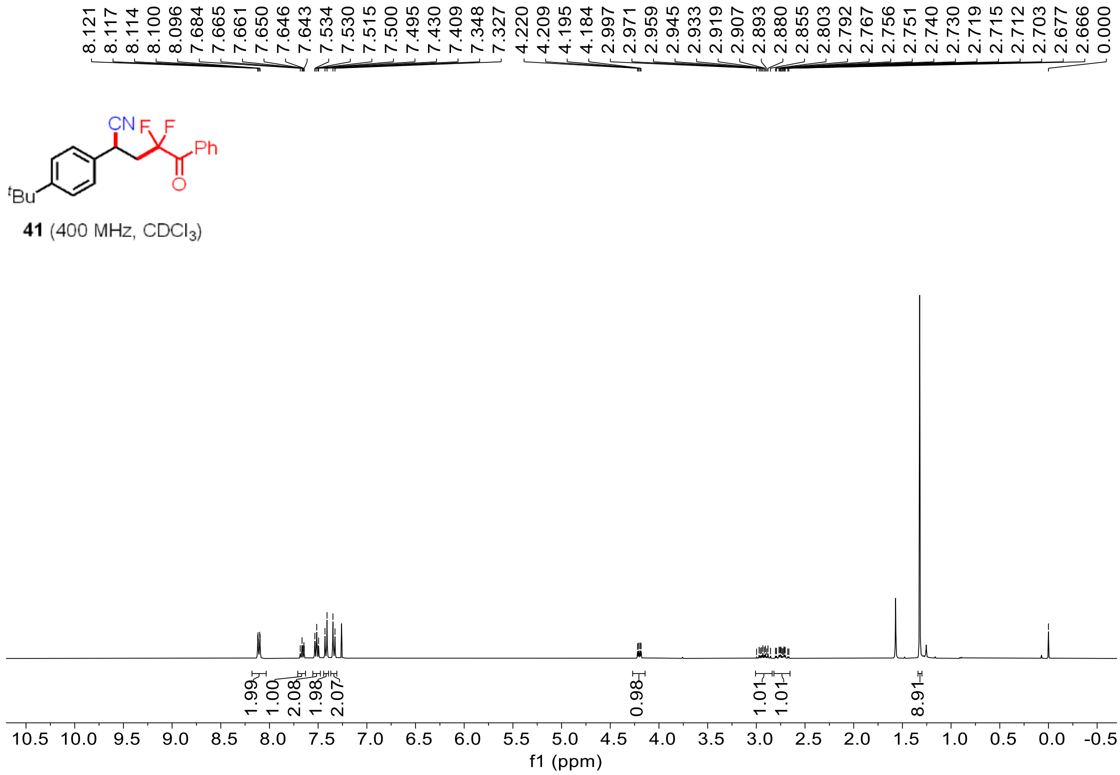
8.121
8.117
8.114
8.100
8.096
7.684
7.665
7.661
7.650
7.646
7.643
7.534
7.530
7.515
7.500
7.495
7.430
7.409
7.348
7.327
4.220
4.209
4.195
4.184
2.997
2.971
2.959
2.945
2.933
2.919
2.907
2.893
2.880
2.855
2.803
2.792
2.767
2.756
2.751
2.740
2.730
2.719
2.715
2.712
2.703
2.677
2.666
0.000
CN F F
Ph
O
tBu
41 (400 MHz, CDCl3)
1.99
1.00
2.08
1.98
2.07
0.98
1.01
1.01
8.91
10.5 10.0 9.5 9.0 8.5 8.0 7.5 7.0 6.5 6.0 5.5 5.0 4.5 4.0 3.5 3.0 2.5 2.0 1.5 1.0 0.5 0.0 -0.5
f1 (ppm)

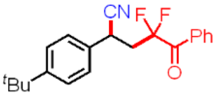

41 (376 MHz, $CDCl_3$)

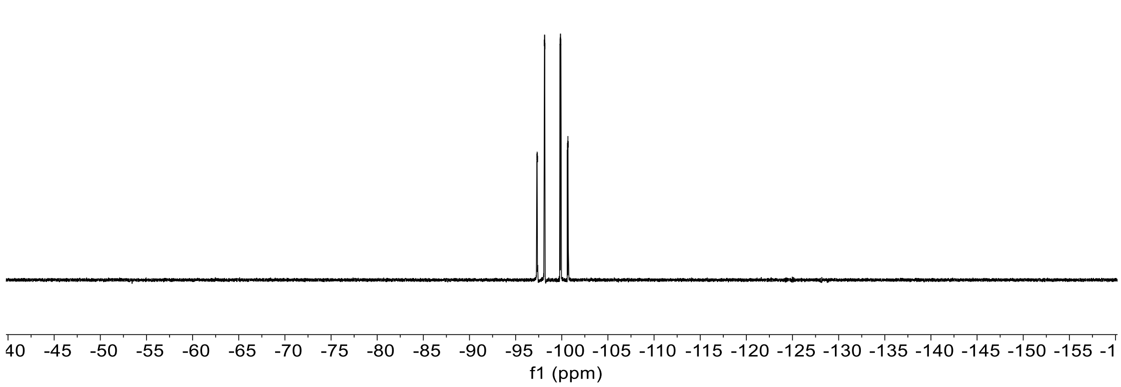

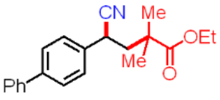

42 (500 MHz, $CDCl_3$)

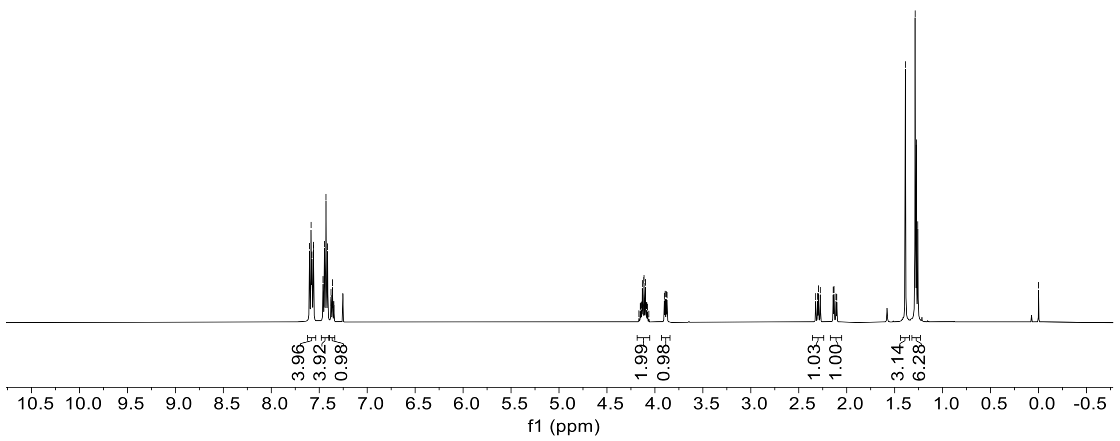

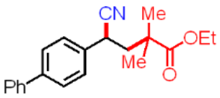

42 (125 MHz, $CDCl_3$)

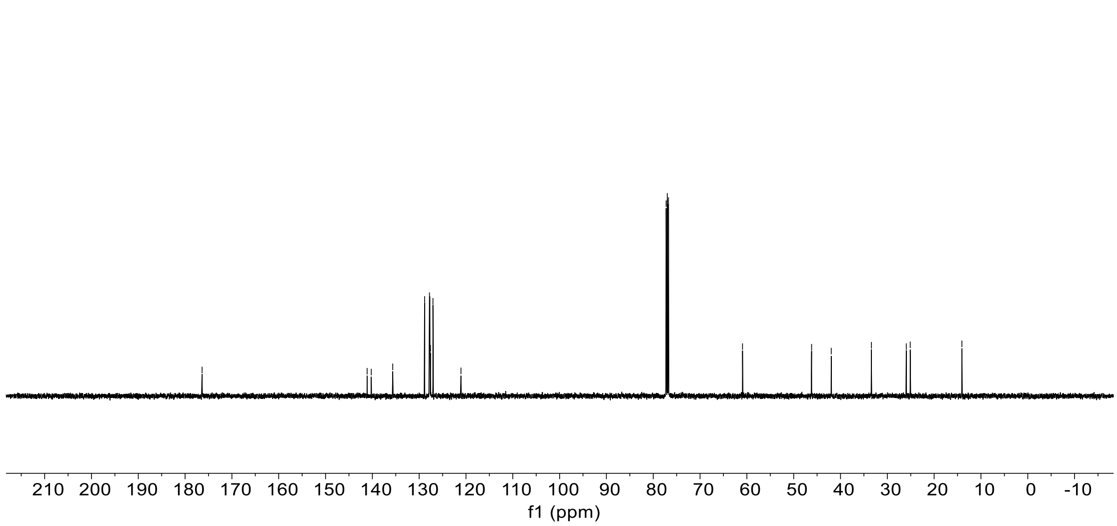

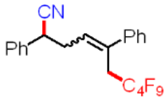

43 (500 MHz, $CDCl_3$)

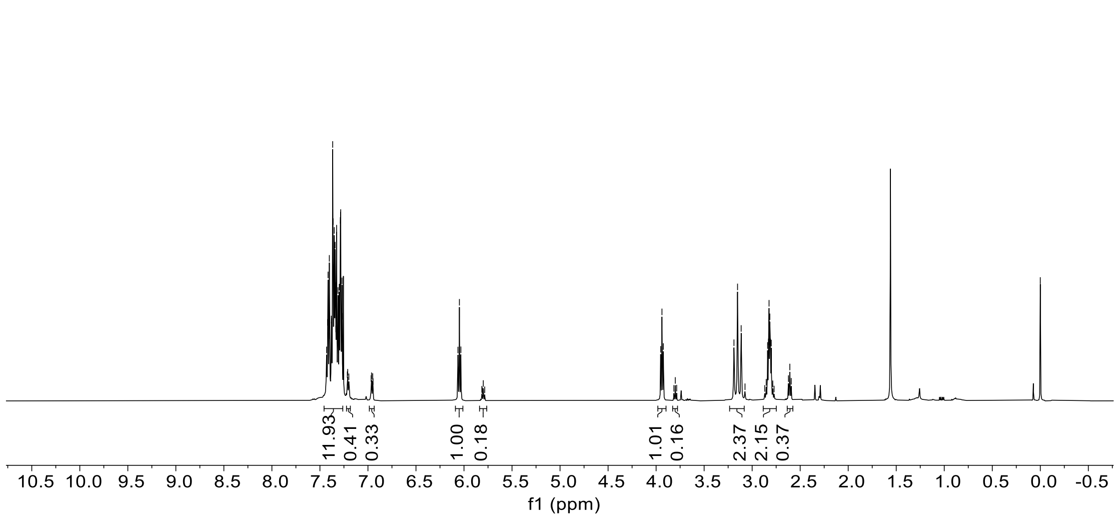

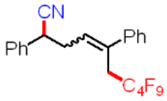

43 (125 MHz, $CDCl_3$)

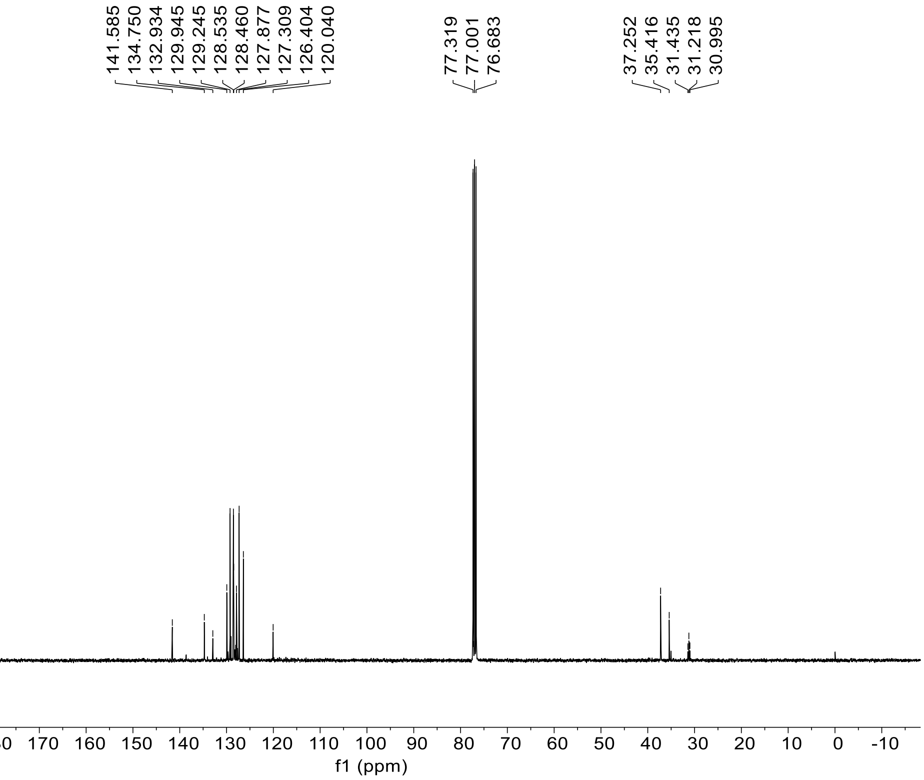

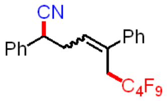

43 (471 MHz, $CDCl_3$)

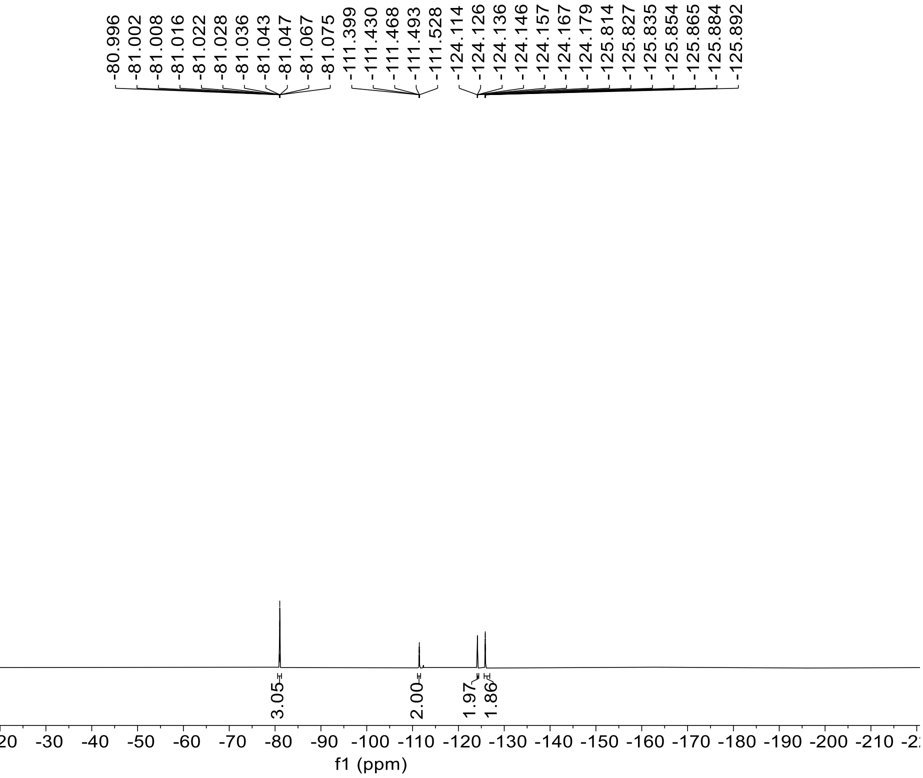

NOESY spectra of compound **43**:

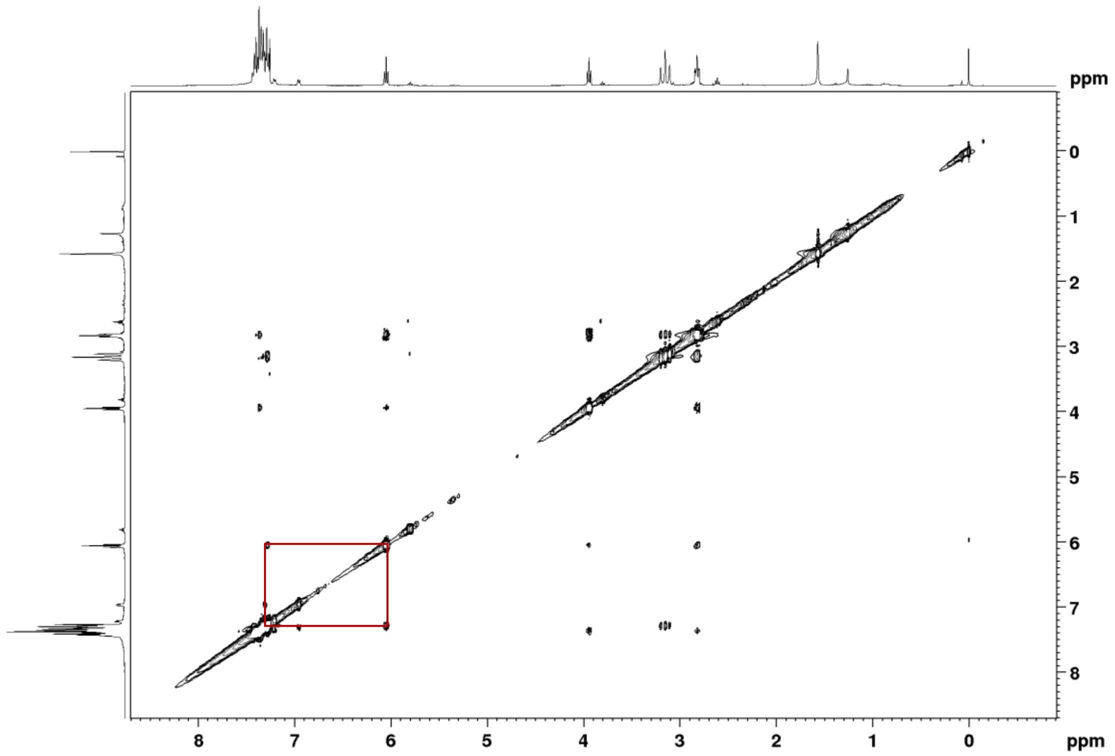

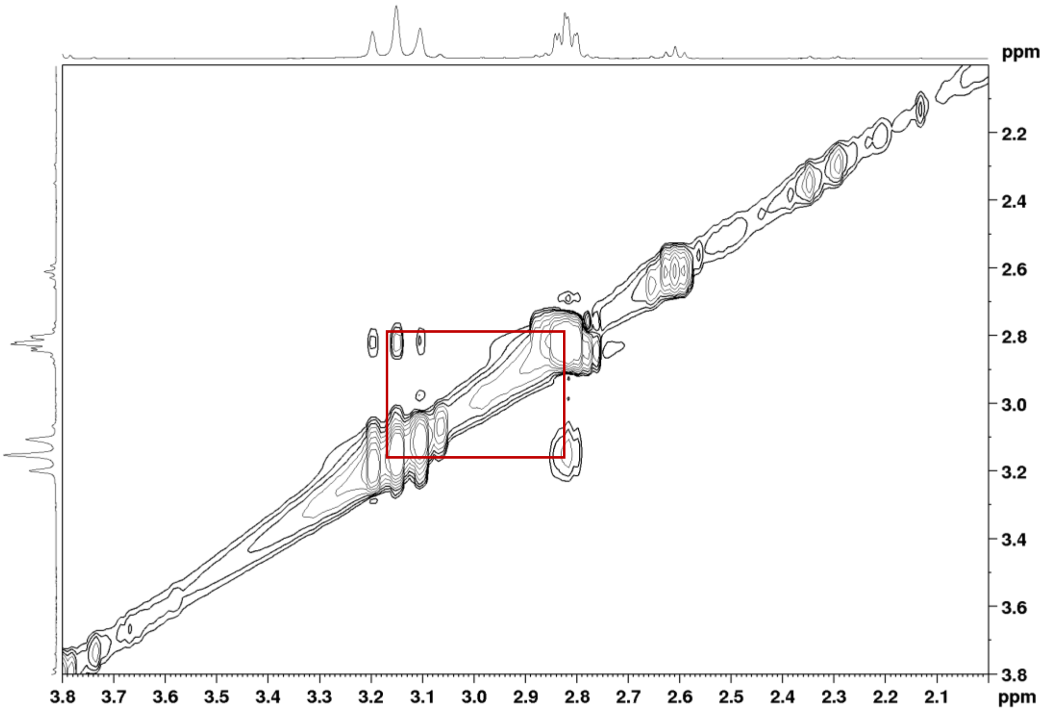

Supplement: Supplementary file 1 — Supplementary Information [file 41467_2024_49081_MOESM1_ESM.pdf]
